# Construction of a Multitissue Cell Atlas Reveals Cell‐Type‐Specific Regulation of Molecular and Complex Phenotypes in Pigs

**DOI:** 10.1002/advs.202504961

**Published:** 2025-11-27

**Authors:** Lijuan Chen, Houcheng Li, Jinyan Teng, Zhen Wang, Xiaolu Qu, Zhe Chen, Xiaodian Cai, Haonan Zeng, Zhonghao Bai, Jinghui Li, Xiangchun Pan, Leyan Yan, Fei Wang, Lin Lin, Yonglun Luo, Goutam Sahana, Mogens Sandø Lund, Maria Ballester, Daniel Crespo‐Piazuelo, Peter Karlskov‐Mortensen, Merete Fredholm, Alex Clop, Marcel Amills, Crystal Loving, Christopher K. Tuggle, Ole Madsen, Jiaqi Li, Zhe Zhang, George E. Liu, Jicai Jiang, Lingzhao Fang, Guoqiang Yi

**Affiliations:** ^1^ Shenzhen Branch Guangdong Laboratory of Lingnan Modern Agriculture, Key Laboratory of Livestock and Poultry Multi‐omics of MARA Kunpeng Institute of Modern Agriculture at Foshan Agricultural Genomics Institute at Shenzhen Chinese Academy of Agricultural Sciences Shenzhen 518124 China; ^2^ Center for Quantitative Genetics and Genomics Aarhus University Aarhus 8000 Denmark; ^3^ State Key Laboratory of Livestock and Poultry Breeding National Engineering Research Center for Breeding Swine Industry Guangdong Provincial Key Lab of Agro‐Animal Genomics and Molecular Breeding College of Animal Science South China Agricultural University Guangzhou 510642 China; ^4^ Key Laboratory for Crop and Animal Integrated Farming Ministry of Agriculture and Rural Affairs Institute of Animal Science Jiangsu Academy of Agricultural Sciences Nanjing 210014 China; ^5^ Department of Animal Science University of California Davis CA 95616 USA; ^6^ Genetic Medicine University of Chicago Chicago IL 60637 USA; ^7^ Department of Biomedicine Aarhus University Aarhus 8000 Denmark; ^8^ Steno Diabetes Center Aarhus Aarhus University Hospital Aarhus 8200 Denmark; ^9^ Animal Breeding and Genetics Program Institut de Recerca i Tecnologia Agroalimentàries (IRTA) Torre Marimon Caldes de Montbui Barcelona 08140 Spain; ^10^ Animal Genetics and Breeding Department of Veterinary and Animal Sciences University of Copenhagen Frederiksberg C 1870 Denmark; ^11^ Department of Animal Genetics Centre for Research in Agricultural Genomics (CRAG) CSIC‐IRTA‐UAB‐UB Campus de la Universitat Autònoma de Barcelona Bellaterra 08193 Spain; ^12^ Departament de Ciència Animal i dels Aliments Universitat Autònoma de Barcelona Bellaterra 08193 Spain; ^13^ USDA‐ARS‐National Animal Disease Center Ames IA 50011 USA; ^14^ Department of Animal Science Iowa State University Ames IA 50011 USA; ^15^ Animal Breeding and Genomics Wageningen University & Research PO Box 338 Wageningen 6700 AH The Netherlands; ^16^ Animal Genomics and Improvement Laboratory Henry A. Wallace Beltsville Agricultural Research Center Agricultural Research Service Agricultural Research Service USDA Beltsville Maryland 20705 USA; ^17^ Department of Animal Science North Carolina State University Raleigh NC 27695 USA; ^18^ Bama Yao Autonomous County Rural Revitalization Research Institute Bama 547500 China

**Keywords:** cell‐type‐trait association, cellular deconvolution, gene regulation, PigGTEx, single‐nucleus RNA‐seq

## Abstract

The systematic characterization of cellular heterogeneity among tissues and cell‐type‐specific regulation underlying complex phenotypes remains elusive in pigs. Within the Pig Genotype‐Tissue Expression (PigGTEx) project, this work presents a single‐cell transcriptome atlas of adult pigs encompassing 229 268 high‐quality nuclei from 19 tissues, annotated to 67 major cell types. Besides cellular heterogeneity within and across tissues, this work further characterizes prominent tissue‐specific features and functions of muscle, epithelial, and immune cells. Through deconvoluting 3921 bulk RNA‐seq samples from 17 matching tissues, this work dissects thousands of genetic variants with cell‐type interaction effects on gene expression (ieQTL). By colocalizing these ieQTL with variants associated with 268 complex traits, new insights into the cellular mechanisms behind these traits are provided. Moreover, this work highlights that orthologous genes with cell‐type‐specific regulation in pigs exhibit significant heritability enrichment for some human complex phenotypes. Altogether, this work provides a valuable resource and highlights novel insights in cellular regulation of complex traits for accelerating pig precision breeding and human biomedical research.

## Graphical abstract

This research conducts an in‐depth investigation of cell‐type‐specific regulatory mechanisms underlying molecular and complex phenotypes through integrative analysis of multitissue single‐nucleus RNA sequencing, bulk RNA‐seq, and genome‐wide association study (GWAS) data in pigs. This pioneering study establishes a valuable genomic resource that significantly enhances pig precision breeding while simultaneously advancing human biomedical research.

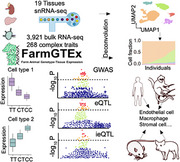

## 1 Introduction

The cell is a fundamental structural, biological, and evolutionary unit of life and plays a key role in orchestrating the development and homeostasis of all living beings through global intercellular interactions. Multicellular organisms, including mammals, are generally composed of over 400 distinct cell types that are distinct in morphology and function.^[^ ^1^, ^2^, ^3^, ^4^, ^5^ ^]^ Genome‐wide association studies (GWAS) have revealed that over 90% of phenotype‐associated genetic variants lie within noncoding regions, suggesting that these variants might influence complex phenotypes through gene expression modulation.^[^ ^6^, ^7^, ^8^ ^]^ The limited overlaps between bulk expression quantitative trait loci (eQTL) and GWAS signals suggest that many candidate variants might regulate biological processes and then complex phenotypes through cell‐type‐specific mechanisms.^[^ ^9^, ^10^, ^11^, ^12^ ^]^ Single‐cell omics studies have shown that the substantial disorders in cellular activity, identity, and composition play a crucial role in the development of complex traits and diseases, both within and across individuals,^[^ ^5^, ^13^, ^14^, ^15^, ^16^ ^]^ highlighting the importance of constructing a multitissue single‐cell atlas for functionally understanding genotype‐phenotype associations. In addition, a better understanding of molecular and cellular mechanisms underpinning complex phenotypes will be an important initial step in generating new avenues for precision breeding in agriculture and therapeutic solutions for similar human diseases.^[^ ^13^, ^14^, ^17^ ^]^

As an important farm animal species, the domestic pig (*Sus scrofa*) is not only an abundant source of animal protein worldwide but also serves as a valuable human biomedical model and an optimal organ donor for xenotransplantation.^[^ ^18^ ^]^ Numerous studies in pigs have delineated significant QTL underlying complex traits of economic importance,^[^ ^19^, ^20^ ^]^ leading to vast improvements in pig breeding programs and production efficiency. However, the systematic interpretation of molecular mechanisms underlying complex phenotypes in pigs lags behind human and mouse research due to limitations in functional data availability. The ongoing Functional Annotation of Animal Genomes (FAANG) and Farm animal Genotype‐Tissue Expression projects (FarmGTEx) are global efforts to provide catalogues of functional elements and variants in pigs at tissue level.^[^ ^21^, ^22^, ^23^ ^]^ The next step is to explore the cell‐type‐dependent biological consequences of trait‐associated variants as tissues contain numerous cell types.^[^ ^24^ ^]^ Although some studies have conducted single‐cell/nucleus RNA‐seq (scRNA‐seq and snRNA‐seq) analyses in pigs, they primarily focused on elucidating the cellular heterogeneity and trajectories of lineage specification in a limited range of tissue types.^[^ ^25^, ^26^, ^27^, ^28^, ^29^, ^30^, ^31^, ^32^ ^]^ The cell‐type‐specific biological impacts of genetic variants on complex traits by integrating single‐cell RNA‐sequencing with large‐scale pig genetics data still need to be explored.

To further fine‐map the causative genetic variants and decipher their cellular impacts on both molecular and complex phenotypes in pigs, we first constructed a single‐nucleus transcriptome atlas by profiling a total of 319, 433 nuclei from 19 major tissue types, representing 261 major cell clusters. Dissection of muscle, epithelial, and immune cells depicted the cellular heterogeneity across these tissues and revealed a number of critical master regulators (i.e., GATA4 and ZBTB11) driving cell identity. Through cellular deconvolution of PigGTEx tissues, cell‐type interaction expression QTL (ieQTL) mapping, and the integrative analysis with GWAS results of 268 complex traits, we pinpoint the cell‐type‐specific contexts in which trait‐associated genetic variants regulate the transcriptional activity and result in phenotypic variation. Moreover, we demonstrate that orthologous genes with cell‐type‐specific regulation in pigs exhibit significant heritability enrichment for many human complex phenotypes. Overall, this study enriches and enhances rich and open resources (http://piggtex.farmgtex.org/ and https://dreamapp.biomed.au.dk/pigatlas/) for charting the cell–cell transcriptome variability within and across tissues and expands our understanding of the connections between genetic variants and phenotypes at single‐cell resolution in pigs. Our results provide relevant information for the development of future precision breeding strategies in pigs and human biomedical research.

## 2 Results

### 2.1 Global Landscape of Single‐Nucleus Transcriptomic Reference Atlas from 19 Pig Tissues

**Figure 1:**
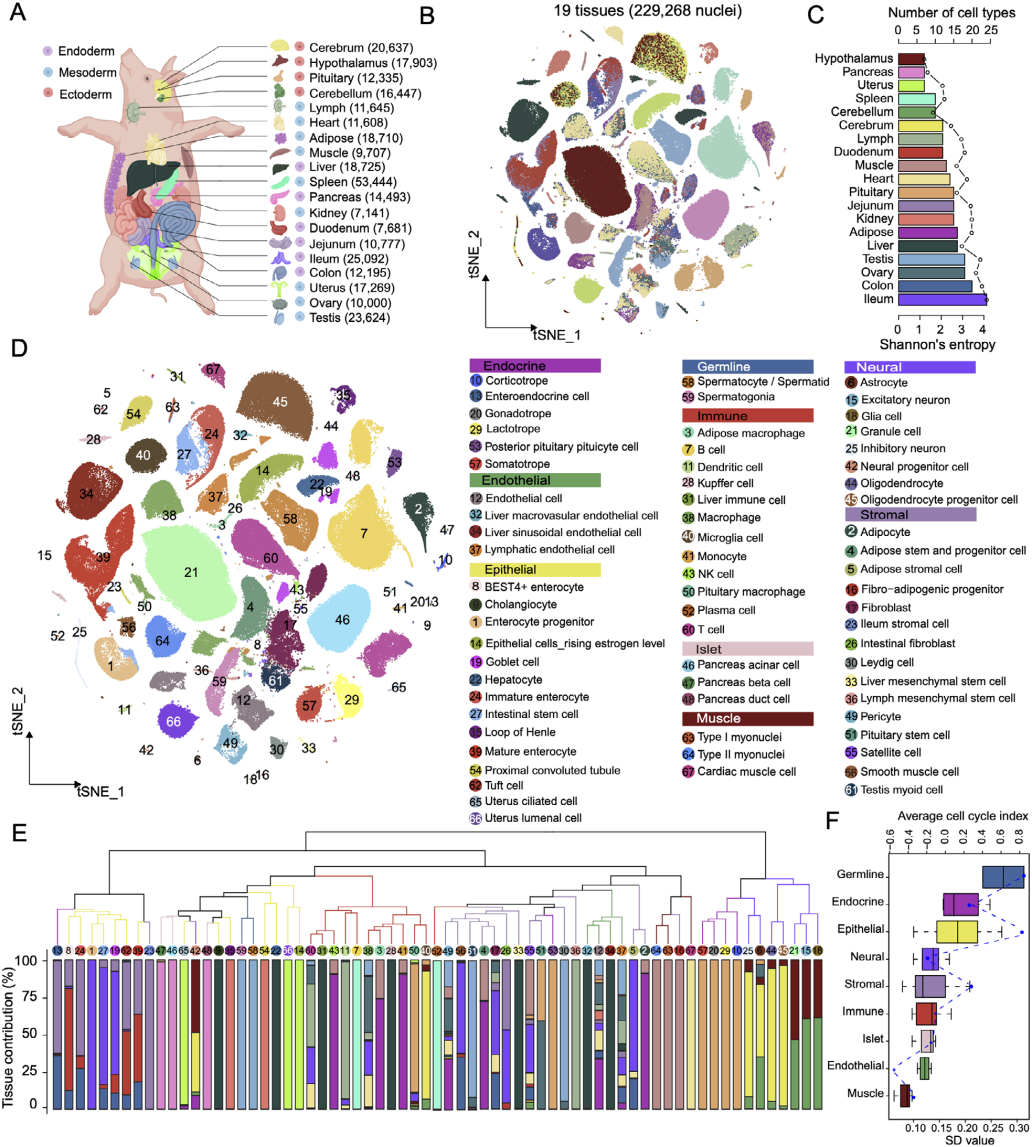
Single‐nucleus transcriptomic landscape across 19 frozen tissues in adult pigs. A) Schematic diagram showing 19 primary pig tissues collected for snRNA‐seq in this study. The cartoons used to generate this illustration were purchased from BioRender.com. The number of nuclei profiled per tissue is denoted in parentheses. B) t‐Distributed Stochastic Neighbor Embedding (t‐SNE) visualization of single‐nucleus profiles (dots) colored by tissues. C) Bar plot displaying the number and diversity of cell types identified in each of the 19 tissues. Entropy shown by dotted line was calculated as described in Experimental Section. D) t‐SNE visualization of single‐nucleus profiles (dots) colored by major cell types. All cell types are categorized into nine top‐level cell lineages, and cell‐type annotation is provided in the legend to the right. E) Cellular relationship and composition across tissues. The dendrogram was created by hierarchical clustering based on the transcriptional levels of each cell type. The bar chart represents the relative contributions of tissues to each cell type. The branch is colored by the lineage based on the classification to which the cell type belongs. F) Cell state prediction of nine top‐level cell lineages. Cells with higher cell cycling index are more proliferative. The top represents the average cell‐cycle index values. The horizontal line in the boxplots corresponds to the median, the box bounds indicate the 25th and 75th percentiles and the whiskers represent 1.5 times the interquartile range. Values outside the whiskers are displayed as points. The bottom represents the standard deviation (SD) values, which were evaluated for the variations in the cell states of each lineage. The blue dots represent the SD values.

To generate a comprehensive multitissue single‐cell transcriptomic reference atlas of pigs, we performed snRNA‐seq in 19 tissues/organs from two adult Meishan pigs (one male and one female) using 10× Genomics technology, including subcutaneous adipose, cerebellum, cerebrum, colon, duodenum, heart, hypothalamus, ileum, jejunum, kidney, liver, lymph node, skeletal muscle, ovary, pancreas, pituitary gland, spleen, testis, and uterus (**Figure** **1**A). An overview of the study workflow was presented to facilitate a clear understanding of the research (Figure S1, Supporting Information). Initially, we profiled 16 812 nuclei and sequenced over 660 million raw reads per tissue on average (Figure 1A). After quality control (see Experimental Section for details), we obtained transcriptomic data for a total of 229 268 high‐quality nuclei across all 19 tissues (Figure S2, Supporting Information). All tissues showed sufficient transcriptional abundance, with a median of 4008 unique molecular identifiers (UMI) and 2064 transcribed genes per nucleus, therefore displaying higher expression than the previously reported single‐cell data in pigs.^[^ ^27^ ^]^ Subsequently, we evaluated the integration of snRNA‐seq data across 19 tissues, and observed that Scanorama obtained the highest overall scores in biological conservation and batch correction^[^ ^33^ ^]^ among the four single‐cell integration tools (Scanorama,^[^ ^34^ ^]^ principal component analysis (PCA),^[^ ^35^ ^]^ ComBat,^[^ ^36^ ^]^ and Harmony^[^ ^37^ ^]^) (Figure S3, Supporting Information). We first assessed the transcriptional similarity by comparing our snRNA‐seq data with that from a previous study across seven common tissues.^[^ ^27^ ^]^ The Spearman correlation values between the two pseudo‐bulk single‐cell transcriptomic profiles were high for all tissues, ranging from 0.653 to 0.825 (Figure S4, Supporting Information), suggesting globally consistent transcriptional profiles of samples between these two single‐cell RNA‐seq studies at the bulk tissue level. The complete snRNA‐seq dataset was grouped into 77 cell clusters and manually annotated as 67 major cell types based on the expression of canonical marker genes from the literature (Figure 1B–D; Figures S5 and S6 and Tables S1 and S2, Supporting Information). To evaluate data representation and reproducibility, we generated new snRNA‐seq data from two additional liver samples. Integration of this new snRNA‐seq data with our liver data displayed high consistency in cell types across all three samples (Figure S7, Supporting Information). Furthermore, we combined the two single‐cell datasets to construct a more comprehensive cell map (Figures S8 and S9, Supporting Information). We found high matching for the majority of cell types in the integrated atlas, and systematic comparison between the two cell maps revealed high similarity in the cellular landscape, marker genes, and transcriptomic dynamics (Figures S10 and S11, Supporting Information). To alleviate the negative impacts on subsequent results by hidden batch effects, which were potentially introduced by technical variations such as different operating technicians and experimental protocols between the two studies, we decided to use the cell atlas from our dataset for the following analyses.

The global cell atlas revealed that a majority of cell types, such as cardiomyocytes, enterocytes, and hepatocytes, exhibited a high tissue specificity regarding gene expression (Figure 1D), reflecting their specialized functions. Notably, several prevalent cell types, such as immune cells, endothelial cells, and fibroblasts, were commonly shared among tissues. To gain a deeper understanding of cellular heterogeneity within each tissue, we individually analyzed and generated cellular visualizations in the hierarchy with Uniform Manifold Approximation and Projection (UMAP) and t‐Distributed Stochastic Neighbor Embedding (t‐SNE), resulting in an average of 14 main cell types per tissue (Figures S12–S14, Supporting Information). Of note, the ileum showed 24 putative cell subpopulations, consistent with its highest cell‐type diversity evaluated by the Shannon entropy index (Figure 1C,D and Figures S12–S14, Supporting Information). Additionally, we compared cellular signatures of tissues shared between our work and the previous study,^[^ ^27^ ^]^ and in general, found a high consistency in both cell annotation, distribution, and expression (Figure S15, Supporting Information). However, some cell types or marker genes, such as *ADIPOQ* in adipose tissue, *DOCK4* in heart, and *CD163* in liver, displayed distinct expression levels and patterns between the two studies (Figure S15, Supporting Information). This discrepancy may arise from differences in tissue sampling regions and experimental protocols. For example, Wang et al.^[^ ^27^ ^]^ adopted a protocol optimized for isolating endothelial cells, resulting in significant depletion of adipocytes in adipose tissue, thus the *ADIPOQ* gene was not detected in their work. To further probe the intercellular relationships, we conducted an unsupervised hierarchical clustering analysis for all these 67 cell types based on their transcriptomic profiles (Figure 1E). These cell types could be largely classified into nine different functional groups of cells by their biological functions based on some previous studies,^[^ ^4^, ^38^, ^39^ ^]^ including endocrine, endothelial, epithelial, germline, immune, islet, muscle, neural and stromal cells. Remarkably, we observed a higher similarity among cell types within the nine major lineages rather than among tissues by conducting within‐lineage and between‐lineage correlation analyses, suggesting that cell clustering was primarily driven by cell type and that these neighboring cell types possibly had similar functions (Figure 1E and Figure S16, Supporting Information). Moreover, we performed hierarchical clustering of pseudo‐bulk across 19 tissues. We observed four major clades (e.g., the digestive, neural, muscle, and immune systems), as well as other separate groups (Figure S17, Supporting Information). Notably, the digestive groups, which included four intestinal segments, exhibited highly similar gene expression profiles. Similarly, the hypothalamus, cerebellum, cerebrum, and pituitary clustered together within the neural systems. These results suggest that functionally similar tissues possess similar gene expression patterns. To evaluate the dynamics of cell state in each cell type, we computed the cell cycling index as described previously.^[^ ^3^ ^]^ Germline cells exhibited a greater cell division capacity than other cells, while the endothelial, stromal, and muscle compartments, which are known to be largely quiescent, had low cycling indices (Figure 1F and Figure S18, Supporting Information). Notably, the epithelial cells presented the highest variations which are evaluated by the standard deviation (SD) in cell states besides the germline cells (only two data points), suggesting great functional diversity of epithelial cell subpopulations.

### 2.2 Distinct Transcriptional Patterns Among Three Types of Muscle Cells

**Figure 2:**
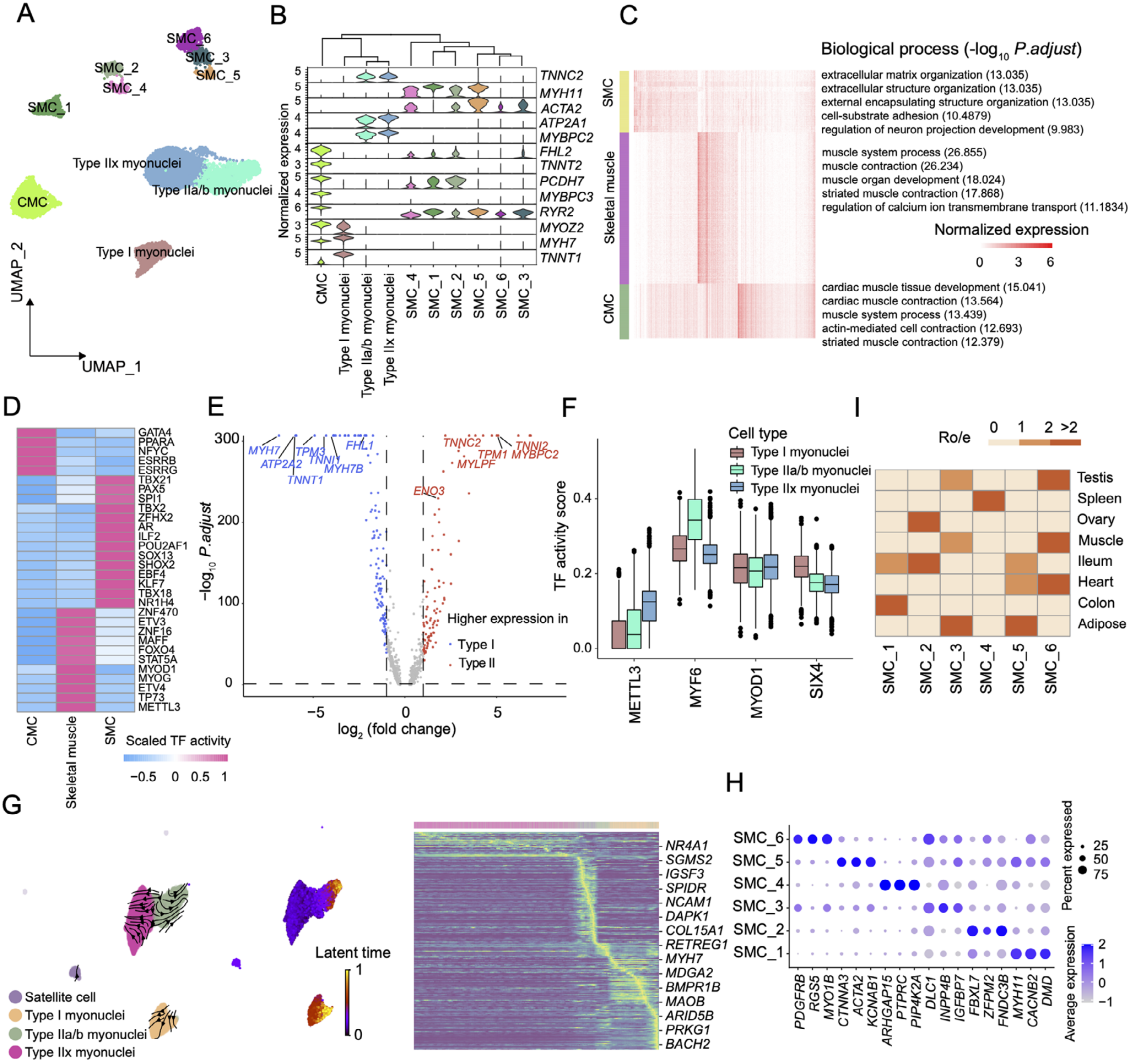
Identification and characterization of three muscle cell types. A) Uniform Manifold Approximation and Projection (UMAP) visualization of all muscle cells from eight tissues. Each dot represents one nucleus, with colors coded according to manually annotated cell types. CMC, cardiac muscle cell; SMC, smooth muscle cell. B) Violin plots showing the normalized expression levels of marker genes for three major muscle cell types. C) Significantly enriched biological process terms of specific gene signatures in three major muscle cell types. Numbers between parentheses represent significance expressed as ‐log_10_ (adjusted *p*‐value). D) Transcription factors (TFs) with different activity scores among three major muscle cell types. E) Volcano plot displaying differentially expressed genes (DEGs) between type I and type II myonuclei. F) Four candidate TFs with distinct activity scores in type I, IIa/b, and IIx myonuclei. G) RNA velocity analysis on the UMAP embedding demonstrating state transition from satellite cells to myofiber in the skeletal muscle tissue. The arrows represent a flow derived from the ratio of unspliced to spliced transcripts, which in turn predicts dynamic changes in cell identity. Heatmap on the right demonstrates stereotyped changes in gene expression trajectory. H) Dot plot showing the expression levels of selected marker genes for each smooth cell cluster. I) Heatmap indicating the tissue preference of each cell population across different tissues revealed by *R*_o/e_ (ratio of observed cell number to expected cell number).

The muscular system is a complex collection of organs that allow movement through the contraction of muscle fibers and is also the main production target of the pig industry, with the aim to provide high‐quality protein in the form of meat. There are three distinct muscle types in the body, namely skeletal, cardiac, and smooth muscle, each with unique cellular morphologies and functions.^[^ ^40^ ^]^ We found that skeletal muscle cells and cardiomyocytes accounted for 66.43% and 25.09% of total cells in muscle and heart, respectively, while smooth muscle cells could be found in eight tissues with an average proportion of 2.52% (Figure S12–S14, Supporting Information). To provide a more detailed view of the three muscle cell types, we extracted a total of 10 117 muscle cells from corresponding tissues according to cell‐type annotations and performed the dimension reduction analysis. As expected, UMAP inspection and dendrogram showed a clear separation among the three major muscle cell types, and each specifically expressed its classical marker genes (**Figures** **2**A,B and S19, Supporting Information), such as *MYH7*, *MYBPC2*, and *TNNT1* for skeletal muscle cells, *MYBPC3* and *TNNT2* for cardiac muscle cells (CMCs), and *ACTA2*, *MYH11*, and *RYR2* for smooth muscle cells. We observed a preferential grouping of skeletal muscle cells with CMCs since both belong to striated muscle tissue and share similar structural and functional characteristics.^[^ ^41^ ^]^ In addition, skeletal and smooth muscle cells could be further partitioned into multiple subclusters in the hierarchy (Figure 2B), suggesting their subtle context‐dependent functions. To examine global transcriptional differences among the three muscle cell types, we performed a pair‐wise differential gene expression analysis. In total, we identified 1250 differentially expressed genes (DEGs) across the three myocyte subtypes (Figure 2C). The 343 DEGs in skeletal muscle cells were significantly enriched in striated muscle contraction, while DEGs in CMCs were mainly involved in cardiac muscle tissue development. Smooth muscle cell‐specific genes were enriched in the extracellular matrix organization (Figure 2C). Further analysis of transcription factor (TF) activity revealed many remarkable regulons in the control of muscle cell‐type specification (Figure 2D). For example, MYOD1, MYOG, and FOXO4 served as master TFs responsible for skeletal muscle cell development, while certain members of the GATA and TBX families showed unique regulatory roles in the cardiac and smooth muscle cell types, respectively.

In addition to characterizing differences across the three main muscle cell types, we further probed cellular heterogeneity within skeletal and smooth muscle cells separately. Our analysis of pig myonuclei in skeletal muscle confirmed the presence of *MYH7* type I (slow‐twitch) and *TNNC2* type II (fast‐twitch, IIa/b, and IIx) myofibers (Figure 2B), consistent with a previous study in monkeys.^[^ ^5^ ^]^ A pairwise comparison between type I and type II myofibers uncovered 209 DEGs (Figure 2E). Notably, type I myofiber‐specific genes were enriched in several fundamental pathways related to molecular structure and function such as muscle contraction and sarcomere organization (Figure S20A, Supporting Information), while the upregulated genes in type II myofibers were essential for metabolic pathways such as phosphorylation and glycolysis (Figure S20B, Supporting Information). By examining DEGs of these two types of myofibers previously reported in humans,^[^ ^42^, ^43^ ^]^ we observed a strong positive Pearson correlation of 0.945 regarding fold changes of the shared genes between pigs and humans (Figure S20C, Supporting Information), implying that the process of muscle fiber specialization might be highly conserved between these two species. Furthermore, we identified several critical master regulators, including METTL3, MYF6, and SIX4, which displayed distinct regulatory activities in type I, IIa/b, and IIx myonuclei (Figure 2F and Table S4, Supporting Information). By conducting RNA velocity analysis in myofibers together with satellite cells (known as skeletal muscle stem cells), we further explored the differentiation trajectory of muscle fibers. Our results revealed clear myogenesis from satellite cells to mature muscle fibers (Figure 2G), which were driven by several fundamental genes with dynamic expressions across distinct cell states such as *MYH7* and *PRKG1*. Interestingly, the type IIa/b fibers displayed intermediate cell states and characteristics during the fast‐to‐slow fiber‐type switch, which could be driven by several stimuli, including exercise, hormones, and nutritional status.^[^ ^44^, ^45^, ^46^ ^]^ Type II fibers are reported to contract more faster and produce more power, but for shorter durations, and fatigue more easily than type I fibers. Generally, domestic pigs were intensively raised at a small place in the modern industry and did not largely use type II fibers for explosive movements, potentially leading to the intermediate cell states of type II fibers and the transition to type I fibers. In the smooth muscle cell compartment, we found distinct gene signatures and tissue enrichment among these six cell subtypes (Figure 2H,I). For instance, SMC_1, which was preferably located in the intestine, showed much higher activity of *MYH11* and *DMD* and subtype‐specific GO terms related to aiding in sustaining contraction (Figure S20D, Supporting Information), such as protein localization to the endoplasmic reticulum and regulation of actin filament‐based process. While SMC_6, mainly from testis, exhibited exclusively high expression of *MYO1B* and *RGS5*, and these signature genes were enriched in muscle system process and structure organization terms. These results suggested that the same cell types undergo subtle processes of functional differentiation depending on the original tissue contexts in which they reside.

To investigate conserved genes and TFs in CMCs across species, we analyzed heart scRNA‐seq/snRNA‐seq data from pigs (this study and Wang et al.^[^ ^27^ ^]^), monkeys,^[^ ^5^ ^]^ and humans.^[^ ^2^ ^]^ Mouse^[^ ^1^ ^]^ heart data were excluded due to the inclusion of only one neonatal heart tissue sample and low number of cells. For cross‐species comparison, we identified 13 013 one‐to‐one orthologous genes among the three species using the Ensembl BioMart. The UMAP visualization illustrated the integrated cell types, and the violin plot displayed specific marker gene expression for each cell type (Figure S21A,B, Supporting Information). Subsequently, we extracted CMCs from the three species for further analysis, comprising 2200 human CMCs, 5238 monkey CMCs, and 4132 pig CMCs (Figure S21C, Supporting Information). The CMCs gene expression patterns were categorized into four modules: a conserved module was widely expressed across all three species, and three species‐specific modules (human, monkey, and pig). In the conserved module, several genes such as *ACTN2*, *TNNT2*, and *MYOZ2* consistently showed high‐level expression in CMCs among the three species (Figure S21D, Supporting Information). GO enrichment analysis indicated that the conserved module was significantly enriched in the muscle system and muscle contraction terms, reflecting the evolutionary conservation of CMCs function (Figure S21, Supporting InformationE). Furthermore, each species‐specific module demonstrated distinct functional enrichment patterns. Additionally, we analyzed the TFs activity in CMCs across the three species. Notably, the TFs MEF2A, TEAD1, PPARA, and GATA4 displayed species‐specific transcriptional activity scores, suggesting distinct regulatory mechanisms in CMCs (Figure S21F, Supporting Information). These TFs in CMCs regulate the expression of cardiac genes and play pivotal roles in transcriptional regulation during embryogenesis. For example, GATA4 is an important member of the GATA family and directly regulates the expression of cardiac‐specific genes.^[^ ^47^ ^]^ A previous study showed that disruption of GATA4 leads to embryonic lethality in mice. Moreover, the TFs of the MEF2 family were reported to participate in regulating gene expression during myocardial cell hypertrophy.^[^ ^48^ ^]^

### 2.3 About the Similarity and Heterogeneity of Intestinal Epithelial Cells

**Figure 3:**
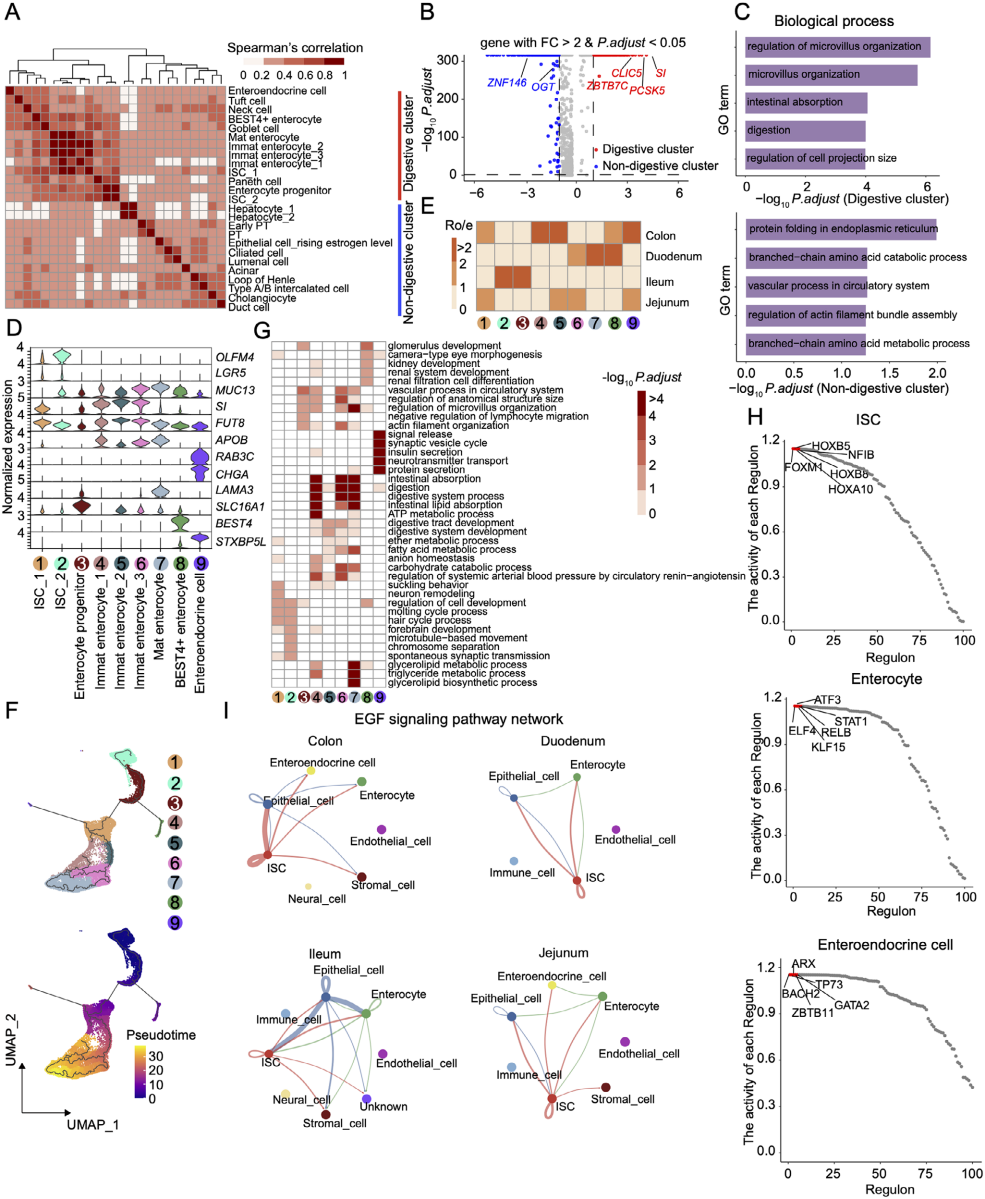
Shared and tissue‐specific molecular features for epithelial cell compartments. A) Heatmap showing Spearman correlation coefficient between 25 epithelial cell subtypes which could be broadly classified into digestive and non‐digestive groups. B) Volcano plot displaying differentially expressed genes (DEGs) between the digestive and non‐digestive clusters. Dots in the volcano plot highlight up‐regulated genes in each group. C) Functional annotation of up‐regulated genes in each group. Top enriched biological processes terms are listed. D) Violin plots showing the normalized expression levels of marker genes for each cell subtype. E) Heatmap indicating the tissue preference of each cell population across four intestinal segments revealed by *R*_o/e_ (ratio of observed cell number to expected cell number). F) Uniform Manifold Approximation and Projections (UMAPs) showing the pseudotime differentiation trajectories of intestinal stem cells, enterocytes, and enteroendocrine cells, respectively. G) Heatmap representing the enrichment of biological process terms in epithelial cell subtypes. H) Scatter plots showing the top 100 regulons of the three major epithelial cell subtypes. Each regulon is ordered by activity score, and the top five regulons with high activity are highlighted in red. I) The inferred EGF signaling pathway network among the major cell types in four intestinal segments. The edge width represents the communication probability, and a thicker edge line indicates a stronger signal.

Epithelia are sheets of cells that cover most body surfaces, line internal cavities, and compose certain glands. They perform a wide range of biological functions, including protection, absorption, and secretion.^[^ ^49^ ^]^ First, we pursued to investigate the primary characteristics and functions of epithelial cells, given their high abundance and diversity in the different organs. We obtained a total of 57 049 epithelial cells from eight tissues and identified their tissue‐specific expression patterns and functions through the global t‐SNE and hierarchical clustering (**Figures** **3**A, S22, Supporting Information). Epithelial cells from the duodenum, jejunum, ileum, and colon, representing the digestive system in the present study, exhibited closer relationships with other cells from the same digestive system than with cells from other systems. As expected, epithelial cells from the intestines showed a strong digestive and metabolic capacity, such as microvillus organization and intestinal absorption, compared to other subtypes (Figure 3B,C). We then extracted intestinal stem cells, enterocytes, and enteroendocrine cells for further exploration, as these cell types might play pivotal roles in feed efficiency traits in pigs.^[^ ^50^, ^51^, ^52^ ^]^ Intestinal stem cells expressed high levels of *OLFM4* and *LGR5* and could be further subdivided into two subtle subtypes according to the differential expression levels of these two markers (Figure 3D). We defined four enterocyte subgroups by the transcriptional patterns of canonical enterocyte markers (for example, *MUC13*, *SI*, *FUT8*, *APOB*, and *BEST4*), including enterocyte progenitors, immature enterocytes, mature enterocytes, and BEST4^+^ enterocytes. Enteroendocrine cells, which are specialized gut epithelial cells that produce and release hormones in the intestine,^[^ ^52^ ^]^ displayed a higher expression of *RAB3C*, *CHGA*, and *STXBP5L* when compared to other intestinal epithelial cells. Enrichment analyses of cell types across tissues revealed that intestinal stem cells were mainly located in the ileum and, to some extent, in the jejunum and colon, while enterocytes were more abundant in the duodenum and colon (Figure 3E).

To further characterize the lineage relationships and cell states among intestinal stem cells, enterocytes, and enteroendocrine cells, we conducted the pseudotime analysis and cell cycling index prediction.^[^ ^3^, ^53^ ^]^ Both analyses revealed that intestinal stem cells and enterocyte progenitors exhibited a great capacity for differentiation into enterocytes and enteroendocrine cells, as evidenced by their high proliferative states (Figure 3F and Figure S23A,B, Supporting Information). The differentiation trajectory of these intestinal epithelial cells was highly similar among the four individual intestine segments (Figure S23C–F, Supporting Information). Functional annotation analyses based on the Gene Ontology (GO) database demonstrated that gene signatures of each cell subgroup in intestinal stem cells were mainly enriched in cell cycle related biological processes as expected (Figure 3G), in line with their self‐renewal and differentiation ability.^[^ ^54^, ^55^, ^56^ ^]^ Notably, the two ISC subtypes showed slight differences in cell state and differentiation trajectories (Figures S23 and S24, Supporting Information), which might be partly attributed to the location‐specific contribution. The highly expressed genes in BEST4^+^ enterocytes were over‐represented in cell development and morphogenesis, which was highly different from the functions of immature and mature enterocytes. The gene sets restricted in enteroendocrine cells were significantly enriched in signal release and protein secretion (Figure 3G). The distinct transcriptional profiles and functions of these cell types can be attributed to their diverse gene regulatory programs (Figure S25, Supporting Information). By inferring the TF activity across the trajectory, we found that three master regulators, NFIB, STAT1, and ZBTB11, play essential roles in enterocyte lineage specification by a coordinated sequential activation (Figure 3H and Figure S26, Supporting Information). To compare the structures and intensities of cell–cell communication across the four gut segments, we employed CellChat^[^ ^57^ ^]^ to identify potential ligand‐receptor pairs among the major cell types. Our results revealed that EGF, PDGF, and BMP signaling pathways were major communicating pathways in the porcine intestine segments (Figure 3I, Figures S27, S28 and Table S5, Supporting Information). Although the global interaction patterns were similar, the strength of intercellular interactions was different across intestine segments. For instance, compared with the colon, we observed stronger intercellular interactions among enterocytes, epithelial cells, and intestinal stem cells in small intestine tissues. We further mapped ligand–receptor pairs in specified cell subpopulations across different organs to understand the rewiring of molecular interactions regulating cell–cell interactions. Notably, the “NAMPT‐INSR” and “GHRL‐GHSR” ligand‐receptor pairs were specific in interactions between enterocytes, suggesting the potential functions contributing to digestion, absorption, and metabolism in the intestine. For example, the “NAMPT‐INSR” pair regulates nutrient uptake and utilization by mediating insulin action on glucose and lipid metabolism.^[^ ^58^ ^]^ the “GHRL‐GHSR” pair plays a key role in the control of food intake and gastric emptying by regulating the secretion of ghrelin.^[^ ^59^, ^60^ ^]^ Overall, our findings highlight the importance of dynamic information exchange between different cells in contributing to the diverse digestive functions of different intestine sections.

### 2.4 A Cross‐Tissue Reference of Immune Cell Types and States

**Figure 4:**
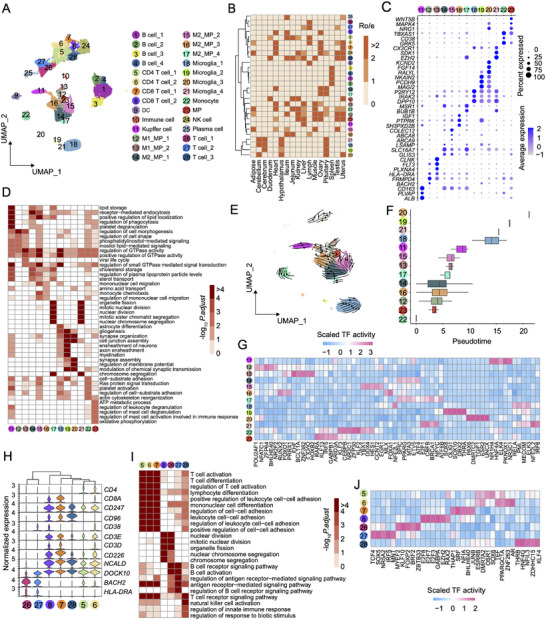
Immune cell heterogeneity across tissues in pigs. A) Uniform Manifold Approximation and Projection (UMAP) visualization of immune cell types across different tissues. Each dot represents one cell, with colors coded according to manually annotated cell types. B) Heatmap indicating the tissue preference of annotated immune cell types across different tissues revealed by *R*_o/e_ (ratio of observed cell number to expected cell number). C) Dot plot showing the expression levels of selected marker genes for each cell cluster. D) Heatmap representing the enrichment of biological process terms for monocyte and macrophage lineages residing in different tissues. E) UMAP showing the pseudotime differentiation trajectories of monocyte and macrophage lineages. F) Box plots denoting the distribution of estimated pesudotime value for each cell type by Monocle3. G) Heatmap showing transcription factors (TFs) with distinct activity scores in six major myeloid cell compartments. H) Violin plots showing the normalized expression levels of marker genes for T‐cell populations. I) Heatmap representing the enrichment of biological process terms for T‐cell subtypes in different tissues. J) Heatmap showing TFs with different activity scores among different T‐cell subtypes.

The immune system is a complex network of cell types distributed throughout the whole body and provides protection against bacteria, viruses, and other pathogens. Understanding the specific and shared features of tissue‐resident immune cells is crucial for deciphering the molecular mechanisms underlying immune responses and ultimately for accelerating precision breeding of disease resistance in pigs. We identified a total of 45 491 immune cells prevailing in 17 tissues, including T cells, B cells, natural killer cells (NK), macrophages, and other tissue‐resident immune cells (**Figures** **4**A and S29, Supporting Information). Hierarchical clustering analysis revealed three main branches of immune cells: myeloid and lymphoid lineages, as well as microglia, which are brain‐resident macrophages (Figure 4B). As expected, each tissue has its own immune compartments, with specific immune cell compositions. For example, the four major parts of the brain exclusively contain microglia cells, which are the first line of defense against pathogen invasion, brain damage, and cellular debris in the central nervous system.^[^ ^61^, ^62^ ^]^ The separation between microglia cells and macrophages in other tissues might be attributed to their difference in origin, morphology, marker genes, and cellular functions. A large population of B cells was evident in the spleen, whereas lymph nodes were enriched for multiple T‐cell types. We next subdivided and reanalyzed the immune dataset to explore further heterogeneity within macrophages and T cells, which were abundantly present across tissues. All tissue‐resident macrophages, together with monocytes, were divided into 13 more granular subsets, which were supported by the expression of well‐established marker genes (Figure 4C). These macrophage subgroups exhibited clear tissue‐type separation and preference, although certain subsets were shared by multiple tissues (Figure 4B). For instance, the M1 macrophage subgroups were enriched in muscle and liver, while M2 macrophages were mainly located in ileum and ovary. To further dissect cell‐type‐specific transcriptional profiling, we performed pair‐wise differential expression analyses and identified 2903 genes with restricted expression in one or a few cell types (Figure S30A, Supporting Information). Functional enrichment analysis evidenced the presence of overrepresented biological processes for each macrophage subtype, which recapitulated cell‐type‐specific functions regarding resident tissues and niches as well as putative cellular states (Figure 4D,F and Figure S30B, Supporting Information). Furthermore, cell‐type‐specific transcriptional programs were combinatorially controlled by several TFs with overlapping expression patterns. For example, the regulons KLF3 and CEBPB were exclusively expressed in monocyte subsets and showed a gradual decrease in expression levels across the monocyte‐to‐macrophage differentiation trajectory (Figure 4G). This finding indicated that these lineage‐restricted TFs were the key driving force behind the cell fate decision.^[^ ^63^, ^64^, ^65^ ^]^

T cells play a crucial role in elicitating and controlling the adaptive immune response.^[^ ^66^ ^]^ We identified seven T‐cell clusters based on known gene signatures, with CD4^+^ and CD8^+^ T cells showing a distinct separation, while the remaining clusters were designated as general T cells due to the absence of significant CD4 or CD8 surface molecules (Figure 4H). CD4^+^ and CD8^+^ T cells in our data were further divided into two subtle clusters, respectively, based on the transcriptional differences of several classical markers such as *CD3E* and *NCALD*. While these annotated T‐cell clusters were observed in 14 organs, their relative proportion and enrichment varied greatly across different organs (Figure 4B). CD4^+^ T cells were primarily located in lymph nodes and jejunum, whereas CD8^+^ T and NK cells were more abundant in heart and ovary. To understand their potential diverse biological functions, we identified DEGs among these T‐cell subtypes and then carried out a functional annotation. The majority of T cells shared several enriched GO terms, such as T‐cell activation and T‐cell receptor signaling pathway, suggesting their shared immune functions regardless of tissue origins. Specifically, signatures of CD4^+^ T cells were enriched for cell–cell adhesion, whereas CD8^+^ T cells had enhanced biological functions in nuclear division and regulation of antigen receptor‐mediated signaling pathway (Figure 4I and Figure S31, Supporting Information). The distinct transcriptional profiles and molecular functions were attributed mainly to the specific TF network (Figure 4J). Notably, the significant terms related to the cell‐cycle process suggested that the two T‐cell subtypes might undergo proliferative expansion into effector T cells in response to antigenic stimulus.^[^ ^67^, ^68^ ^]^ Overall, our study provides valuable insights into the diversity and complexity of T‐cell populations across different organs and sheds light on their roles in regulating the immune response.

### 2.5 Genetic Mapping and Functional Implications of Cell‐Type‐Specific eQTL

Bulk tissue samples often contain a high degree of cellular heterogeneity, which can mask genetic effects that are active only in specific cell types within the sampled tissue. To address this, we explored ieQTL by performing the cell‐type deconvolution analysis of 3921 bulk RNA‐seq samples in the PigGTEx project via this newly built cross‐tissue cell atlas. First, we tested the cell estimation performance of the CIBERSORT algorithm^[^ ^69^ ^]^ in pigs by deconvoluting pseudo‐bulk samples generated from simulation studies using the SCDC software.^[^ ^70^ ^]^ By employing the gene signature matrix built from our liver snRNA‐seq data, we observed that the estimated cell proportions from pseudo‐bulk samples were highly correlated with the putative cell populations identified in the liver snRNA‐seq, with the highest correlation in Hepatocyte_1 subtype (Pearson's *r* = 0.841, *p*‐value < 2.2  ×  10^−16^, Figure S32A–D, Supporting Information). This result indicated the feasibility and accuracy of our cellular deconvolution pipeline in pigs. To identify cell‐type‐specific eQTL in an unbiased manner, we performed eQTL deconvolution analysis by integrating our cross‐tissue snRNA‐seq data with the large‐scale bulk RNA‐seq collections from the PigGTEx project.

**Figure 5:**
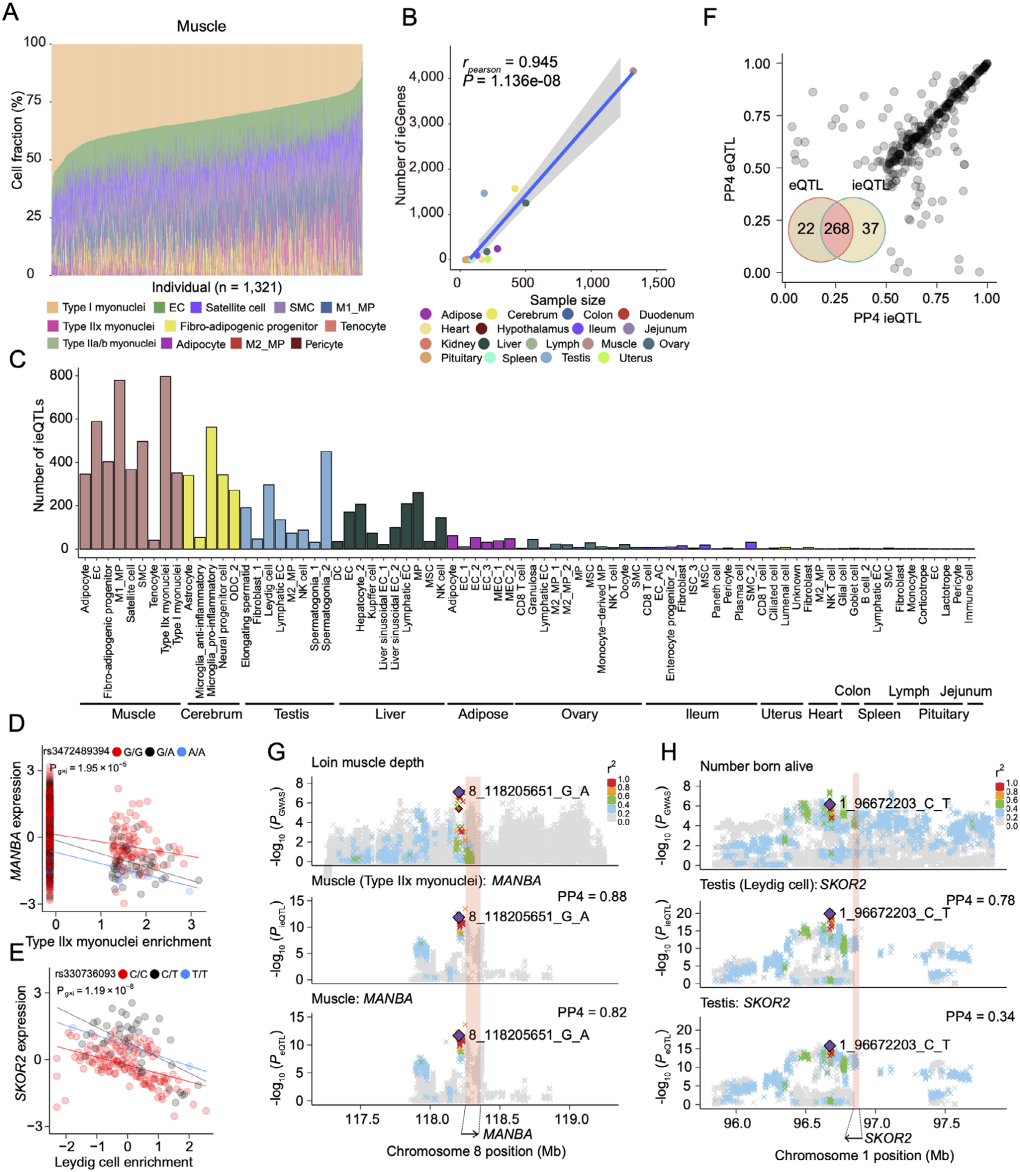
Cell‐type‐dependent activities of genetic variants on gene expression and pig traits. A) Stacked bar plots showing the fraction of cell types estimated in PigGTEx RNA‐seq samples based on our snRNA‐seq reference matrix in muscle tissue. B) Scatter plot showing the estimated number of ieGenes versus sample sizes for 17 tissues estimated using public bulk RNA‐seq datasets. C) Number of cell‐type interaction QTL (ieQTL) discovered in each cell‐type tissue combination at FDR < 5%. D) An ieQTL of *MANBA* showing cell‐type‐specific effects in type IIx myonuclei from muscle. Each point represents an individual and is colored by three genotypes. Both gene expression levels and cell‐type enrichment values are inverse normal transformed across samples. The lines are fitted by a linear regression model using the geom_smooth function from ggplot2 (v3.3.2) in R (v4.0.2). **E**) An ieQTL of *SKOR2* showing cell‐type‐specific effects in Leydig cell from testis. Each point represents an individual and is colored by three genotypes. Both gene expression levels and cell‐type enrichment values are inverse normal transformed across samples. The lines are fitted by a linear regression model using the geom_smooth function from ggplot2 (v3.3.2) in R (v4.0.2). F) Overlaps between ieQTL and eQTL detected by traditional bulk RNA‐seq. G) Aligned Manhattan plots of pig GWAS, ieQTL, and eQTL at the *MANBA* locus for loin muscle depth trait (LMDEP). SNPs are colored according to the magnitude of linkage disequilibrium (*r* ^2^) between adjacent SNP pairs. Orange shading indicates the gene position and the black arrow (bottom) indicates the gene expression direction. H) Aligned Manhattan plots of pig GWAS, ieQTL, and eQTL at the *SKOR2* locus for number born alive trait (NBA). SNPs are colored according to the magnitude of linkage disequilibrium (*r* ^2^) between adjacent SNP pairs. Orange shading indicates the gene position and the black arrow (bottom) indicates the gene expression direction.

The pseudo‐bulk gene expressions of our snRNA‐seq data were significantly correlated with those of PigGTEx bulk samples across all the 17 matching tissues, with correlation coefficients ranging from 0.498 (colon) to 0.745 (spleen), implying sufficient concordance for the subsequent integration (Figure S32E, Supporting Information). We thus estimated the relative cell fractions of these 17 PigGTEx tissues using the snRNA‐seq signature matrix of the respective tissues, where sample sizes of PigGTEx tissues varied from 44 (kidney) to 1321 (muscle). Overall, most samples were well‐deconvoluted (*p*‐value < 0.05, 1000‐times permutations) and revealed a striking variability in cellular composition across the PigGTEx samples (**Figures** **5**A and S33, Supporting Information). The number of putative cell types detected in deconvoluted samples ranged from six (uterus) to 23 (ileum) (Figure S32F, Supporting Information). In particular, the predicted abundance of cell types in muscle and heart displayed considerable inter‐individual variations, with certain cell types in some samples even being totally missing, while colon and hypothalamus showed less heterogeneous cell fractions across samples (Figure S33, Supporting Information). To map ieQTL, we performed a linear regression analysis that models an interaction term between estimated cell fractions and genotypes.^[^ ^71^ ^]^ We detected a total of 5168 protein‐coding genes with at least one significant ieQTL (ieGenes) across cell types and tissues (Figure 5B), with around a third of these ieQTL validated using the allele‐specific expression approach. Of note, muscle exhibited the highest number of significant ieGenes, followed by cerebrum, testis, liver, and adipose tissues (Figure 5C). The discovery power of ieGenes in tissue was significantly correlated with its sample size (Figure 5B). We detected an average of 114 ieGenes across 79 cell types from 14 tissues. Among them, type IIx myonuclei had the largest number of ieGenes (*n* = 797), whereas ileum Paneth cells only had one ieGene. For instance, the effects of rs3472489394 and rs330736093 on *MANBA* and *SKOR2* significantly interacted with the enrichment of type IIx myonuclei in muscle and Leydig cells in the testis, respectively (Figure 5D,E and Figure S34A, Supporting Information).

Furthermore, to explore the cellular effects of trait‐associated variants, we performed a colocalization analysis between ieQTL and GWAS hits of 268 complex traits in pigs (Table S6, Supporting Information). Of the putative ieQTL, 305 loci colocalized with at least one pig GWAS hit (Figure 5F), indicating a potential involvement in the genetic control of complex traits. By comparing GWAS colocalization results between standard PigGTEx eQTL and the newly detected ieQTL, we found that a substantial proportion of GWAS signals (> 81.96%) could be colocalized by both ieQTL and eQTL (Figure 5F–H, Figure S34B and Table S7, Supporting Information). For example, we found a promising colocalization between the *MANBA* gene in muscle and loin muscle depth (Figure 5G), which was supported by both ieQTL (posterior probability of colocalization (PP4) = 0.88) and standard eQTL (PP4 = 0.82). Of note, there were 37 ieQTL‐specific GWAS colocalizations (Table S7, Supporting Information), representing 19 complex traits, which indicated the cell‐specific regulation of these traits and their potential cellular origin. We also discovered that some GWAS hits missed by bulk eQTL could be retrieved by ieQTL. A noteworthy example was the Leydig cell ieQTL of *SKOR2* in testis (Figure 5H), which colocalized with the GWAS signal for the number of born alive at birth (PP4 = 0.78), whereas the standard eQTL from bulk testis tissues did not (PP4 = 0.34). Furthermore, ieQTL analysis indicated that *MANBA* expression in individuals carrying the A allele at rs3472489394 reduced with increased enrichment of Type IIx myonuclei (Figure 5D), and that rs330736093‐C decreased *SKOR2* expression with increased enrichment of Leydig cells, suggesting that the SNPs may affect gene expression through cell‐type‐specific regulations and interactions, further influencing cellular proportions and functions. These results together showcased the substantial potential of our cell atlas in dissecting the genetic control of the transcriptome and complex phenotypes at single‐cell resolution in pigs.

### 2.6 Association of Cell Types with Complex Traits and Diseases in Pigs and Humans

**Figure 6:**
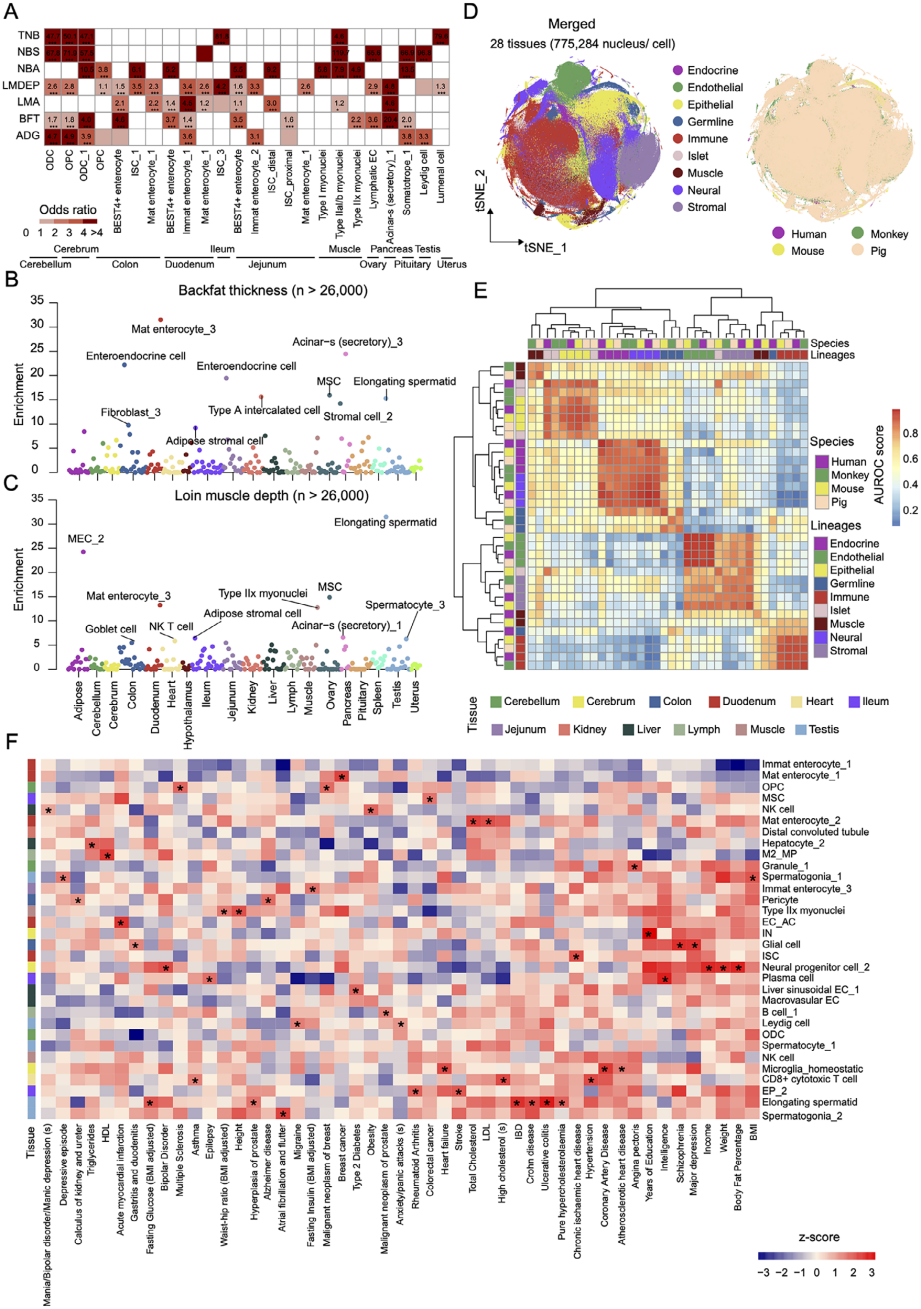
Association of single‐cell transcriptomic profiles with complex traits in pigs and humans. A) Heatmap showing representative significant associations between cell types and traits in pigs. Definitions for abbreviations and complete results are provided in Table S7 (Supporting Information). B) Evaluation of the enrichment of backfat thickness trait in putative cell types by scRNA‐seq data. Each circle represents a cell‐type‐trait association from a large‐scale population dataset. C) Evaluation of the enrichment of loin muscle depth trait in putative cell types by scRNA‐seq data. Each circle represents a cell‐type‐trait association from a large‐scale population dataset. D) t‐Distributed Stochastic Neighbor Embedding (t‐SNE) showing the distribution of nine major lineages from human, monkey, mouse, and pig. The single‐nucleus/cell RNA‐seq data from the four species was downloaded from public datasets and a total of 775 284 nuclei/cells were integrated by the Harmony algorithm after quality control. E) Pair‐wise correlation based on the expression levels of orthologous genes for the nine major lineages between human, monkey, mouse, and pig based on the area under the receiver operator characteristic curve (AUROC) scores. The AUROC scores were calculated by MetaNeighbor to measure the similarity of different lineages. F) Heatmap showing enrichment of pig cell types (indicated on the right) associated with selected human traits and diseases (indicated at the bottom). The colored boxes indicate selected enriched patterns. Definitions for abbreviations and complete results are provided in Tables S9 and S10 (Supporting Information).

Although ieQTL have provided new potential target genes and variants underlying GWAS loci, the causal cell types of complex phenotypes are yet to be fully understood. To systematically infer the relevance of cell types with complex traits and diseases, we conducted the GWAS signal enrichment analyses using the signature genes of each cell type. The complex traits collected in the PigGTEx project (Table S6, Supporting Information) were grouped into five main categories, including reproduction traits (*n* = 71), health traits (*n* = 61), meat and carcass traits (*n* = 50), production traits (*n* = 19), and exterior traits (*n* = 6). Of the 263 high‐resolution cell clusters in all 19 tissues, 222 (84.41%) showed significant enrichments for at least one phenotype category after multiple testing correction (Figure S35, Supporting Information). For instance, the litter size relevant traits were maximally enriched in the immune cell cluster, implying the existence of critical relationships between immune function and piglet survival (Figure S36, Supporting Information). Notably, many reproduction traits, such as total number born alive (NBA), total number of piglets born (TNB), and the number of stillborn pigs (NBS), showed a significant enrichments in neuronal cell types such as oligodendrocyte in cerebrum and cerebellum, in addition to Leydig cells in testis, endothelial cells in ovary and lumen cells in uterus (**Figures** **6**A and S37, Supporting Information). Moreover, several production and growth traits, including average daily gain, backfat thickness, and loin muscle area, were enriched not only in three skeletal myocytes but also in pituitary somatotropes, intestine enterocytes, and pancreatic acinar cells (Figure 6A). However, we did not find any significant enrichment for health and exterior traits, possibly due to their relatively low GWAS power. To validate the results, we partitioned the heritability of two production traits, backfat thickness, and loin muscle depth, by cell types in a large population of over 26 000 genotyped individuals (Figure 6B,C, Table S8, Supporting Information). As expected, we observed the enriched heritability of muscle depth trait in type IIx myonuclei. Likewise, backfat thickness showed a remarkable enrichment for enterocytes in the duodenum and enteroendocrine cells in the jejunum and colon. Although both results were obtained from two datasets with different sample sizes and distinct enrichment approaches, they showed to some extent consistency (Figure 6A–C). Furthermore, through examining the gene‐traits/disorders from Online Mendelian Inheritance in Animals database (OMIA, https://omia.org/), we identified notable cell‐type‐specific expression programs of many essential genes (Figure S38, Supporting Information). For example, *APOE* is associated with hyperlipdemia/atherosclerosis disease in OMIA. After 6 months of a high‐fat and high‐cholesterol diet, Fang et al.^[^ ^72^ ^]^ revealed that severe hypercholesterolemia and progressively formed human‐like atherosclerotic lesions in pigs. *APOE*, a major risk factor gene for Alzheimer's disease,^[^ ^73^ ^]^ showed higher transcription levels in the pig astrocyte and microglia subtypes compared to other cell types. High levels of *CD163* expression (an essential receptor for the porcine reproductive and respiratory syndrome^[^ ^74^ ^]^) were mainly observed in the Kupffer cells and other macrophages.

Given similar expression patterns in several cell types between pigs and humans, we further extended the cross‐species comparison analysis. We collectively analyzed scRNA‐seq data from human,^[^ ^2^ ^]^ monkey,^[^ ^5^ ^]^ mouse,^[^ ^1^ ^]^ and pig (Wang et al.^[^ ^27^ ^]^ and our dataset) using one‐to‐one orthologous genes, and compared the cellular compositions and transcriptomic landscapes of the cell reference map across the four species. After quality control, a total of 775 284 eligible cells were included in the integrated cell atlas (Figure 6D and Figure S39, Supporting Information), in which we defined 88 cell types and categorized these into nine major cell lineages according to our previous method. We found great consistency in tissue distribution and cellular components across four species. To quantify the interspecies transcriptional similarity for the nine lineages, we performed pair‐wise correlation analysis using the area under the receiver operator characteristic curve (AUROC) scores calculated by the MetaNeighbor framework.^[^ ^75^ ^]^ Overall, the same lineage across the four species showed significantly higher similarity (Figure 6E). To refine the cross‐species comparisons, we further characterized the conservation and divergence of cell types within the five matched tissues, including kidney, liver, spleen, pancreas, and uterus. In line with previous studies,^[^ ^1^, ^2^, ^5^, ^27^ ^]^ each tissue displayed similar cellular landscape and transcriptional signatures across the four species (Figures S40–S44, Supporting Information), despite the presence of some differences in cellular proportions, which might be related to the difference in library preparation protocols (snRNA‐seq and scRNA‐seq from 10 × and microwell technologies) and sampling regions or due to true species‐specific features. These findings suggested significant conservation and divergence in cellular transcriptional programs during animal evolution and would pave the way for further biomodel and xenotransplantation research.

To explore whether our pig cell atlas could help to understand the cellular mechanisms of complex traits and diseases in humans, we quantified the heritability enrichment of 137 human complex phenotypes (Table S9, Supporting Information) across the 261 annotated cell types (two cell clusters defined as unknown types were discarded) via the stratified linkage disequilibrium score regression analysis (LDSC). We retrieved 15 354 one‐to‐one pig‐human orthologous protein‐coding genes from the Ensembl dataset (v104) using the BioMart tool^[^ ^76^ ^]^ for the following analyses. Our results revealed a total of 1547 significant associations (the corrected enrichment *p*‐value < 0.05) between pig cell types and human complex phenotypes (Figure 6F, Figure S45 and Table S10, Supporting Information). As expected, we observed significant enrichments of several neurological and psychiatric phenotypes, such as multiple sclerosis, schizophrenia, and bipolar disorder, in neural cell types, including excitatory neurons and neural progenitor cells from the cerebrum, as well as in certain immune cell clusters such as microglia from cerebrum and macrophages from pituitary. Additionally, metabolic traits, including type 2 diabetes and cholesterol‐related phenotypes, showed expected associations with hepatocytes, pancreatic duct cells, and ileum goblet cells, as well as interesting associations with several skeletal muscle and intestine cell populations. Moreover, our analysis revealed some novel relationships between GWAS traits and cell types. For instance, we found enriched heritability of several intestine diseases, such as Crohn's disease and diverticular disease, in cell clusters corresponding to brain‐resident immune cells,^[^ ^5^, ^15^ ^]^ in addition to enterocytes and immune cells from the four intestine segments. For fasting insulin and glucose traits, we found significant enrichments in adipocytes from adipose and skeletal muscle cells and enterocytes from the intestines. Similarly, we observed striking enrichments of anthropometric traits, including height, waist‐hip ratio, and body fat percentage, not only in intestinal stem cells, fibro‐adipogenic progenitor cells from skeletal muscle, and adipocyte from adipose but also in multiple cell populations from testis and ovary. Overall, our pig snRNA‐seq data provided new comprehensive insights into trait‐relevant cell types in both pigs and humans, which will boost the unravelling of molecular and cellular mechanisms underlying complex phenotypes and the potential utilization of pigs as human biomedical models for certain diseases.

## 3 Discussion

The domestic pig (*Sus scrofa*) is a valuable livestock species that contributes significantly to both agricultural and biomedical research. Recent studies, including our PigGTEx project, have revealed that many traits‐associated variants are located in non‐coding regions and affect the spatiotemporal expression of candidate genes in a context‐specific (tissue‐ or cell‐type‐specific) fashion. However, the impacts of genetic variations on these regulatory pathways and how they vary across trait‐relevant cell types have not been explored in pigs. To bridge the gaps between genetic variants and phenotypes at single‐cell resolution, we performed a comprehensive analysis by integrating a cross‐tissue snRNA‐seq atlas with the large‐scale PigGTEx datasets. This work not only establishes a comprehensive single‐cell reference map as a baseline for dissecting cellular heterogeneity within and across tissues but also highlights a more powerful strategy for identifying trait‐critical cellular signatures and cell‐type‐specific eQTL in pigs.

The present study employed single‐nucleus RNA‐seq to profile gene expression in 229 268 high‐quality cells from 19 tissues in pigs, similar to a recent study^[^ ^27^ ^]^ which constructed the first single‐cell transcriptomic atlas of 222 526 cells across 20 swine tissues. Compared with that work, our dataset uses a different pig breed (a Chinese native breed versus a three‐way hybrid), and represents a broader range of pig organ sources covering nine major body systems and especially comprises several highly important tissues in pig production performance, such as skeletal muscle, four intestine segments, and three reproductive organs. Given the large diversity in the chosen tissues, the two studies demonstrate a good complement and represent very significant contributions to the efforts of the pig single‐cell consortium. Notably, the two studies in pigs focused on addressing different biological questions by using the single‐cell transcriptomic atlas. Wang et al. showed basic biological knowledge of distinct cell types and regulons,^[^ ^27^ ^]^ and then underlined the cellular heterogeneity and function of endothelial cells in pigs due to their importance in human biomedicine research. However, in this study, we highlighted the importance of single cell atlas in understanding the genetic and molecular mechanisms underlying bulk eQTL and complex traits through identifying trait‐critical cellular signatures and cell‐type‐specific eQTL, besides providing the detailed characterization of cellular similarity and heterogeneity across tissues associated with economic traits. Furthermore, this work was the first study integrating single‐cell data and population genetics in farm animals as far as we know.

In line with single‐cell landscapes in other species,^[^ ^1^, ^2^, ^4^, ^5^, ^77^, ^78^, ^79^ ^]^ we identified primary cell classes based on known canonical marker genes and captured a few rare cell types such as Purkinje cells from the brain and enteroendocrine cells from the intestine, which may facilitate our understanding of cell lineage trajectory and tissue homeostasis. Although the sample size in this study is limited, the identified cell types and marker genes demonstrate high consistency across multiple developmental stages, breeds, and even mammalian species, which strongly supports the reliability of our findings. Our pig cross‐tissue cell atlases clarify the heterogeneous characteristics in cellular compositions and molecular properties within and across tissues. For example, we delineated the global transcriptional divergence and transition pattern among three dominant myofiber types (type I, IIa/b, and IIx) and revealed evolutionarily conserved similarity in pivotal genes specializing myofiber, such as *MYH7* and *MYBPC2* across mammals.^[^ ^38^, ^42^, ^43^ ^]^ This finding may have important implications for improving meat quality and quantity, which are largely affected by myofiber characteristics and proportions in pigs.^[^ ^80^, ^81^ ^]^ Type II myonuclei exhibited a notable enrichment in metabolic processes, indicating their crucial involvement in metabolic traits and syndromes, i.e., meat production and fat deposition in pigs and type 2 diabetes and obesity in humans.^[^ ^42^, ^82^, ^83^, ^84^ ^]^ Notably, we did not detect the expression of the *MYH1* and *MYH4* genes based on the Ensembl annotation file (v104), which are three classical markers for skeletal muscle cells, and the discrepancy might be attributed to the incomplete genome assembly and annotations in pigs. Future studies based on the more complete reference genome and annotation files from multiple long‐read technologies would improve the results. Our data also demonstrate the prevalence of epithelial and immune cells across different tissue contexts and offer a more detailed understanding of cell compartments. Although some cells of a common type are shared across tissues, subpopulations are specifically enriched in particular tissues. These tissue‐resident epithelial and immune subsets are specialized to fulfill the specific functional demands of different tissues, probably owing to unique local environments or niches.^[^ ^78^, ^85^ ^]^

Although our PigGTEx project has provided a compendium of genetic regulatory effects across pig tissues and functional variants underlying complex traits,^[^ ^24^ ^]^ a comprehensive understanding of gene regulation at the single‐cell resolution for most noncoding loci is still lacking. To address this issue, emerging approaches such as single‐cell eQTL and heritability enrichment analyses have been extensively used in deciphering complex human traits and diseases^[^ ^14^, ^15^, ^16^, ^17^, ^71^ ^]^ but have yet to be systematically applied in pig studies. As a critical component of the PigGTEx project, our work offers an in‐depth dissection of the genetic effects of trait‐critical cellular signatures and cell‐type‐specific eQTL, in addition to the comprehensive pig cell reference map, setting it apart from other recent single‐cell studies.^[^ ^27^ ^]^ We revealed that around 15% of the loci that co‐localized with GWAS traits showed significant cell‐type specificity, underscoring the advantages of single‐cell eQTL analysis over the standard bulk eQTL approach. The proportion missed by bulk studies is slightly lower than what has been described in humans,^[^ ^71^ ^]^ which might be attributed to the limited sample size in our work. By linking individual cell types to complex traits, we identified substantial cell‐type‐trait associations that are consistent with previous studies,^[^ ^5^, ^15^, ^16^, ^42^ ^]^ suggesting high functional conservation of major cell types among mammal species.^[^ ^38^ ^]^ Furthermore, we mapped several unique associations between cell types and important phenotypes in pigs, such as the driving roles of myofiber cell types for meat production traits and Leydig cells from the testis for reproduction traits. Overall, our results provide meaningful insights into previously cryptic molecular and cellular mechanisms behind traits of economic importance and offer new opportunities for precision breeding in pigs.

Despite the significant findings of our study, several limitations must be noted. Firstly, the current dataset comprises only one male and one female pig and is not an exhaustive characterization of all pig organs. As such, we cannot fully capture the complete single‐cell picture and inter‐individual variation in cellular composition, potentially limiting our ability to explore rare cell types and map entire trait‐associated cellular signatures. Nevertheless, we have strengthened the reliability of our findings through the integration of public datasets, multi‐species comparisons, and additional single‐cell RNA‐seq data generated from liver tissue. Future studies with larger sample sizes will be valuable to validate and extend these findings. Secondly, compared with single‐cell RNA‐seq, our single‐nucleus RNA‐seq can only profile nuclear transcripts and not cytoplasmic transcripts. Different library preparation protocols may result in a reasonable proportion difference in specific cell types, such as muscle, neural, and immune cells, despite globally consistent detection performance in gene number and cell type diversity between them.^[^ ^86^, ^87^ ^]^ Thirdly, the sample size of certain tissues used in cellular deconvolution and heritability partitioning analyses is relatively small, limiting the statistical power to detect causative trait‐associated cell types and single‐cell eQTL. The prioritized variants, genes and cell types in this study cannot be directly explained as causal factors of complex traits, despite that we performed strict analyses and filtering. Further validation experiments such as single‐cell perturbation assays and in vivo models with targeted mutations at ieQTLs are required to confirm how genetic variations drive cell‐type‐specific gene expression and impact the phenotypic traits. Lastly, although the present work, similar to large‐scale multi‐omics studies such as the ENCODE,^[^ ^88^ ^]^ Roadmap^[^ ^89^ ^]^ and GTEx^[^ ^6^ ^]^ projects in humans, pinpoints many promising and testable biological hypothesis on trait‐associated determinants (variants and cell types) in pigs, it is worthwhile to remark that more in vitro and in vivo experiments are still needed. Therefore, future studies that incorporate larger sample sizes, different breeds, a broader range of tissues with multiple developmental stages, and several complementary single‐cell approaches (such as, scATAC‐seq and spatial transcriptomics), as well as solid validation experiments, will be required to establish a more comprehensive review of cellular reference maps in pigs and further provide more robust evidence supporting our findings.

## 4 Conclusion

This study presents a compendium of high‐resolution body‐wide single‐cell transcriptional landscape, provides a deeper understanding of the expression patterns and functions of tissue‐specific and shared cell types, and illuminates the intricate cell–cell interactions governing tissue homeostasis. Through pioneering single‐cell eQTL and colocalization analyses in pigs, we pinpointed the likely causative cell‐type‐associated variants and genes underlying traits of economic importance. Additionally, thousands of cell‐type‐trait associations were discovered, and previously unexplored biological mechanisms were explicated using heritability enrichment analysis. Collectively, these findings will significantly enhance our understanding of cross‐tissue and cross‐individual variations of cellular phenotypes and highlight promising trait‐associated determinants (variants and cell types) for advancing the fields of future pig breeding and human biomedical research.

## 5 Experimental Section

### Ethics Statement

All animal protocols and procedures were implemented in compliance with the Guide for the Care and Use of Experimental Animals established by the Ministry of Agriculture and Rural Affairs (Beijing, China) and were approved by the Institutional Animal Care and Use Committee of the Chinese Academy of Agricultural Sciences. Prior to tissue sampling, the pigs were humanely euthanized as necessary to minimize their suffering.

### Animals

Two Meishan pigs (one female and one male), aged 180 days, were obtained from a pig company managed under the same conditions (Nantong, Jiangsu). Eighteen tissues, including adipose, cerebellum, cerebrum, colon, duodenum, heart, hypothalamus, ileum, jejunum, kidney, liver, lymph, muscle, ovary, pancreas, pituitary, spleen, and uterus, were freshly harvested from postmortem female pig. The testis tissue was collected from the male pig, to chart the single‐cell view of the male primary reproductive organ. Each tissue was kept on ice and minced into 5–10 pieces weighing approximately 50–100 mg each on ice with sterilized scissors. Tissue samples were then snap‐frozen in liquid nitrogen and stored at −80 °C until nuclear extraction was performed.

### Cell Isolation

Single‐nucleus isolation and purification were conducted following the standard 10 × Genomics protocol (CG000375 • Rev B), with some minor modifications.^[^ ^28^, ^86^ ^]^ Briefly, the frozen tissue samples were homogenized using the Dounce homogenizer with 25 strokes of the loose pestle A and then 25 strokes of the tight pestle B in 1 mL of ice‐cold homogenization buffer (10 × 10^−3^ m Tris‐HCl pH 7.4, 10 × 10^−3^ m NaCl, 3 × 10^−3^ m MgCl_2_, 0.1% NP‐40, 1 × 10^−3^ m DTT, and 1.0 U µL^−1^ RNase inhibitor). Tissues were dounced until no visible cell clumps remained. Homogenates were incubated on ice for 5 min and pipetted a few times with a wide‐bore pipette tip during incubation. After this, the mixture was filtered through a 40‐µm cell strainer into a 1.5‐mL tube. To collect dissociated single nuclei, the sample was centrifuged at 500 g for 5 min at 4 °C, and the supernatant was discarded by pipetting. After centrifugation, the nuclear pellet was washed and resuspended in 1× PBS with 1.0% BSA and 1.0 U µL^−1^ RNase inhibitor, and centrifuged at 500 g for 5 min at 4 °C to discard cellular impurities within the supernatant. This step was repeated at least two times. Then the nuclei suspension was stained with a 7‐AAD ready‐made solution for flow‐cytometry‐based sorting of high‐quality nuclei, followed by a 5‐min incubation on ice. The pellet was resuspended in 250 µL of nuclei wash buffer (10 × 10^−3^ m Tris‐HCl pH 7.4, 10 × 10^−3^ m NaCl, 3 × 10^−3^ m MgCl_2_, 1.0% BSA, 0.1% Tween‐20, 1 × 10^−3^ m DTT, and 1.0 U µL^−1^ RNase inhibitor). Quality control of post‐sort nuclei concentration was evaluated under a microscope before loading into a 10× Chromium Single Cell Chip (10× Genomics, Pleasanton, CA) with a targeted capture of 8000 nuclei per channel.

### Single‐Nucleus RNA‐seq Library Preparation and Sequencing

The single‐nucleus RNA‐seq libraries were prepared following the standard protocol supplied by 10× Genomics (10× Genomics, Pleasanton, CA). In brief, isolated nuclei were captured in droplets with gel beads in the Chromium Controller. Following the RNA reverse transcription step, emulsions were broken, and barcoded cDNA was purified with Dynabeads, after which PCR amplification was performed. The amplified cDNA was then used for 3′ gene expression library construction. Then, indexed libraries were constructed according to the manufacturer's recommendations. After quality control, eligible libraries were sequenced on the Novaseq 6000 platform (Illumina) in a 150 bp paired‐end manner. The first 28 bp in read 1 captured both the 16 bp 10× barcode and the 12 bp UMI. All snRNA‐seq experiments were conducted following the standard protocols and were finished in a week by the same technician, to attenuate the batch effects possibly introduced by technical variations such as different operating peoples and processing time.

### Preprocessing of snRNA‐seq Data

The Sscrofa11.1 reference assembly^[^ ^90^ ^]^ in FASTA format and annotated gene model in GTF format were downloaded from the Ensembl database (https://ftp.ensembl.org/pub/release‐104/). Raw snRNA‐seq data were aligned to the pig reference genome and subjected to barcode assignment and UMI counting using the commands recommended by the CellRanger pipeline (10× Genomics, CA, USA). Given that snRNA‐seq captures both unspliced pre‐mRNA and mature mRNA, the include‐introns option was used for counting exonic and intronic reads together. The filtered gene expression matrix was used for further analysis with the Seurat package.^[^ ^91^ ^]^ To ensure the accuracy and robustness of the results, ambient RNA and potential doublets were removed using DecontX^[^ ^92^ ^]^ and DoubletFinder^[^ ^93^ ^]^ with default settings. This work also filtered out low‐quality nuclei expressing less than 200 genes or more than 5000 genes, and less than 500 UMIs or more than 15 000 UMIs, as well as those exceeding 5% of mitochondrial content. In general, the 10 × single‐nuclei data still contains a small amount of mitochondrial RNA since these transcripts might remain stuck to nuclear membranes or may get partitioned into GEMs which were attributed to the imperfect efficacy of the stripping process. High‐quality nuclei were kept with less than 5% mitochondrial genes for the subsequent analysis. During the gene filter step, all genes not expressed in at least three nuclei were removed. In addition, to balance the dataset in subsequent analyses, this work randomly selected 20 000 nuclei from the spleen, as it had a much higher number (*n* = 53 444) of captured nuclei compared to other tissues.

### Cell Clustering and Cell‐Type Annotation

After filtering, the remaining high‐quality data were log‐normalized and scaled to account for cell‐to‐cell variation with regression on the number of UMIs and percentage of mitochondrial genes. Subsequently, PCA linear dimensionality reduction analysis was performed, followed by t‐SNE and UMAP visualization approaches using the Scanorama tool,^[^ ^34^ ^]^ to capture the global cell‐type landscapes across tissues. For individual clustering, each tissue dataset was visualized using the Seurat package.^[^ ^91^ ^]^ Parameters used in each function were manually curated to obtain the optimal clustering of cells by adjusting the number of principal components and resolutions on a per‐dataset basis. The FindAllMarkers or FindMarkers function with default parameters were employed to identify marker genes of each cluster and annotated each cell type based on known classical markers from extensive published literature. The Pearson or Spearman correlation coefficients among cell types were calculated using the average expression of the top 1000 highly variable features, and the pheatmap package (https://github.com/raivokolde/pheatmap) was used to visualize the results. Considering that smooth muscle, epithelial, and immune cells are generally abundant across the body, the corresponding cell types were extracted and reanalyzed in each tissue to systematically resolve the fine‐grained variations in these cell types across tissues. The bioinformatics analyses, including dimensionality reduction, cellular annotation, trajectory inference, regulon prediction, and functional enrichment, were conducted as similar to the procedure in the total dataset. Besides, the expression of marker genes in different cell types was visualized with the ggplot2 R package.

### Assessment of snRNA‐seq Data Integration

The integration of snRNA‐seq data across 19 tissues using the “scib” package (https://github.com/theislab/scib‐pipeline)^[^ ^33^ ^]^ with default parameters was evaluated. Four frequently used single‐cell data integration tools were selected, including Scanorama,^[^ ^34^ ^]^ principal component analysis (PCA),^[^ ^35^ ^]^ ComBat,^[^ ^36^ ^]^ and Harmony^[^ ^37^ ^]^ for assessment of snRNA data integration. The accuracy of single‐cell data integration was assessed using two key metrics: batch effect removal and conservation of biological variance. These metrics would give an overall score by computing a 40:60 weighted mean of these two category scores.^[^ ^33^ ^]^

### Cell‐Type Diversity Estimation

Shannon entropy was calculated to evaluate cell type diversity in each tissue with a previously published method^[^ ^14^ ^]^ according to the formula $-\sum_{x}\left(p_{x}\times log_{2}\left(p_{x}\right)\right)$, where *p_x_* is the proportion of each cell type *x* in a tissue. The entropy value per tissue was plotted using the ggplot2 R package.

### Pseudotime Trajectory Inference and RNA Velocity Analysis

The cell lineage trajectory was inferred using Monocle 3^[^ ^53^ ^]^ according to the standard tutorial. The built‐in DDRTree algorithm was used for dimensional reduction and visualization after constructing the cell trajectory. Notably, the root state of the inferred trajectory was specified based on existing biological knowledge. Furthermore, the velocity streams and latent time assignments were predicted from sorted bam files using the dynamical model implemented in scVelo.^[^ ^94^ ^]^

### Cell‐Cycle Index Estimation

To further infer dynamic information about cell state, a cell‐cycle index for each cell type was calculated with a previously published method.^[^ ^3^ ^]^ Typically, progenitor cells with rapidly dividing capacity display higher cycling indices, whereas cell types that are known to be largely quiescent exhibit lower values. This work based on high‐confidence list of the cell‐cycle markers,^[^ ^3^ ^]^ and converted pig‐human orthologous genes (one‐to‐one) using the Ensembl BioMart. This list was provided Table S3 (Supporting Information). G0 cell‐cycle markers were defined as noncycling, while cell‐cycle markers of other phases (G1, S, G2, and M) were regarded as cycling. The cell‐cycle index is calculated using a formula:

(1) $$\mathrm{cell}\;\mathrm{cycle}\;\mathrm{index}=\mathrm{lo}g_{10}\frac{\mathrm{cycling}\;}{\mathrm{non}-\mathrm{cycling}\;}$$

The cell‐cycle index was calculated as the log10 ratio of the number of cycling cells to noncycling cells for each cell type using scanpy's score_gene function in the scanpy package.^[^ ^95^ ^]^

### Cell–Cell Interaction Analysis

To investigate cellular communication patterns between different cell types, the CellChat package^[^ ^57^ ^]^ with default parameters was used, which is a manually curated database of literature‐supported ligand–receptor interactions in humans and mice. To run CellChat analysis in pig datasets, pig genes were mapped to human orthologs according to the Ensembl annotation (v104). The CellChat quantifies the relative interaction strength between two cell groups by a probability value, which is modeled by the law of mass action depending on the average expression values of a ligand by one cell group and that of a corresponding receptor by another cell group, as well as their cofactors. The statistically significant intercellular communications were identified using a permutation test (*n* = 100 by default) by randomly permuting the group labels of cells, and then recalculating the communication probability. The interactions with *p*‐value < 0.05 were then considered to be significant.

### Tissue Enrichment of Clusters

The enrichment of each cell cluster across tissues was estimated, as previously described.^[^ ^96^ ^]^ In brief, the observed and expected cell numbers were calculated in each cell cluster to compute the ratio (*R*_o/e_) between the two values using the epitools R package. A cluster was considered to be enriched in a specific tissue if *R*_o/e_ > 1.

### Gene Ontology (GO) Enrichment Analysis

Gene ontology (GO) analysis was performed using the clusterProfiler 4.0^[^ ^97^ ^]^ and org.Hs.eg.db annotation package, considering that genome‐wide annotation is incomplete in pigs. The Benjamini–Hochberg (BH) procedure was used for the multiple testing corrections, and only GO terms with an adjusted *p*‐value smaller than 0.05 were retained.

### Single‐Cell Regulatory Network Analysis

To uncover cell‐type‐specific transcription regulons and construct gene regulation networks (GRNs), single‐cell regulatory network inference and clustering analysis were conducted using the SCENIC suite^[^ ^98^ ^]^ with the default parameters. The original expression matrix was normalized with Seurat and fed into SCENIC to build a coexpression network using the built‐in GRNBoost2 algorithm. The activity of regulons in each cell was calculated by the AUCell algorithm.

### Cellular Deconvolution Analysis Using CIBERSORT

For each tissue, DEGs specific to each cell type were first identified using the *Findmarkers* function in the Seurat package. The top 50 genes were then selected with the most significant overexpression, based on adjusted *p*‐value (<0.05) and average log_2_ fold change (>0.5), to build the gene expression signature matrix for the cell‐type reference set. To predict the abundances of cell types in a mixed cell population for each tissue, the RNA‐seq expression matrix of 17 matching bulk tissues were collected with snRNA‐seq data from the PigGTEx database (http://piggtex.farmgtex.org/). Subsequently, the CIBERSORT tool^[^ ^69^ ^]^ was selected for cellular deconvolution analysis, given its great resolving power.^[^ ^99^ ^]^ In addition, CIBERSORT could be easily downloaded for local use with the R code and is convenient to test multiple parameters such as data transformation, normalization method, and signature matrix, which was more easily controlled and flexible for large‐scale bulk RNA‐seq data. To test the robustness of cellular deconvolution, the *generateBulk_norep* function was first used in the SCDC package^[^ ^70^ ^]^ to obtain the transcript per million (TPM) matrix of 1000 pseudo bulk samples (default parameters) with known cell‐type distribution based on the liver snRNA‐seq data. Then CIBERSORT was used to perform deconvolution on these samples using the TPM matrix of signature genes from each cell type in pig liver. The number of permutation tests was set to 1000 times to determine the significance level, and *p* < 0.05 was regarded as statistical significance. Finally, the Spearman correlation coefficient was calculated between the known and predicted cell‐type distribution of hepatocyte cells to assess the accuracy of CIBERSORT deconvolution in pig dataset.

### Cell‐Type Interaction cis‐eQTL Mapping

To detect whether a *cis*‐eQTL explicitly affects gene expression in a given cell type, cell‐type interaction *cis*‐eQTL (ieQTL) mapping for 17 bulk tissues of PigGTEx was performed. The cell‐type composition (i.e., enrichment score) estimated from CIBERSORTx was used as above and only considered cell types with an enrichment score > 0 in at least 20 samples and/or 20% of samples within a tissue. For each tissue‐cell type pair, ieQTL mapping was performed via a linear regression model implemented in TensorQTL (v1.0.3),^[^ ^100^ ^]^ which included an interaction term between genotype and cell‐type enrichment score:

(2) y∼g+b+g×b+A

where *y* is the vector of gene expression value (i.e., the inverse normal transformed trimmed mean of M‐values, TMM), *g* is the genotype dosage (i.e., 0/1/2) vector of the tested SNP from PigGTEx samples, *b* is the enrichment score of a given cell type predicted from snRNA‐seq data, *g* × *b* is the interaction term between genotype and enrichment score, and A represents the covariates (i.e., genotype PCs and PEER factors, detailed in PigGTEx pilot phase). For the ieQTL mapping, SNPs within ±1 Mb of transcription start sites (TSS) of each gene were only considered. This work eliminated those SNPs with minor allele frequency (MAF) < 0.05 in the top and/or bottom 50% of samples sorted by the enrichment score of each cell type, using TensorQTL (v1.0.3) with parameter: –maf_threshold_interaction 0.05. To correct for the multiple testing at the gene level, eigenMT^[^ ^101^ ^]^ in TensorQTL was used for calculating the top nominal *p*‐value of each gene. This work then computed the genome‐wide significance of genes using the Benjamini–Hochberg FDR correction on the eigenMT‐corrected *p*‐values and defined as ieGene that with at least one significant ieQTL (i.e., FDR‐corrected *p*‐value < 0.05).

### Allele‐Specific Expression Validation of ieQTL

Allele‐specific expression (ASE) data were used at the individual level to validate the discovered ieQTL. This work first estimated the effect size (i.e., allelic fold change, aFC) of the top ieQTL for each ieGene from ASE data using the script phaser_cis_var.py in phASER (v1.1.1)^[^ ^102^ ^]^ and considered only ieQTL with nominally significant ASE (*p*‐value < 0.05) data in more than 10 heterozygous individuals with more than eight reads for a gene. To filter out outlier samples in ASE data, the median absolute deviation (MAD) was applied based on Hampel's test to the allelic imbalance (AI) ratio values ($|\frac{\mathrm{Reference}\;\mathrm{reads}}{\mathrm{Total}\;\mathrm{reads}}-0.5|$) across samples,^[^ ^71^, ^103^ ^]^ since MAD provides a more robust and flexible approach for outlier detection, particularly when dealing with non‐normal data, outliers, and heteroscedasticity,^[^ ^104^ ^]^ compared to the SD. When a sample had |AI_i_ − median (AI)|  ≥  4.5  ×  MAD, where MAD  =  median(|AI_i_ − median(AI)|) and AI_*i*_ is the allelic imbalance ratio value for the ith individual, this work defined it as an outlier. Subsequently, these outliers were eliminated in the validation process since these outliers exhibited either high (close to 1) or low (close to 0) frequencies of reference alleles and might negatively bias the results. Within a given tissue, this work determined that an ieQTL was validated by ASE data if it presented a nominally significant (*p*‐value < 0.05) Pearson's correlation between allelic fold change of an ASE locus and cell‐type enrichment score across samples.

### Colocalization Between ieQTL and GWAS Loci

To identify shared association variants between the ieQTL and GWAS loci retrieved from the PigGTEx project, colocalization analysis was performed using the Bayesian statistical procedure implemented in Coloc (v5.1).^[^ ^105^ ^]^ Briefly, the summary statistics of ieQTL was used for each ieGene and its matched GWAS loci as input for Coloc. This work only considered the GWAS loci with at least one SNP with a *p*‐value < 1 × 10^−5^. The posterior probabilities PP4 were obtained from the *coloc.abf* function with default parameters, where PP4 represents the probabilities of a shared variant affecting both the gene expression of a given cell type and the complex trait. ieGene‐trait pairs were defined with PP4 > 0.5 as significant colocalization. In addition, to compare whether eQTL differ from ieQTL in terms of colocalization with GWAS loci, the same pipeline was employed for ieQTL scanning to perform the colocalization analysis for eQTL for each ieGene and its matched GWAS loci as well.

### Genetic Mapping of Cell‐Type Specificity for Complex Traits in Pigs and Humans

To uncover associations of traits with cell types, an enrichment analysis of significant GWAS loci and cell‐type specific genes was performed using the LOLA (v1.22.0) R package.^[^ ^106^ ^]^ Specifically, this work extracted the top 200 cell‐type‐specific genes sorted in ascending order by the *p*‐value for each of the 19 available tissues and created an annotation based on the genomic regions of these candidate genes for each cell‐type tissue pair. The GWAS summary statistics of 268 pig complex traits were then examined and significant genetic variants were selected with *p* < 5  ×  10^−8^ for each trait, using all tested SNPs of the 268 GWAS summaries as the background set. Finally, the significance level (*p*‐value) of the enrichment fold was calculated using Fisher's exact test with FDR correction and defined trait‐tissue‐cell type trios with *p*‐value < 0.05 as significant enrichment. Furthermore, The enrichment analysis was expanded to a larger Duroc population (>26 000 individuals) from a commercial company, given that the current GWAS dataset is relatively small. The heritability enrichment analysis was performed for backfat thickness and loin muscle depth traits with genomic partitioning of quantitative genetic variance similar to the aforementioned GWAS results.^[^ ^107^ ^]^ A total of 11.7 M imputed variants that had been quality‐controlled were grouped into two sets: one containing variants within ±10 Kb of the top genes specific to each cell type, and the other containing the remaining variants. Per‐variant heritability enrichment was calculated for each cell‐type specific variant set.

To test the enrichment of genes associated with human traits and diseases for each specific pig cell type, the GWAS summary statistics of 137 human complex traits was collected from the UK Biobank and public literature (Table S9, Supporting Information). The cell‐type‐specific genes in pigs were converted to the corresponding human orthologous genes with one‐to‐one mapping with the Ensembl database.^[^ ^76^ ^]^ A total of 20 888 pairs of pig‐human orthologous genes had expression information in the dataset used, including 16 115 one‐to‐one orthologous pairs (15 354 protein‐coding genes used in this work), 2985 one‐to‐many pairs, and 1 788 many‐to‐many pairs. One‐to‐one orthologous correspondence accounts for the majority of gene repertoires and shares more robust alignment and clear conserved functions among different species, as compared to the other two types. In consequence, only 16 115 one‐to‐one orthologous genes were kept for subsequent analysis although a small proportion of genes would be lost. Finally, linkage disequilibrium (LD) score regression analysis (https://github.com/bulik/ldsc)^[^ ^108^, ^109^ ^]^ was employed to partition the heritability based on 262 annotations, including 261 cell‐type‐specific gene lists and one base annotation including all SNPs. Heritability enrichment was calculated as the proportion of trait heritability contributed by SNPs in the annotation over the total proportion of SNPs in that annotation. This work reported the coefficient *p*‐value as a measure of the association of each cell type with the traits. All plots showed the predicted *z*‐score of partitioned LD score regression.

### Cross‐Species Single‐Cell Transcriptomic Analysis

To understand the cellular component and transcriptomic dynamics across species, cross‐species integration of single‐cell/nucleus RNA sequencing data from human,^[^ ^2^ ^]^ monkey,^[^ ^5^ ^]^ mouse,^[^ ^1^ ^]^ and pig (Wang et al.^[^ ^27^ ^]^ and dataset) was performed. A total of 10 882 one‐to‐one orthologous genes were first retrieved across the four species for subsequent analysis using the Ensembl BioMart tool.^[^ ^76^ ^]^ Then, raw cell count matrices of the orthologous genes were extracted from the scRNA‐seq data for each species respectively, and concatenated as input to the Seurat package.^[^ ^91^ ^]^ Cross‐species integration was conducted using the Harmony strategy in the Seurat. All downstream analyses were carried out in a similar way as described above. To further quantify the interspecies transcriptional similarity for the different cell types, the MetaNeighbor framework^[^ ^75^ ^]^ was employed to calculate the AUROC scores across species. The pair‐wise correlations were conducted based on the AUROC scores.

### Statistics and Reproducibility

If not specified, all statistical analyses and data visualization were performed in the R environment. No statistical method was used to predetermine sample size, no data were excluded from the analyses, and all analyses were not randomized, ensuring maximum reproducibility.

## Conflict of Interest

The authors declare no conflict of interest.

## Author Contributions

L.C., H.L., and J.T. contributed equally to this work. G.Y. and L.F. conceived and designed the study. X.Q., Z.C., and L.Y. were responsible for sample collection. L.C., H.L., J.T., Z.W., X.C., Jinghui L. H.Z., Z.B., and J.J. conducted bioinformatic analysis. L.C. and Z.W. performed snRNA‐seq analyses. H.L., X.P., and J.T. contributed to eQTL mapping and cellular deconvolution. J.T., X.C., J.L., J.J., Z.Z., and J.L. were responsible for GWAS data collection and analysis in pigs and humans. G.Y., H.L., J.T., and J.L. wrote the initial draft of the manuscript. G.Y., L.F., J.J., G.E.L., F.W., L.L., Y.L., G.S., M.S.L., M.B., D.C.P., P.K.M., M.F., A.C., M.A., C.L., C.K.T., and O.M. revised the manuscript. All authors read and approved the final manuscript.

[Correction added on December 1, 2025, after first online publication: author contribution statement is added.]

## Supporting information



Supporting Information

## Data Availability

The data that support the findings of this study are openly available in GEO at https://www.ncbi.nlm.nih.gov/gds/?term=GEO, reference number 233285.
