# Supplementary material for: Construction of a Multitissue Cell Atlas Reveals Cell‐Type‐Specific Regulation of Molecular and Complex Phenotypes in Pigs
Source: Adv Sci (Weinh). 2025 Nov 27;13(8):e04961. doi: 10.1002/advs.202504961 (PMC12884822; doi:10.1002/advs.202504961)
Supplement: Supplementary file 1 — Supporting Information [file ADVS-13-e04961-s001.docx]

Supplementary Figures:


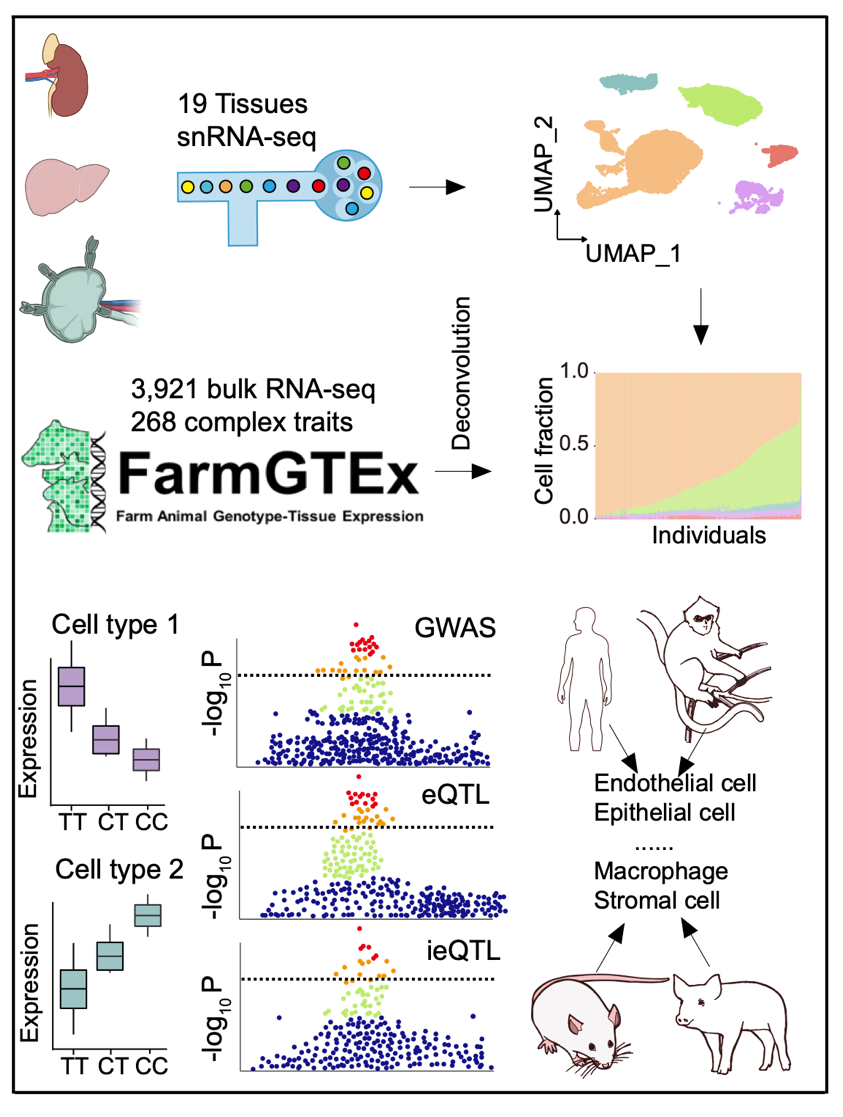


**Figure S1. Overview of the study workflow.** We performed snRNA-seq data analysis across 19 tissues in pigs and deconvoluted 3,921 bulk RNA-seq samples from PigGTEx. We identified cell-type interaction expression QTL (ieQTL) and leveraged our results to co-localize with GWAS results of 268 complex traits. The cartoons used to generate this illustration from https://bioart.niaid.nih.gov/.


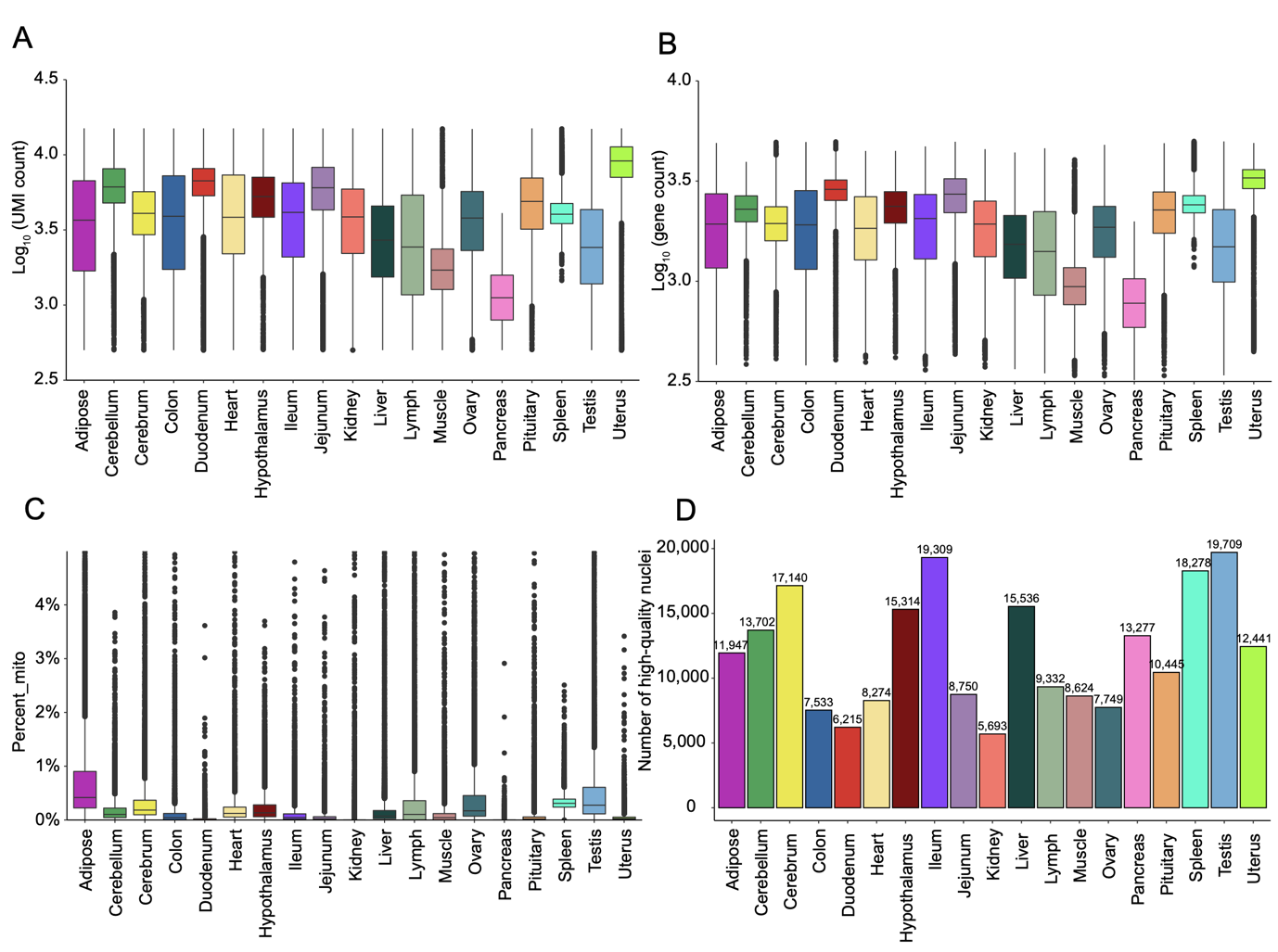


**Figure S2. Quality control of cross-tissue snRNA-seq data.**

**(A)** Boxplots showing the distribution of UMIs per nucleus for each tissue processed in this study. The horizontal line in the boxplots corresponds to the median, the box bounds indicate the 25th and 75th percentiles, and the whiskers represent 1.5 times the interquartile range. Values outside the whiskers are displayed as points.

**(B)** Boxplots showing the distribution of detected genes per nucleus for each tissue.

**(C)** Boxplots showing the proportion of mitochondria reads per nucleus for each tissue.

**(D)** Barplot showing the number of high-quality nuclei in 19 tissues after filtering.


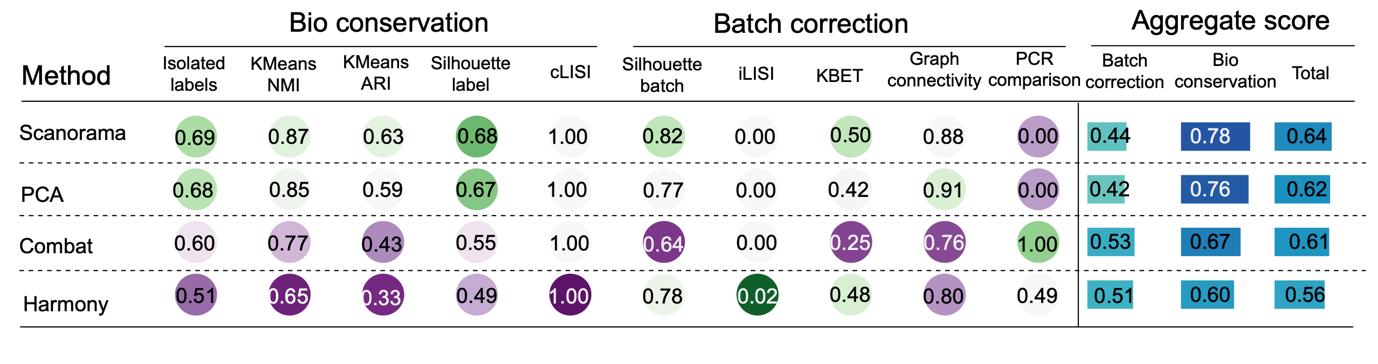


**Figure S3: Performance assessment of snRNA-seq data integration**. Integration accuracy metrics are divided into two categories: batch effect removal and conservation of biological variance. Overall scores were computed using a 40:60 weighted mean of these two category scores.


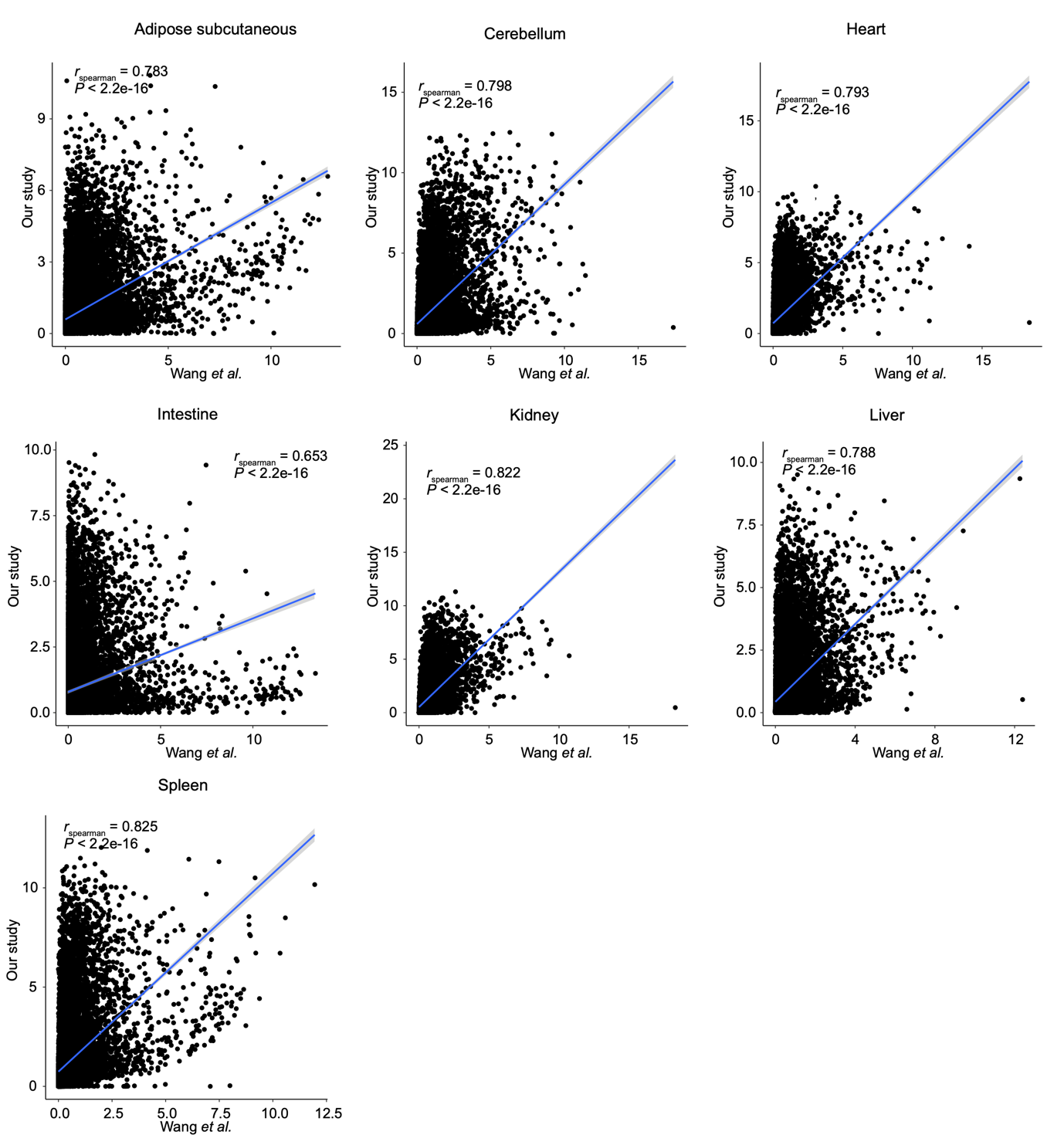


**Figure S4. Spearman correlation of normalized expression levels for all protein-coding genes across seven common tissues between the two single-cell RNA-seq studies in pigs.** We summarized the gene-wise expression of all high-quality cells by summing them up as the pseudo-bulk transcriptomic profiles of a tissue. The correlation coefficients are displayed in the top of each panel.


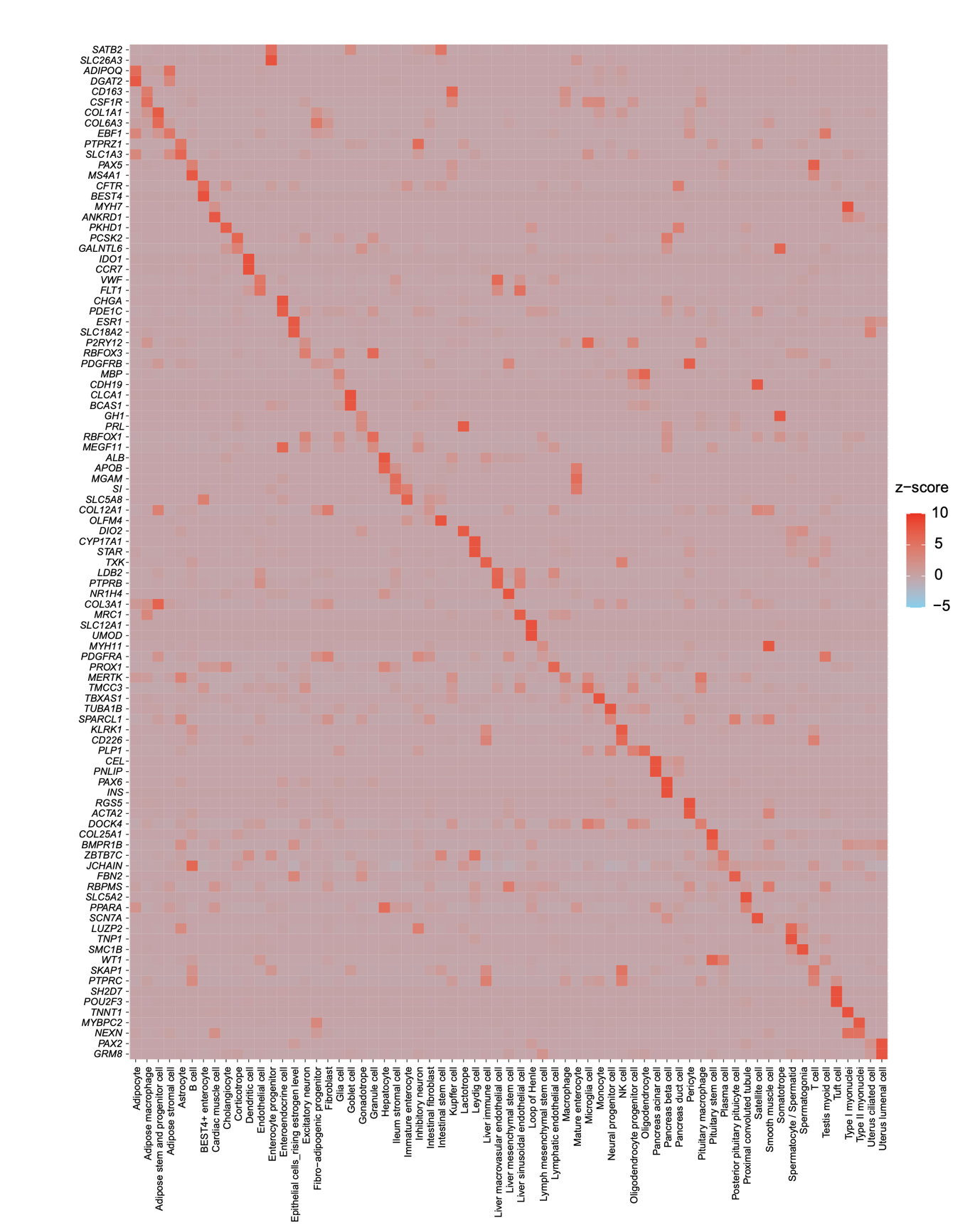


**Figure S5. Selected canonical marker genes for cell type annotation in pigs.** Heatmap showing the expression levels of representative marker genes used to manually annotate all cell clusters in the present dataset.


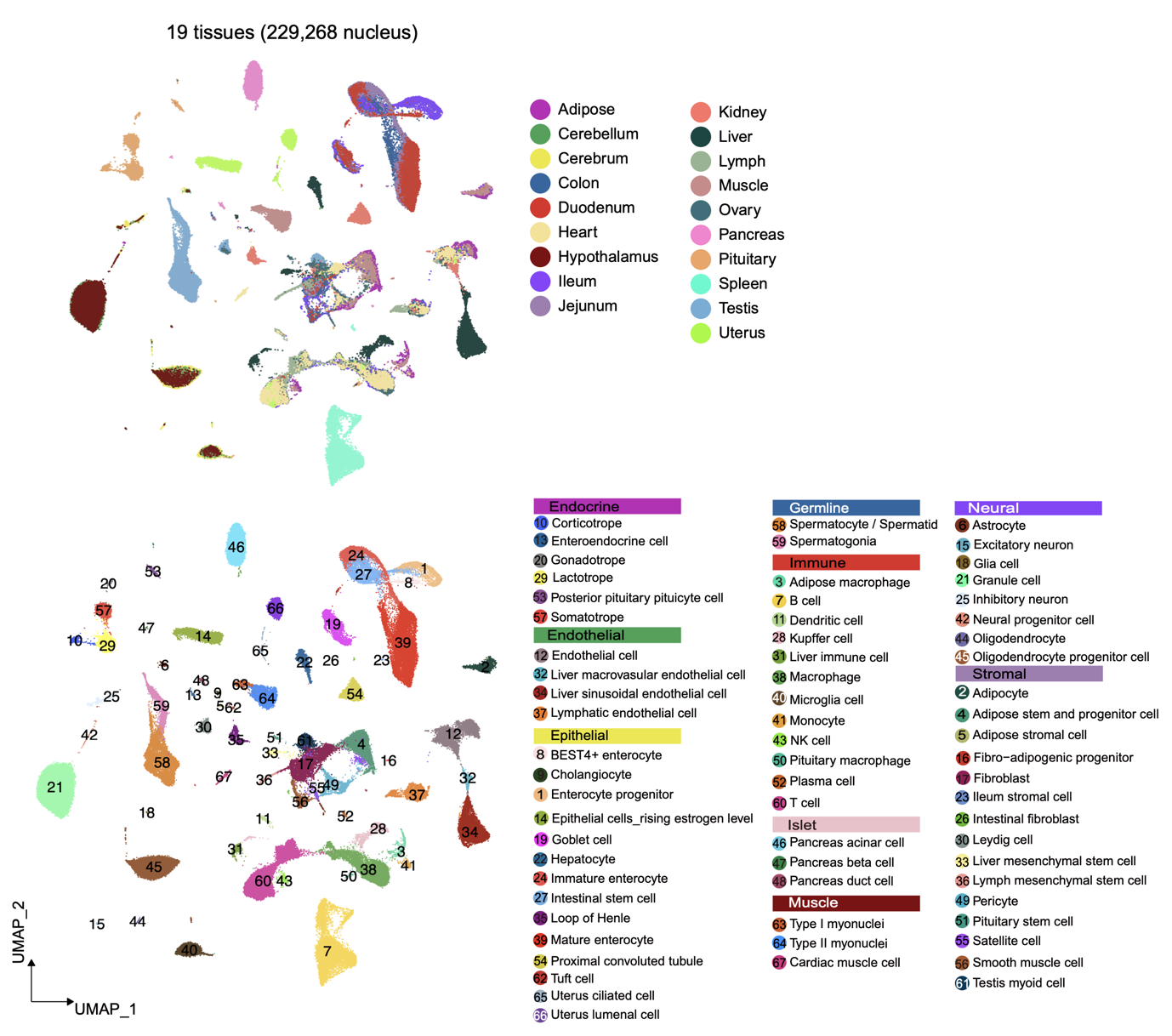


**Figure S6. UMAP visualization of single-nucleus profiles (dots) colored by tissues and major cell types.** All cell types are categorized into nine top-level cell lineages, and cell type annotation is provided in the legend to the right.
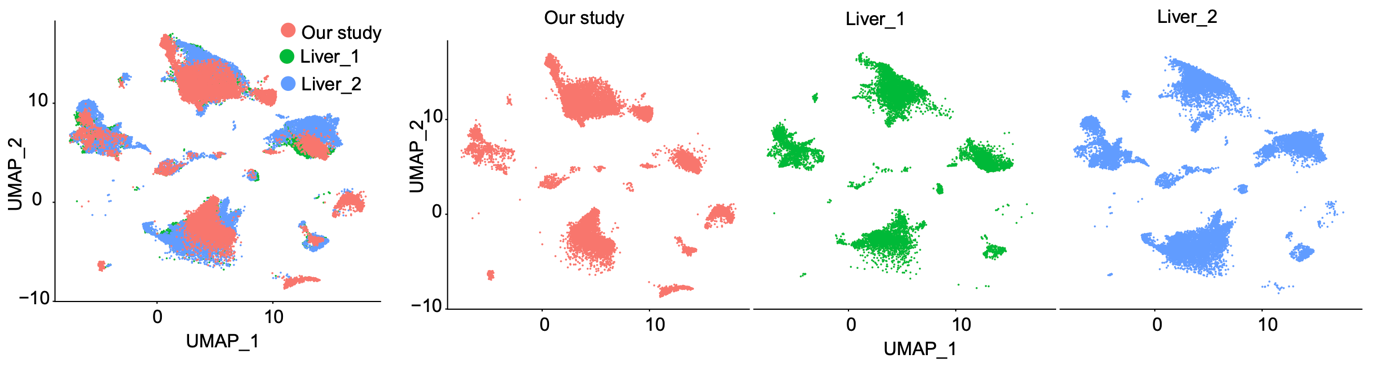


**Figure S7. High overlap between our study and two additional biological replicates in pig liver tissue.** Liver_1 and Liver_2 represented two biological replicates.


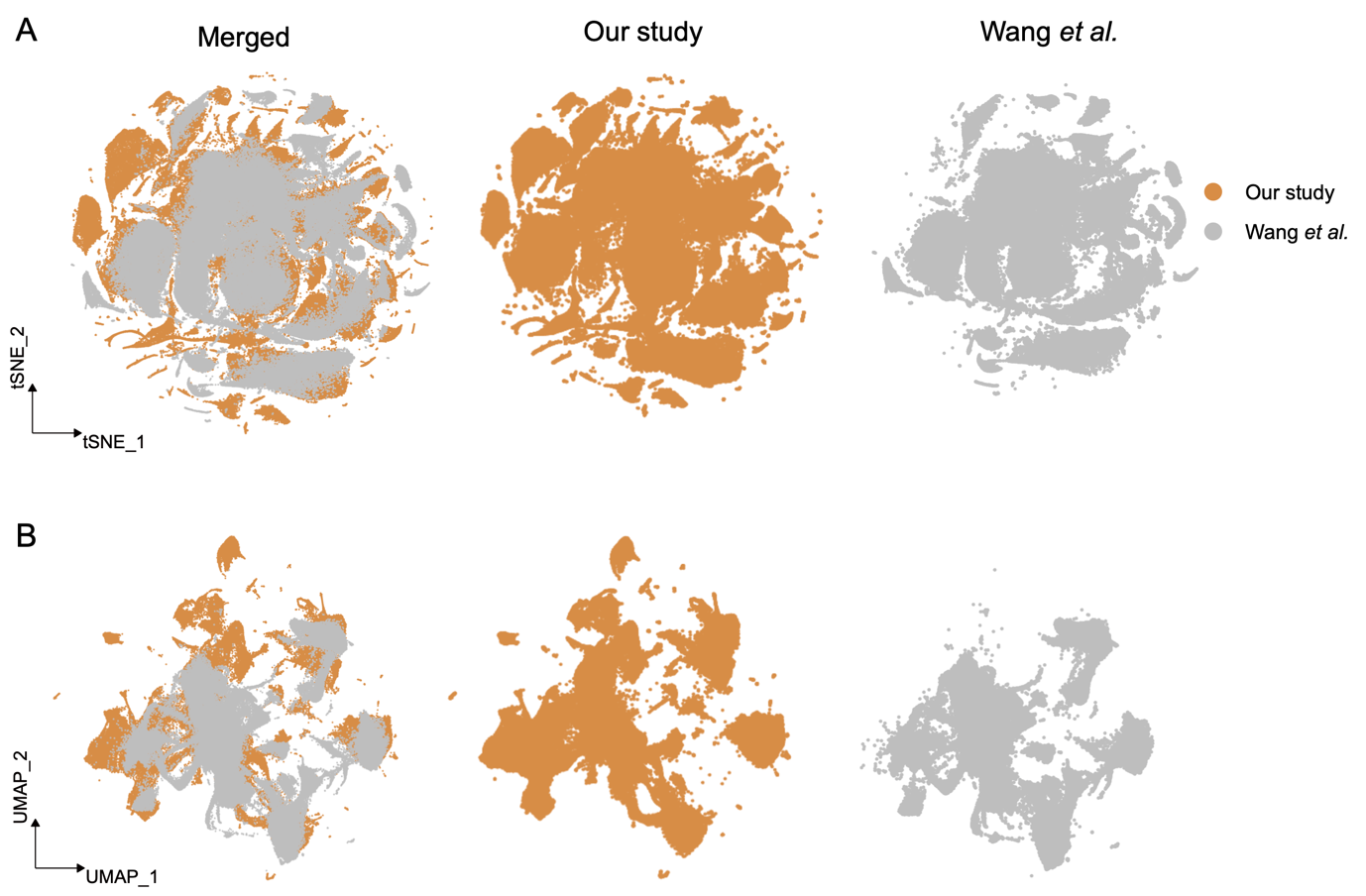


**Figure S8. Integrated cell maps combining single-nucleus/cell datasets from Wang *et al*. and our work.**


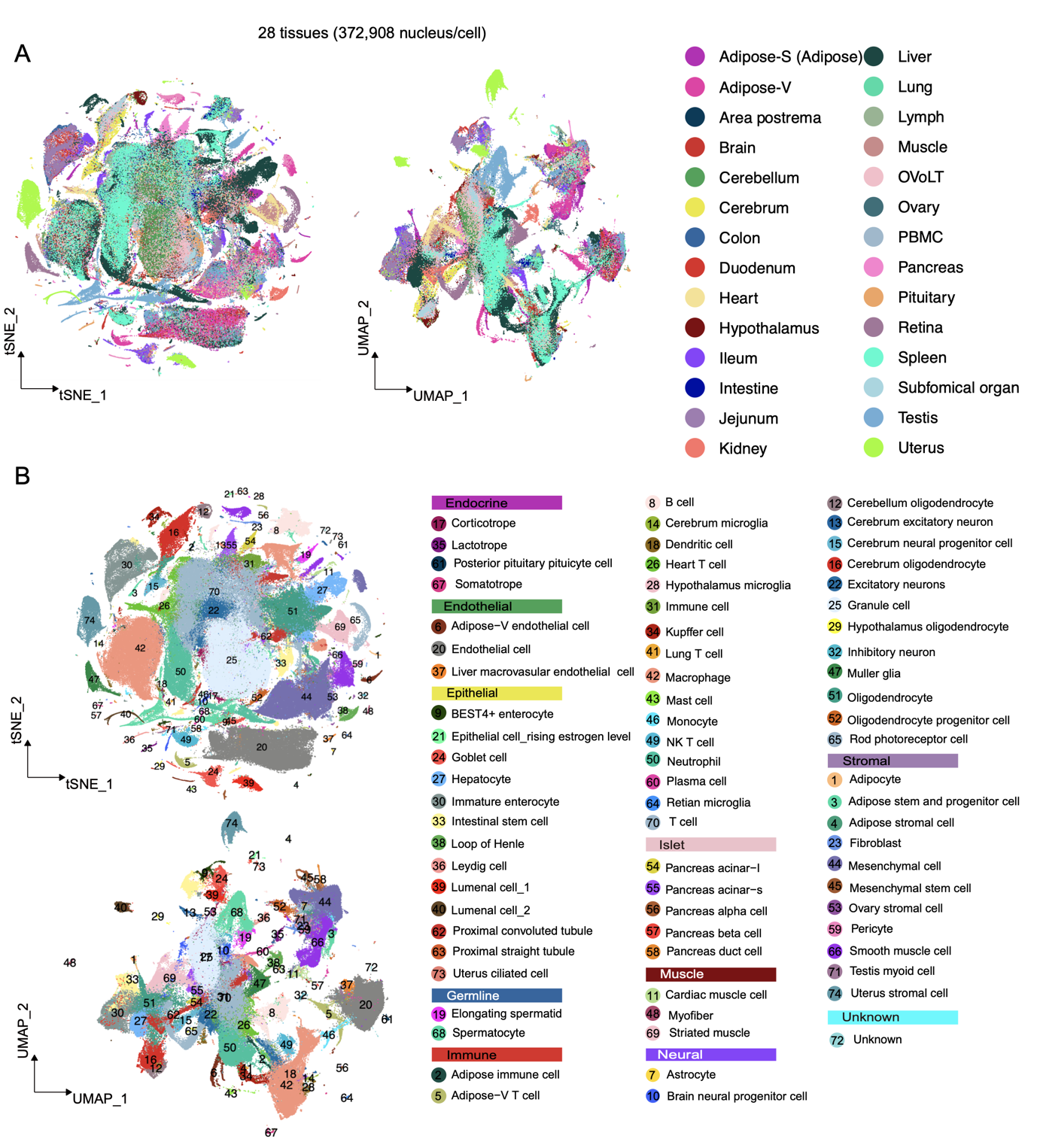


**Figure S9. t-SNE and UMAP visualization of single-nucleus profiles (dots) colored by tissues and major cell types.** All cell types from integrated cell map are categorized into nine top-level cell lineages, and cell type annotation is provided in the legend to the right.

**
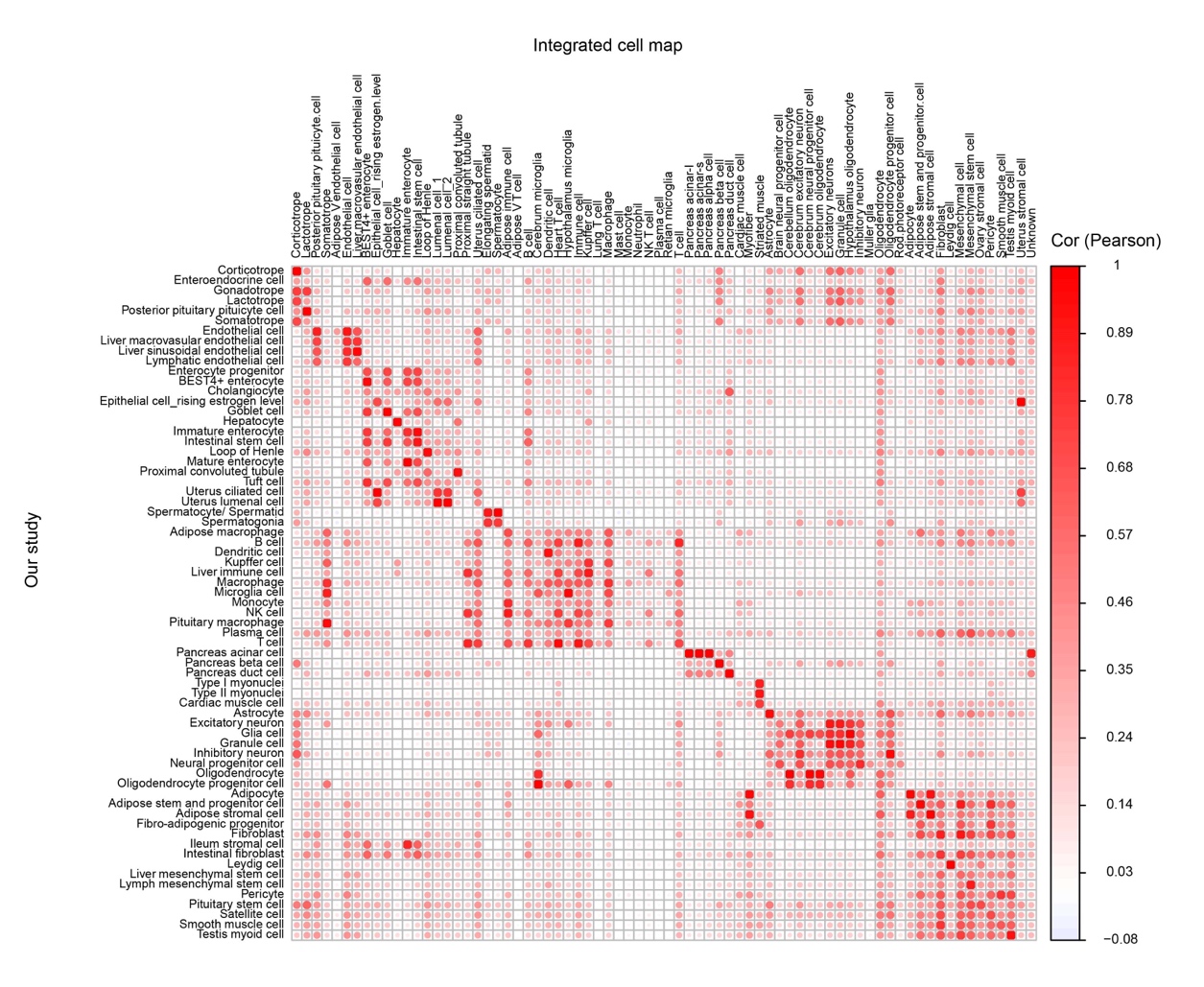
**

**Figure S10. Pair-wise correlation heatmap showing the similarity between cell types from integrated cell map and cell atlas from our data.**


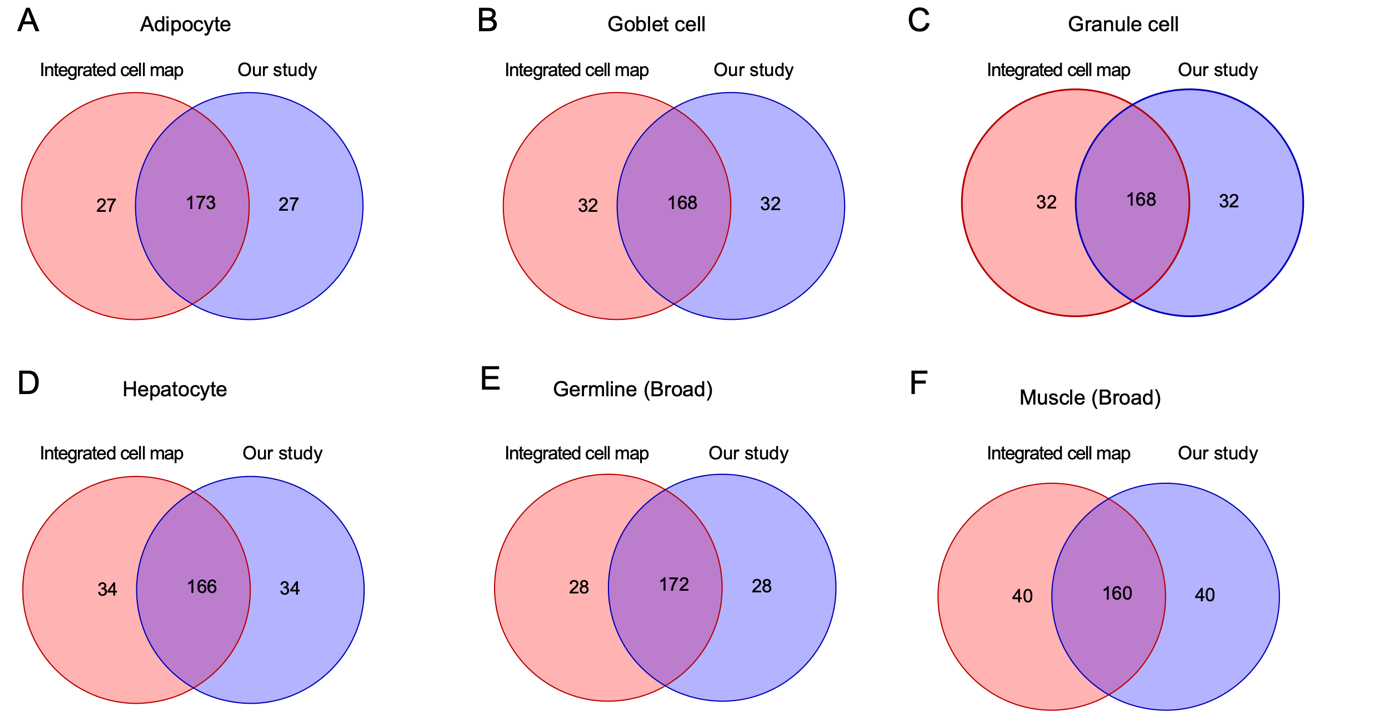


**Figure S11. Overlap between top 200 marker genes for the six representative cell types.**


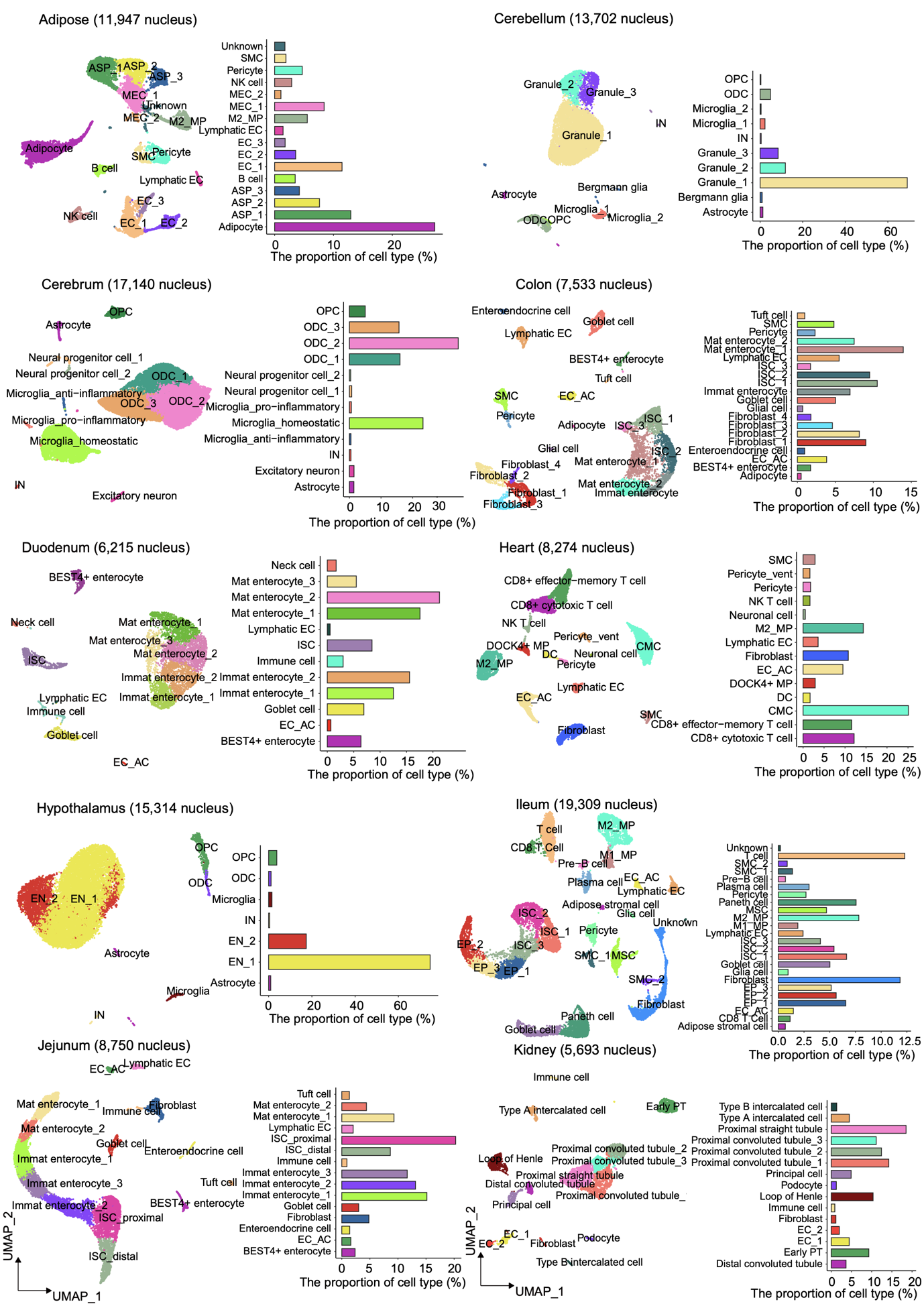


**Figure S12. UMAP visualization and the proportion of cell clusters in 10 tissues.** These tissues contain the adipose, cerebellum, cerebrum, colon, duodenum, heart, hypothalamus, ileum, jejunum, and kidney. The name and proportion of each cell population for every tissue are indicated, and definitions for abbreviations are provided in Supplementary Table S1.


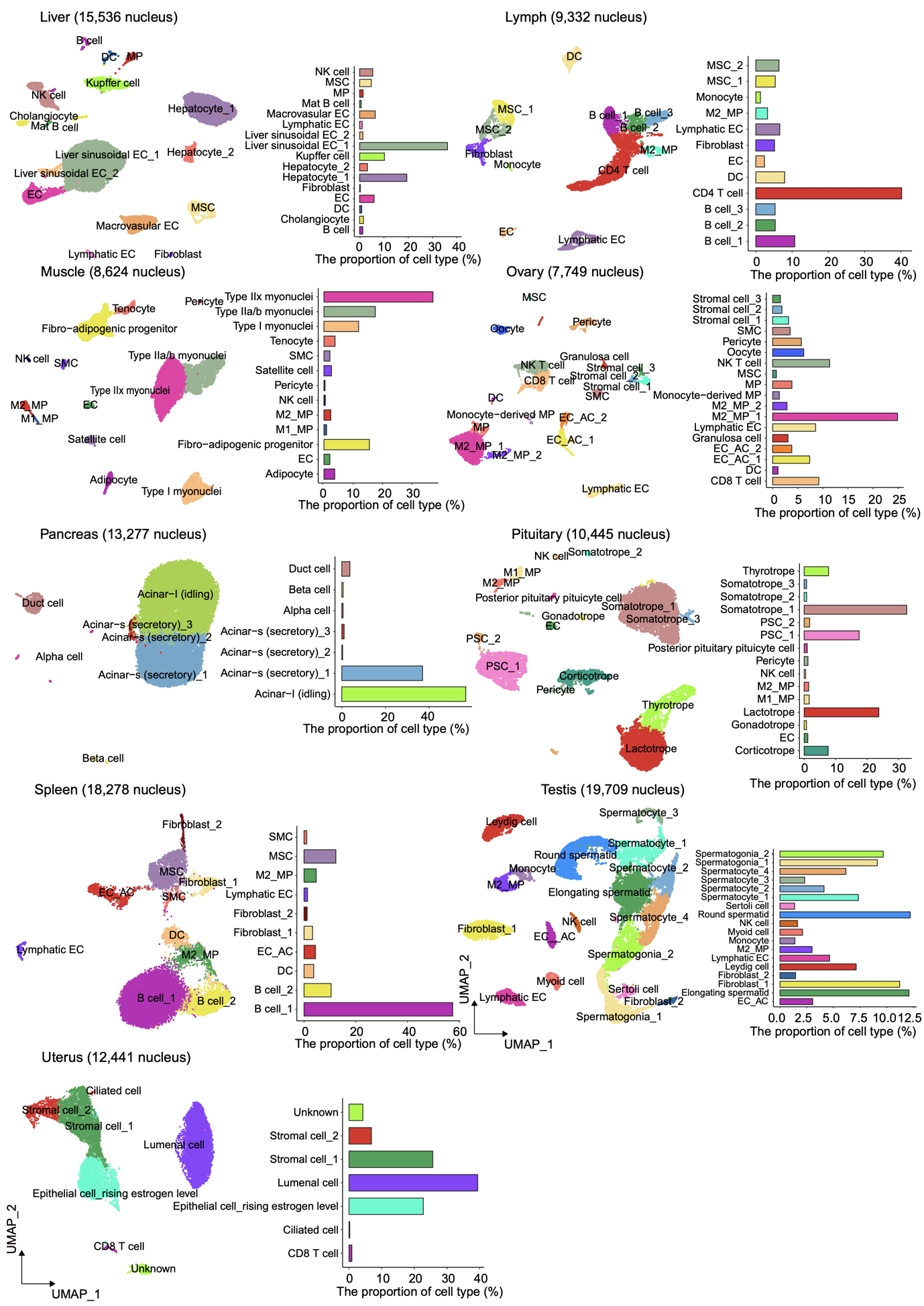


**Figure S13. UMAP visualization and the proportion of cell clusters in nine tissues.** These tissues contain the liver, lymph, muscle, ovary, pancreas, pituitary, spleen, testis, and uterus The name and proportion of each cell population for every tissue are indicated, and definitions for abbreviations are provided in Supplementary Table S1.


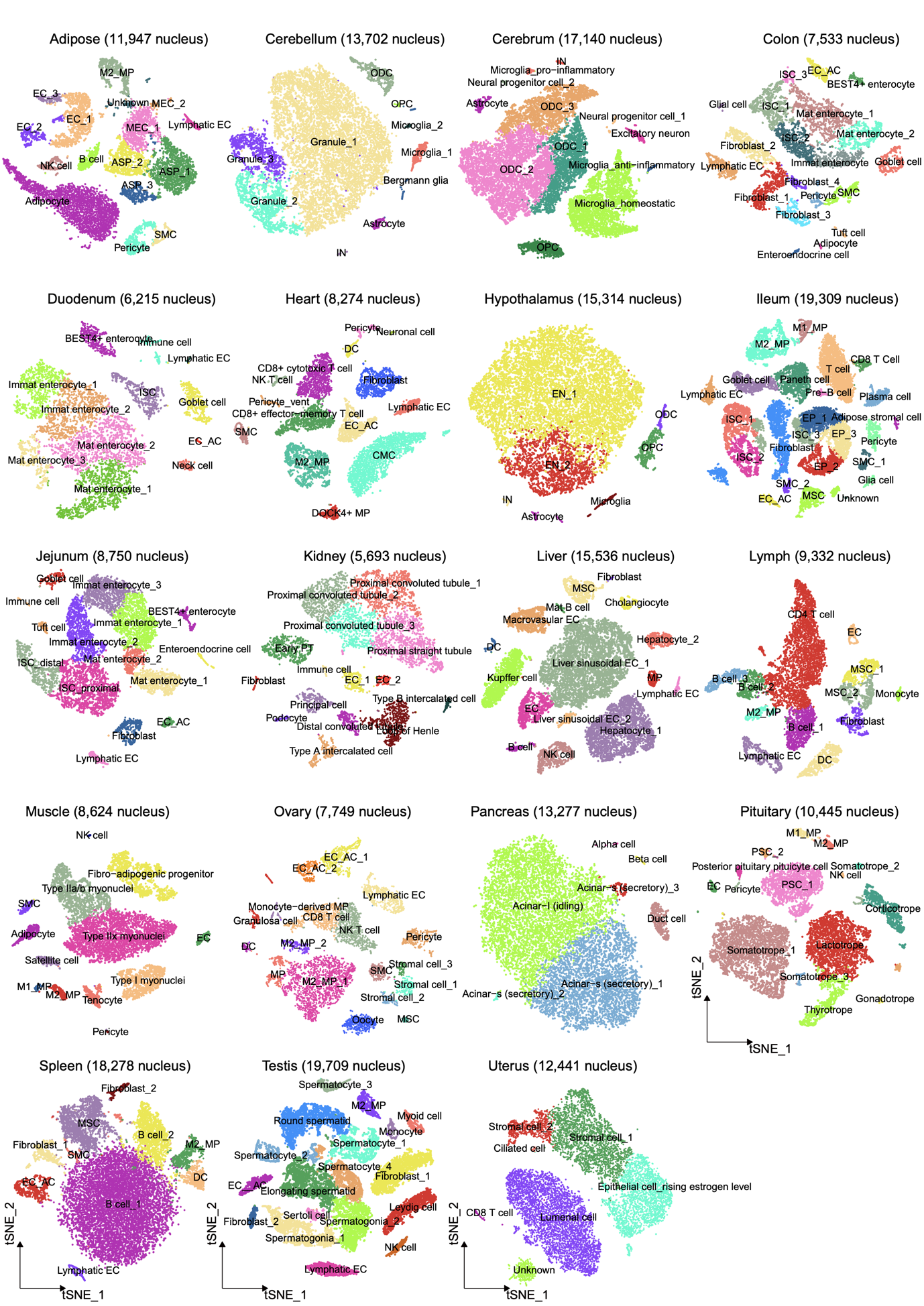


**Figure S14. t-SNE visualization showing cell atlas in 19 tissues.** The definitions for abbreviations are provided in Supplementary Table S1.


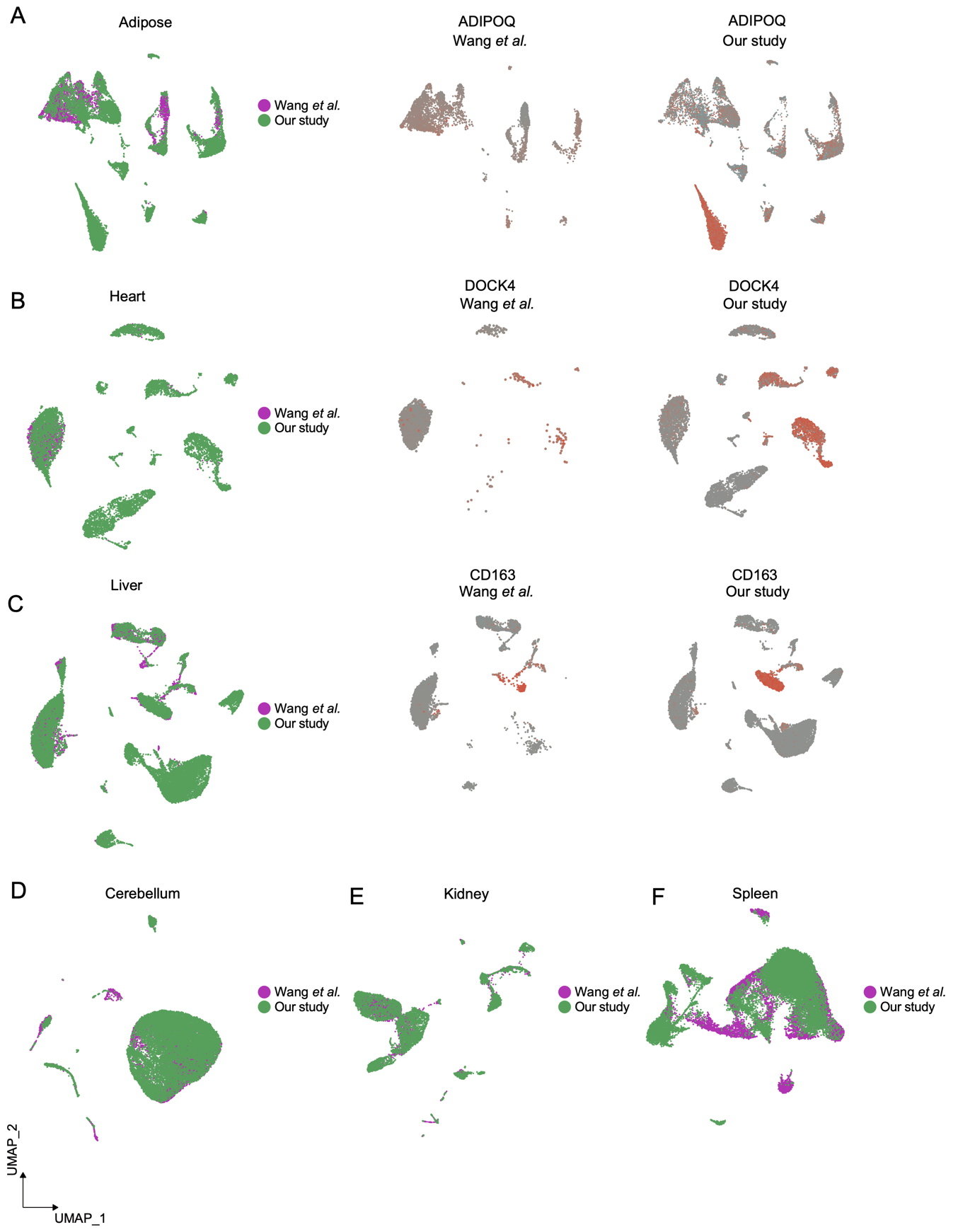


**Figure S15. Comparison of single-cell transcriptional patterns between our dataset and a public pig scRNA-seq study.**

**(A)** Single-cell transcriptional difference of the *ADIPOQ* gene in pig adipose tissue between two studies.

**(B)** Single-cell transcriptional difference of the *DOCK4* gene in pig heart tissue between two studies.

**(C)** Single-cell transcriptional difference of the *CD163* gene in pig liver tissue between two studies.

**(D-F)** Single-cell transcriptional difference in the cerebellum (**D**), kidney (**E**), and spleen (**F**) between two studies.


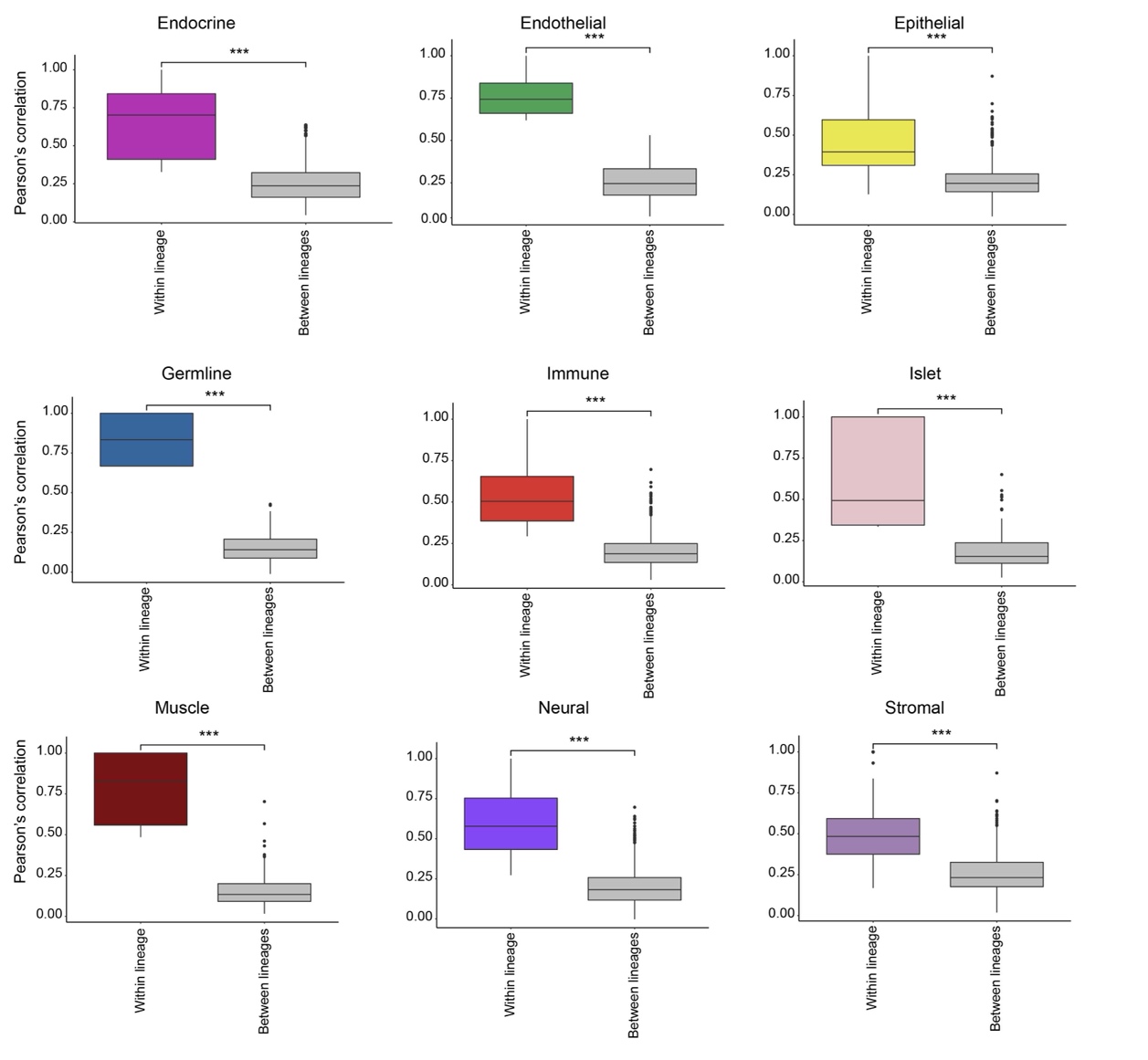


**Figure S16. Pair-wise correlation of transcriptional patterns between cell types within lineage and between lineages.**
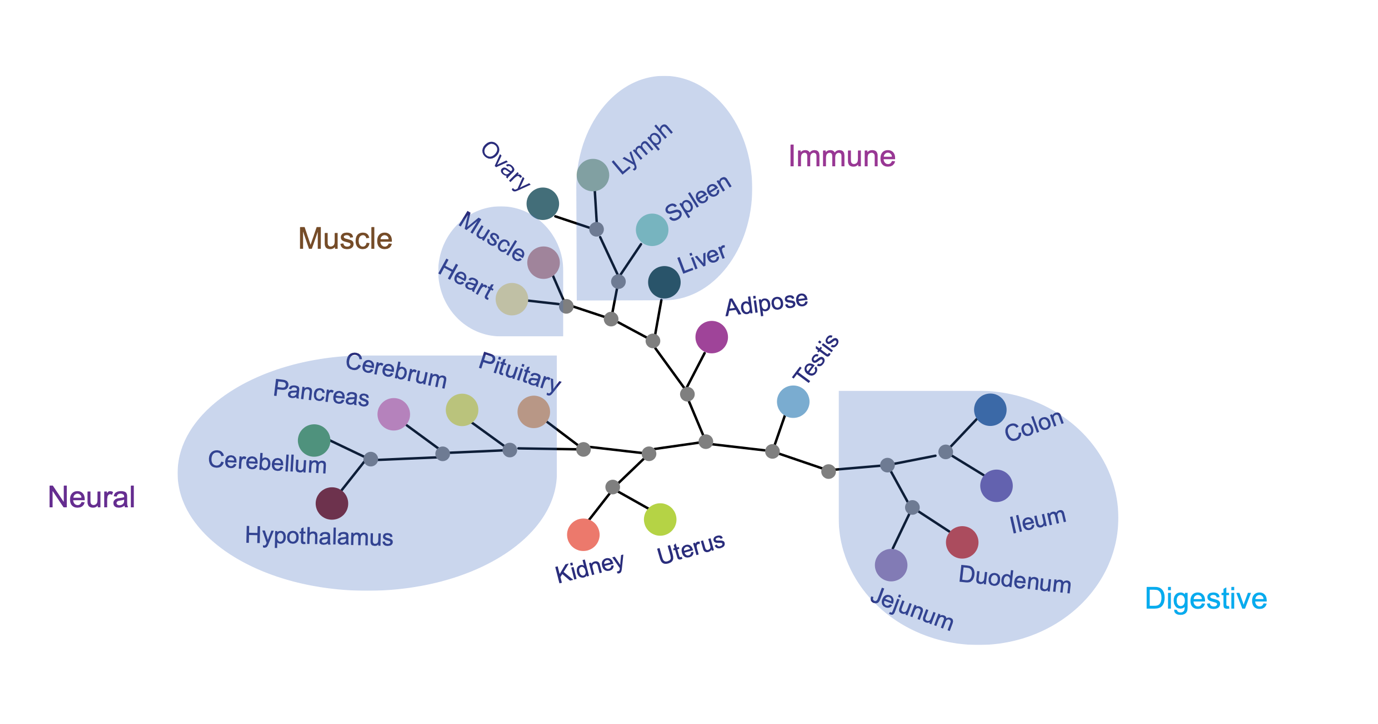


**Figure S17: Hierarchical clustering across 19 tissues.** Dendrogram showing hierarchical clustering of tissues based on pseudo-bulk gene expression profiles.


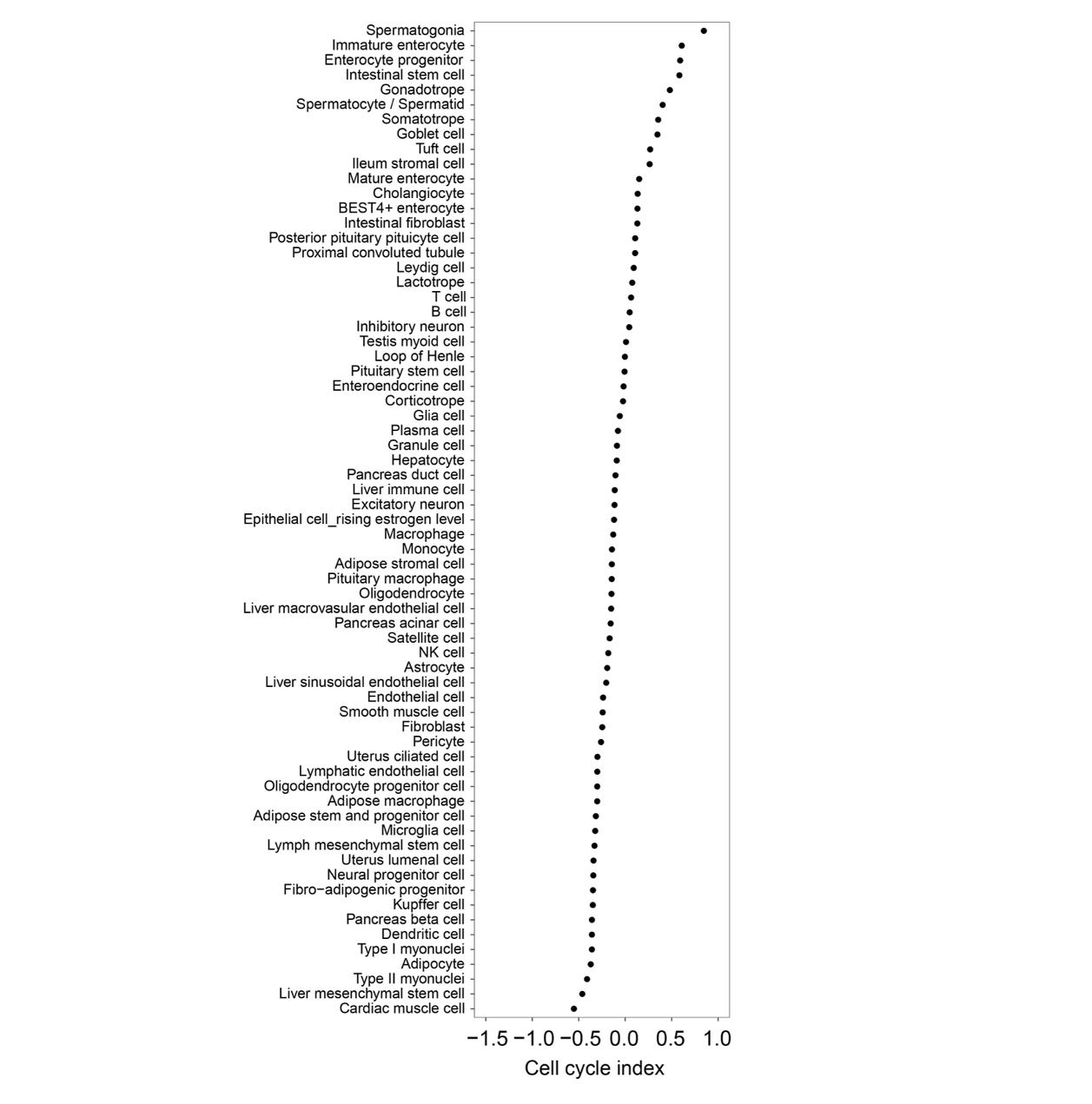


**Figure S18. Inferred cell state for each major cell type.** Cell types are ordered by the magnitude of the putative cell cycling index, with the most highly proliferative at the top and quiescent cells at the bottom of the list.


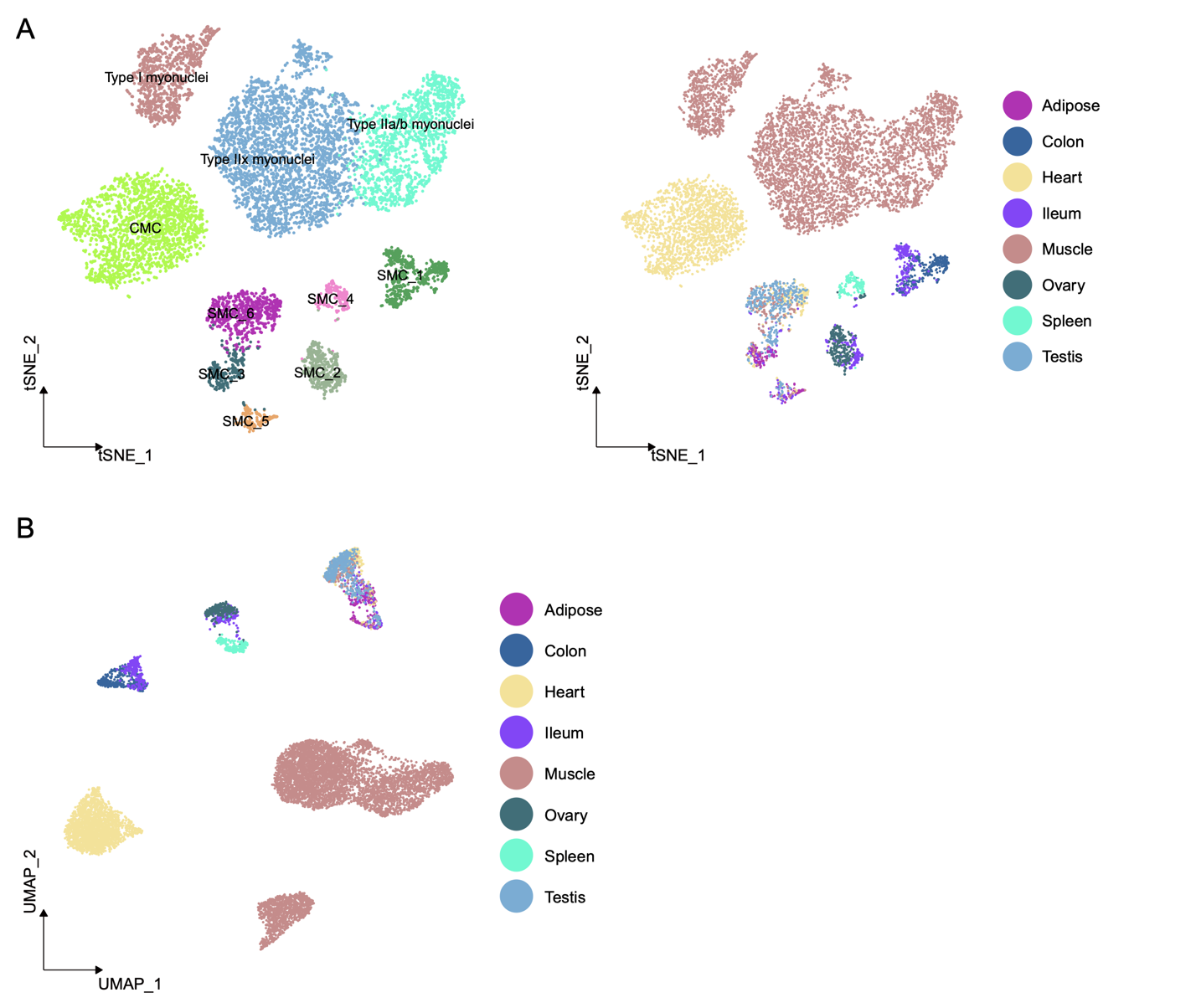


**Figure S19. t-SNE and UMAP visualization of single-nucleus profiles (dots) colored by tissues and cell types.** Each dot represents one nucleus, with colors coded according to manually annotated cell types which are assigned as muscle cells. CMC, cardiac muscle cell; SMC, smooth muscle cell.


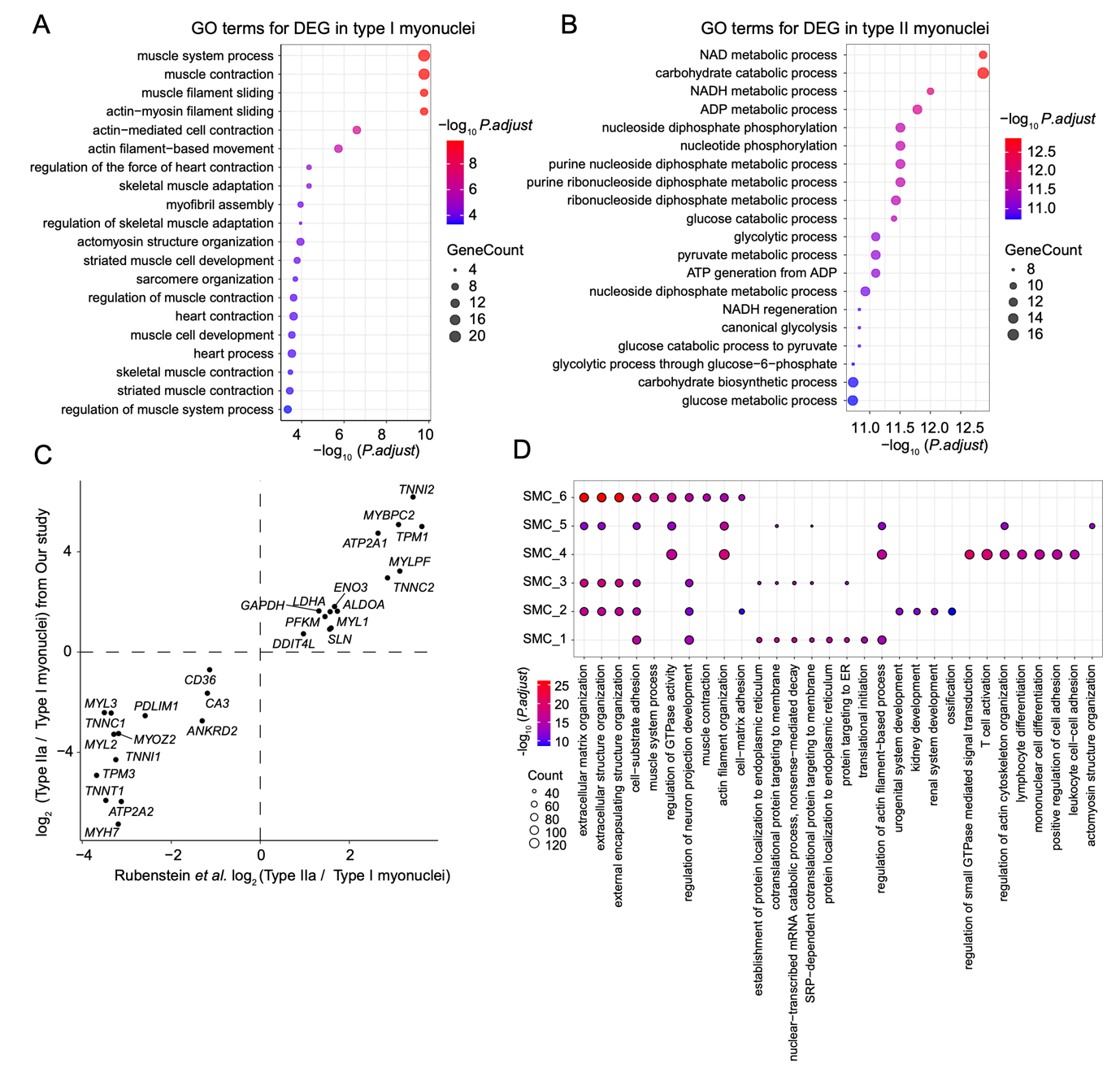


**Figure S20. Detailed characterization of type I and type II myofibers.**

**(A-B)** Functional annotation of differentially expressed genes between type I (**A**) and type II myofibers (**B**).

**(C)** Correlation of genes with the largest fold changes between type I and type II myofibers in pigs and humans. The candidate gene list was retrieved from Rubenstein *et al*..

**(D)** Functional enrichment for the six smooth muscle cell subtypes.


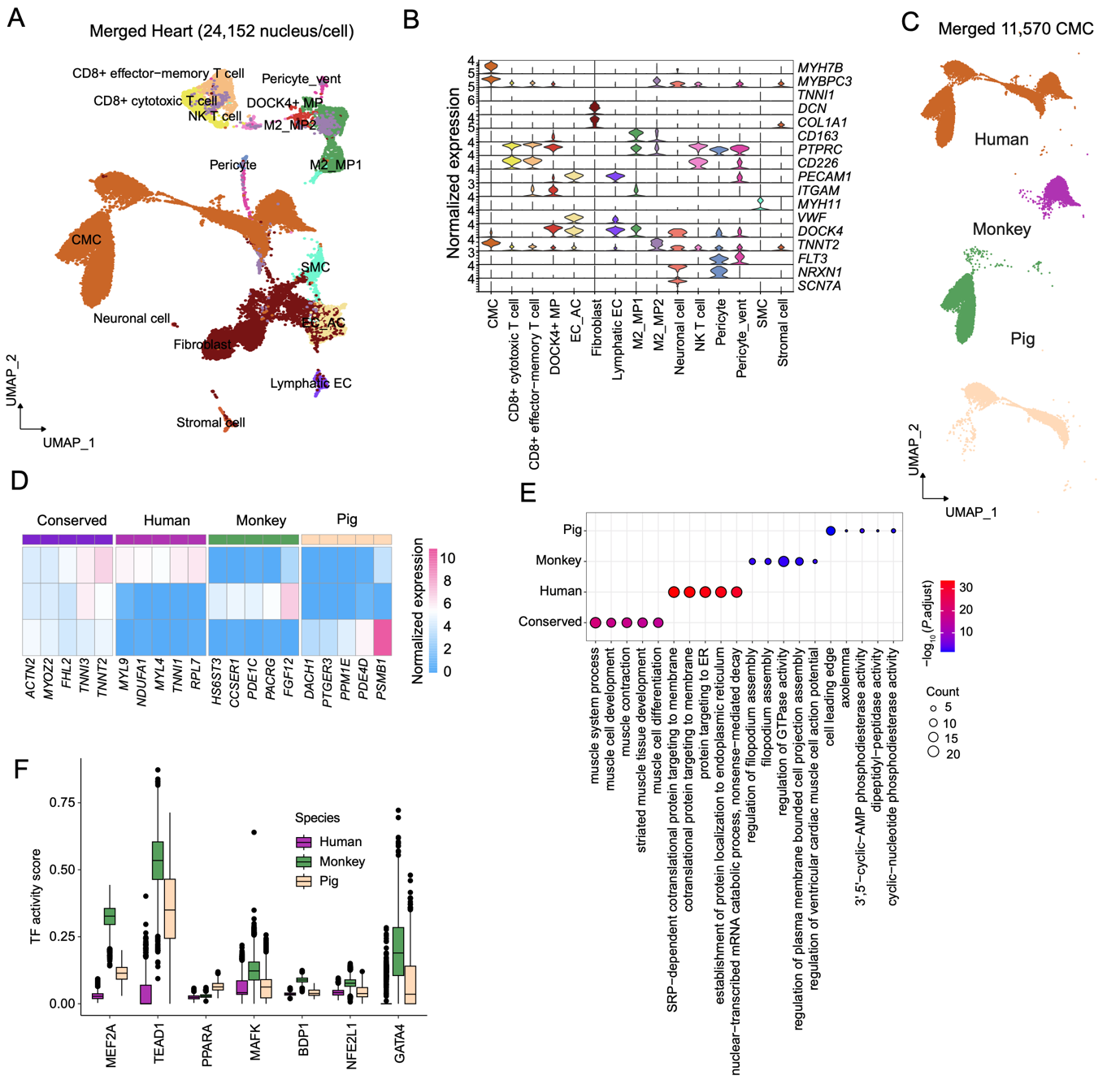


**Figure S21. Integrated analysis of heart tissue scRNA-seq/snRNA-seq data across species.**

**(A)** UMAP visualization of scRNA-seq/snRNA-seq profiles colored by cell types.

**(B)** Violin plots illustrating the expression of marker genes for each cell type.

**(C)** UMAP visualization of CMCs colored by cell type and species.

**(D)** Heatmap displaying the top five expressed genes for each module (conserved, human, monkey, and pig).

**(E)** Dot plot showing the top five GO enrichment terms for each module (conserved, human, monkey, and pig). Dot size represents gene count and is colored by adjusted *p*-values.

**(F)** Transcription factors exhibiting distinct activity scores across the three species.

**
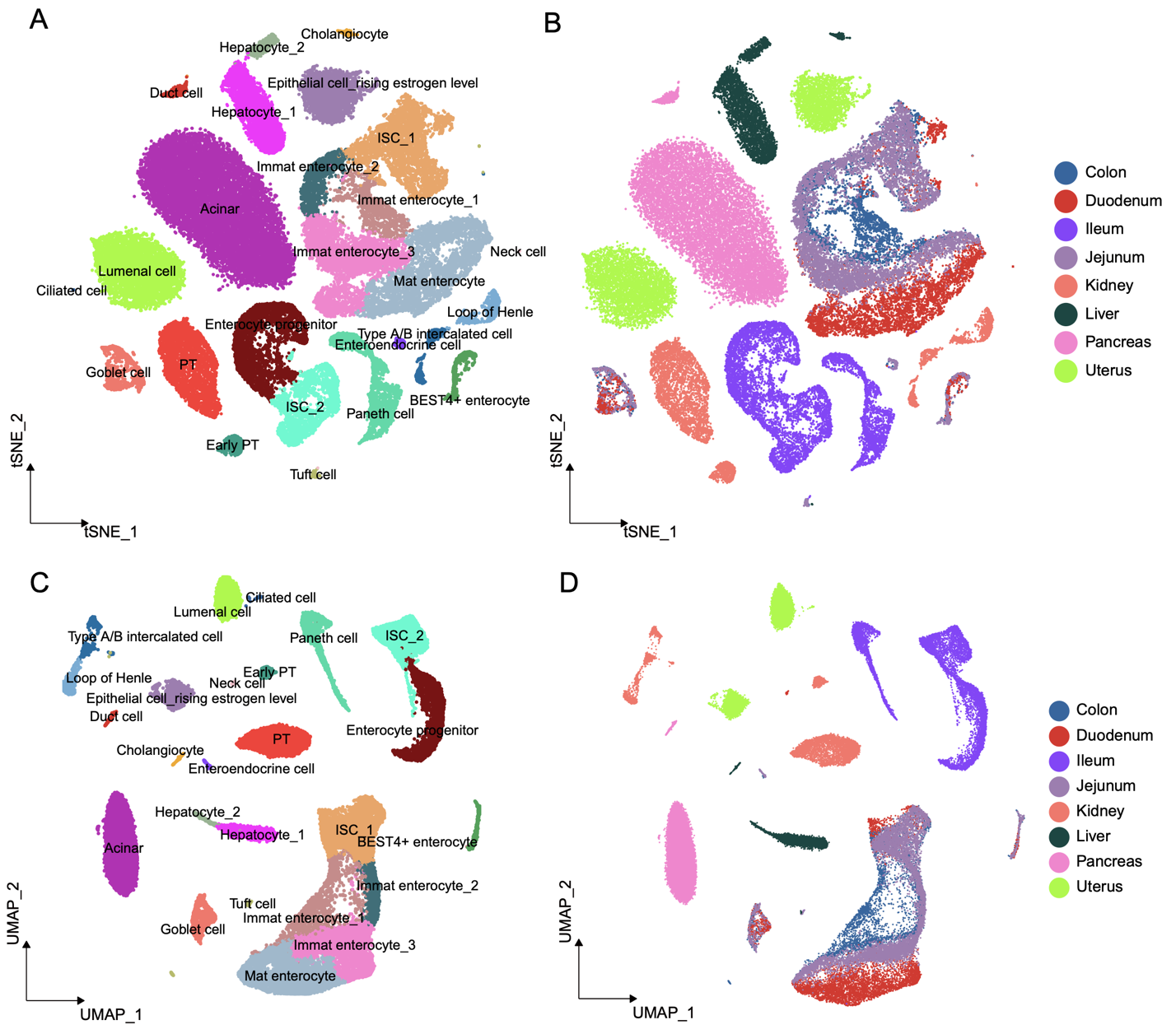
**

**Figure S22.** **The cell type annotation of epithelial cell clusters based on snRNA-seq data.** t-SNE and UMAP visualization showing cell distribution according to cell types (left) and tissues (right).


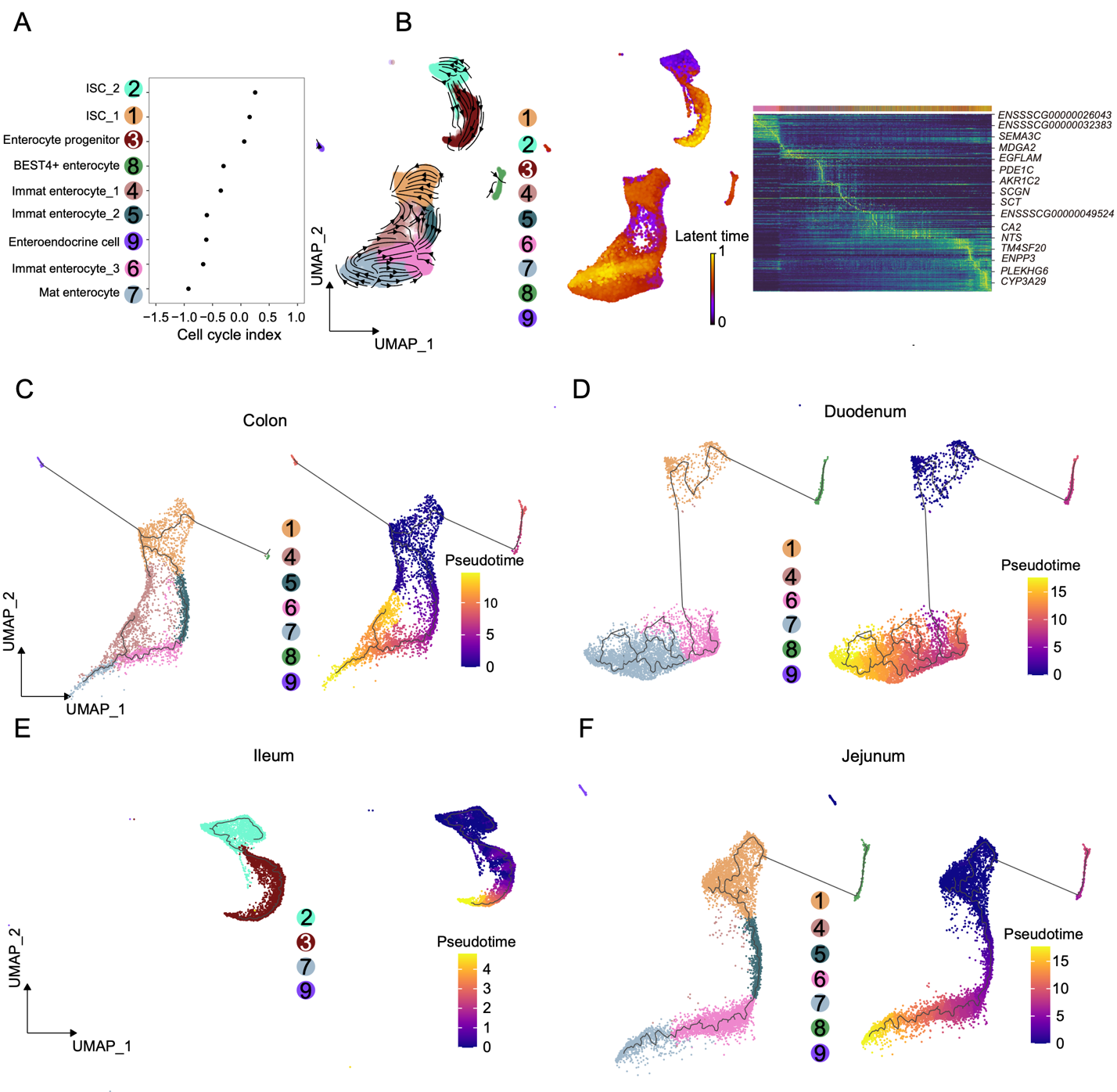


**Figure S23. Differentiation trajectory inference for selected cell types from four intestinal segments.**

**(A)** Cell cycle indices for various cell types from four intestinal segments.

**(B)** RNA velocity analysis demonstrating state transition for various cell types from four intestinal segments. The arrows represent a flow derived from the ratio of unspliced to spliced transcripts, which in turn predicts dynamic changes in cell identity. Heatmap on the right demonstrating stereotyped changes in gene expression trajectory.

**(C-F)** Pseudotime analysis of selected cell types from colon (**C**), duodenum (**D**), ileum (**E**), and jejunum (**F**). The trajectory is colored by cell subtypes (left) or pseudotime (right) for each panel.


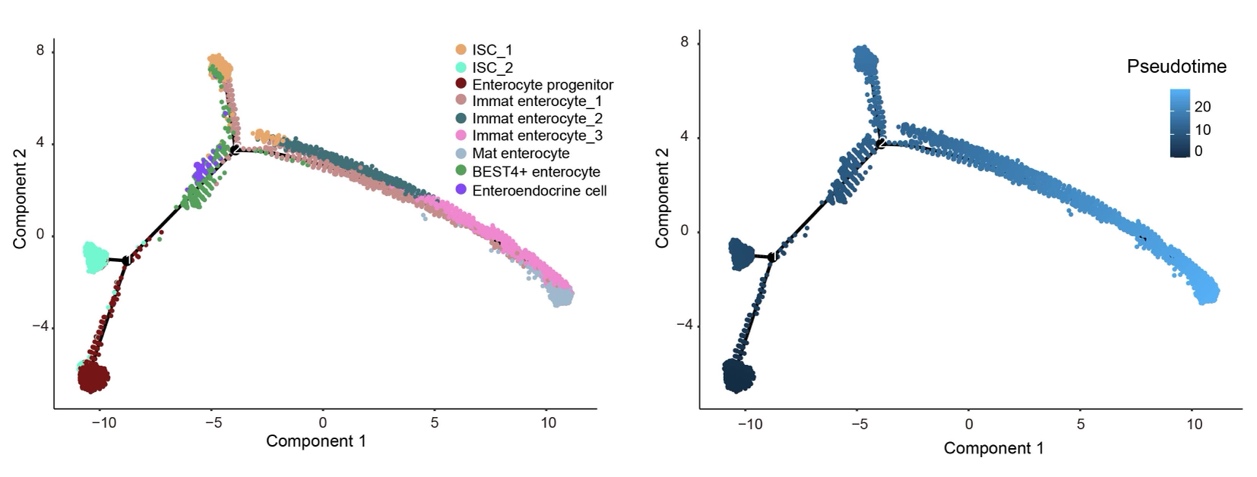


**Figure S24. Pseudotime analysis based on Monocle2 of the two intestinal stem cell subtypes.** The trajectory is colored by cell subtypes (left) or pseudotime (right) for each panel.


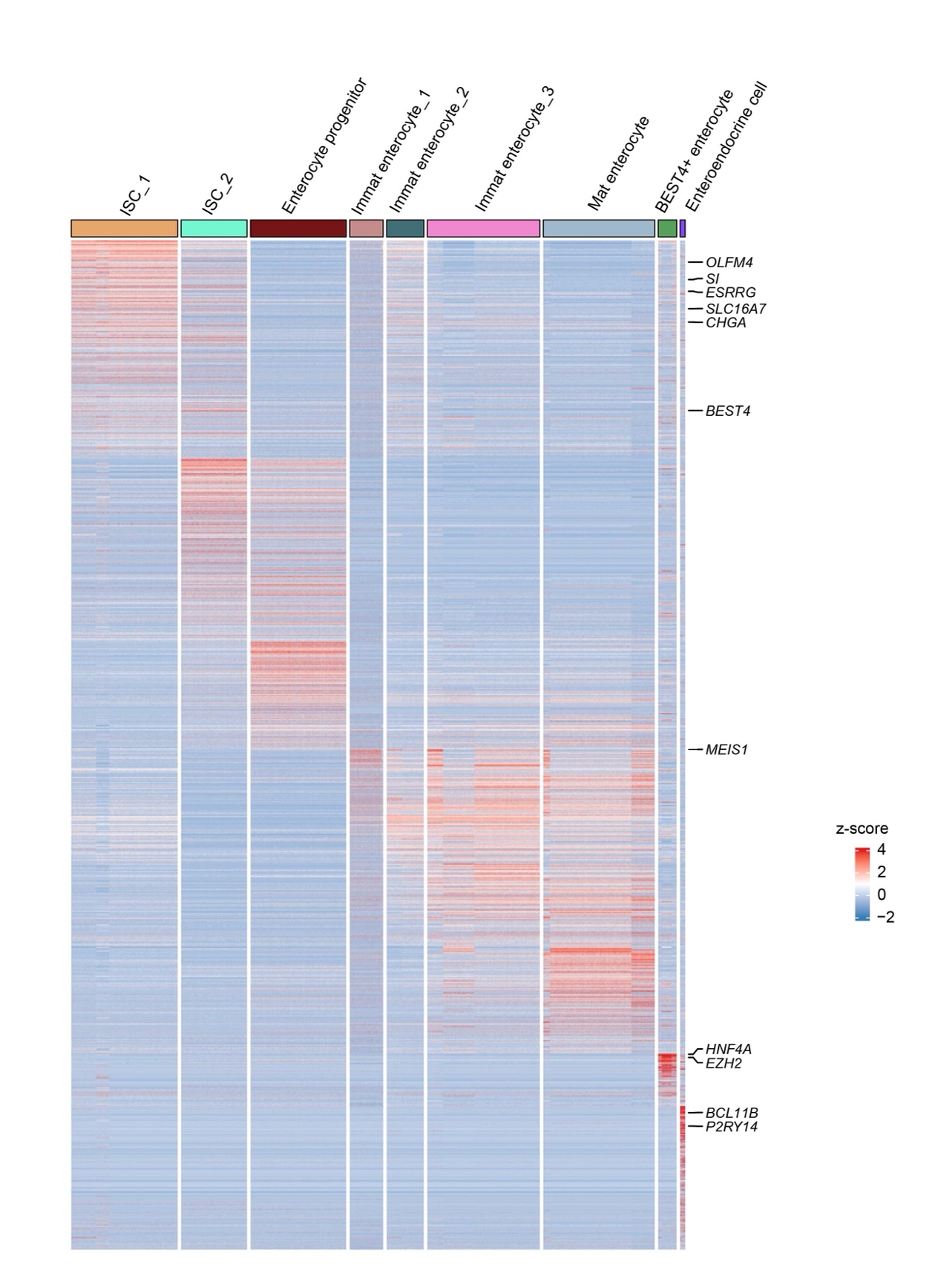


**Figure S25. Heatmap representation of genes showing cell-type-restricted expression patterns in the nine defined cell subtypes.** The top 20 differentially expressed genes for each cell subtype were extracted as the representative. Color represents normalized expression levels.


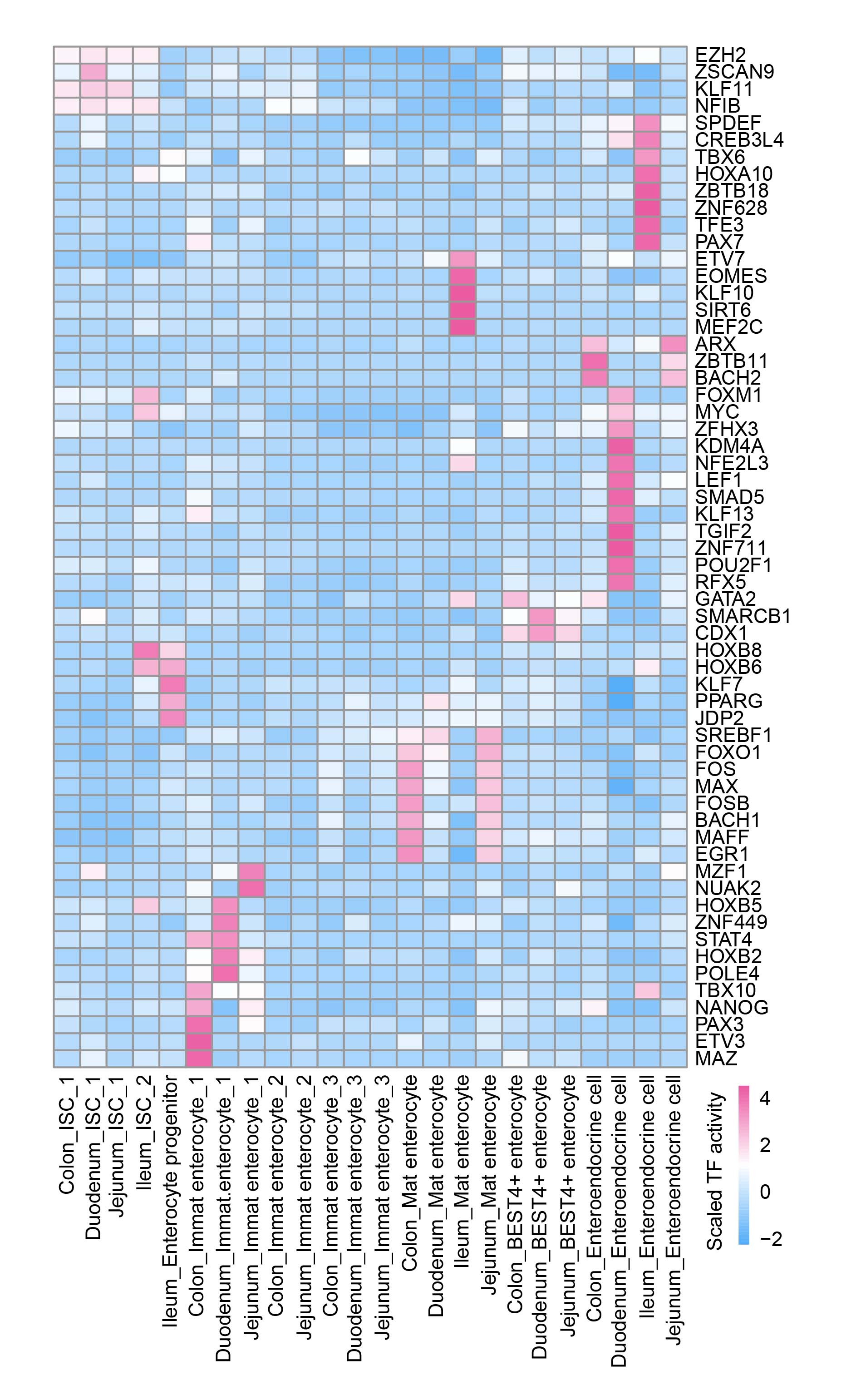


**Figure S26. Heatmap showing the activity scores of transcription factors for each cell subtype from four intestinal segments.** Each row represents one representative transcription factor, and each column represents one cell subtype.


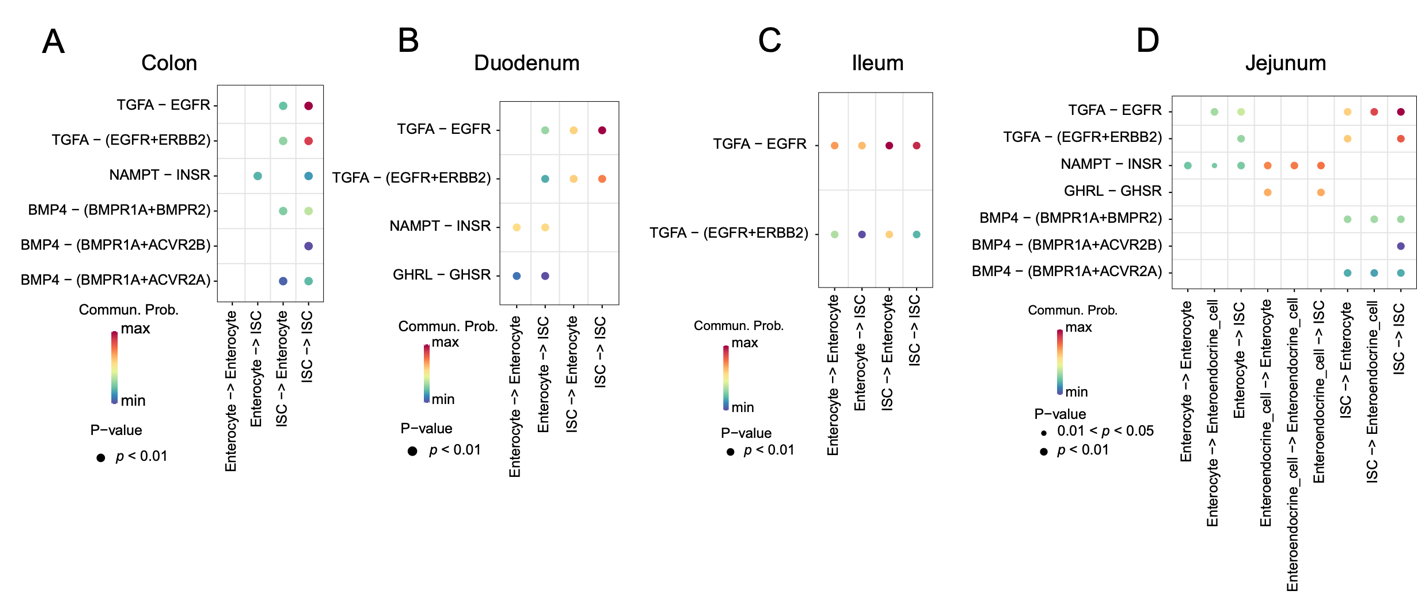


**Figure S27. Dot plots depicting representative ligand-receptor interactions between various cell subtypes from four intestinal segments.**


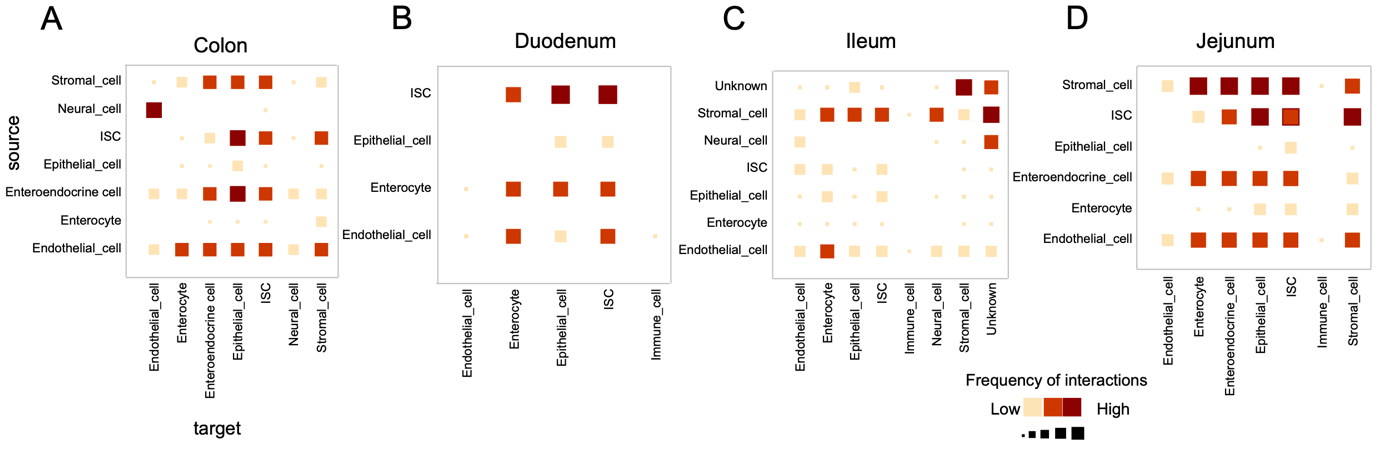


**Figure S28. Heatmap representation showing cellular interaction among the major cell types in four intestinal segments. among the major cell types.** The difference in the inferred EGF signaling pathway network is evaluated by the block sizes and colors, which are proportional to the interaction frequency.

**
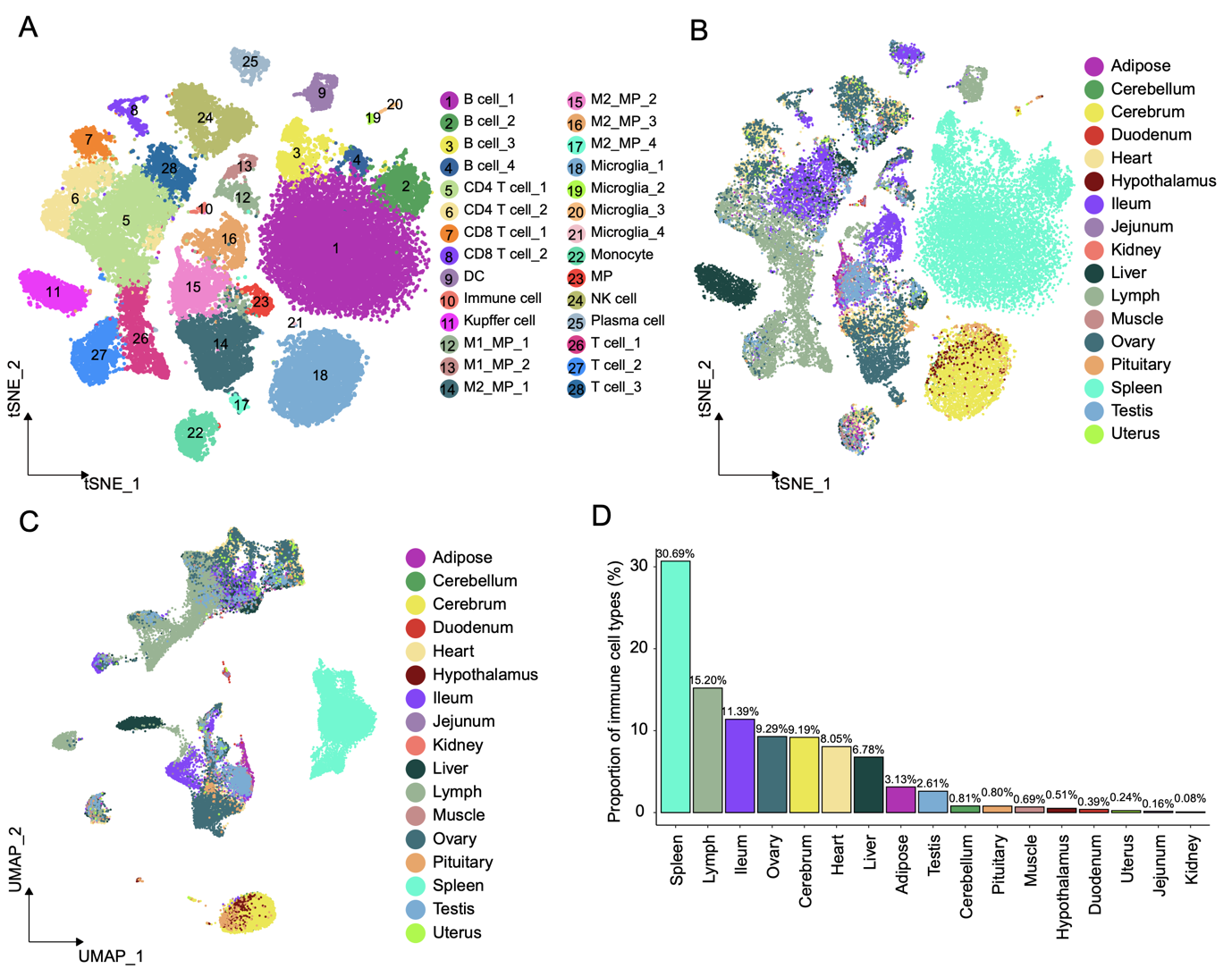
**

**Figure S29. Cell type annotation of immune cell clusters based on snRNA-seq data.**

**(A-B)** t-SNE visualization showing cell distribution according to cell types (left) and tissues (right).

**(C)** UMAP visualization showing cell distribution according to tissues.

**(D)** The proportion of total immune cell types in each tissue.


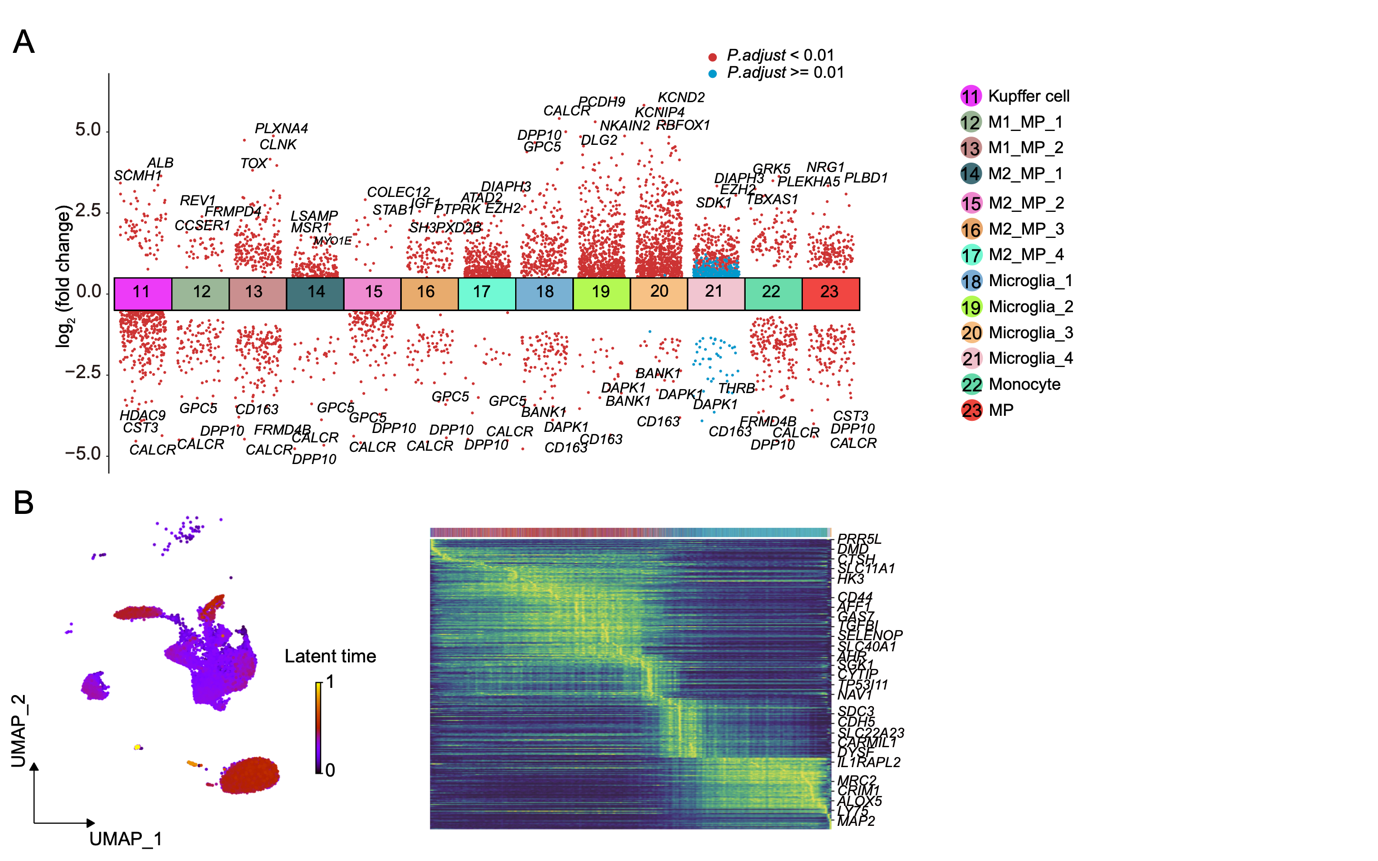


**Figure S30. Diverse transcriptional profiles for each cell cluster in myeloid cell compartments.**

**(A)** Differentially expressed genes for each cell cluster in tissue-resident macrophages and monocytes.

**(B)** UMAP and heatmap showing the pseudotime differentiation trajectories of monocyte and macrophage lineages.


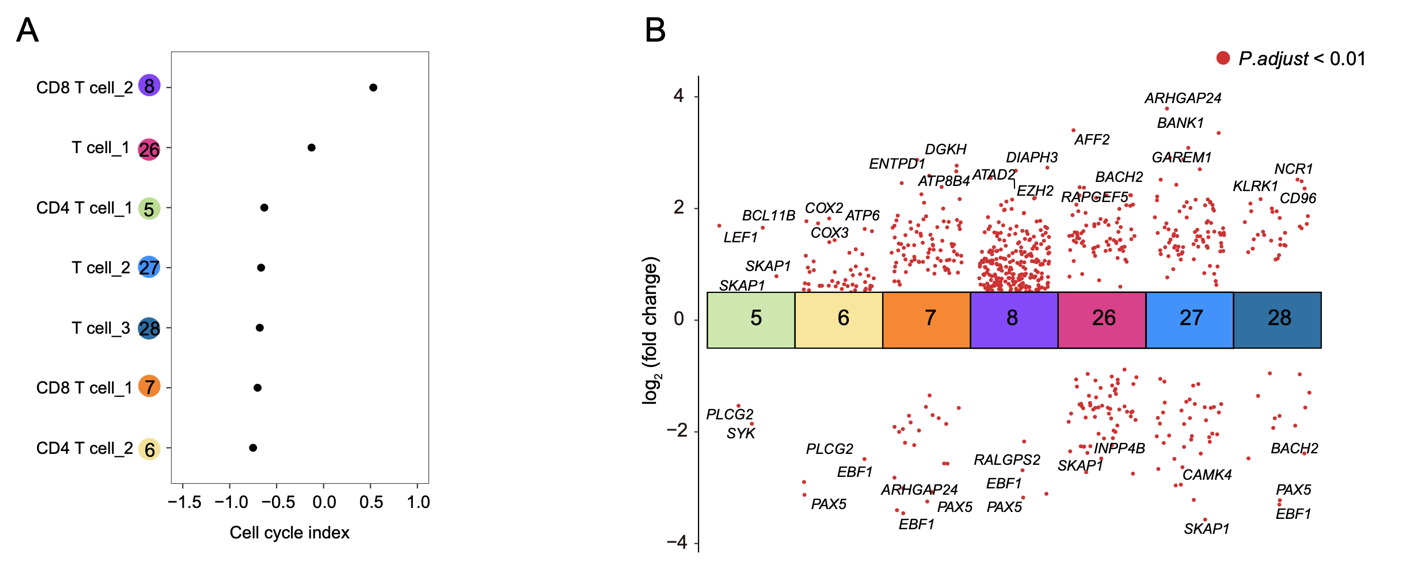


**Figure S31. Diverse transcriptional profiles for each cell cluster in T cell compartments.**

**(A)** Cell cycle indices for various T cell types.

**(B)** Differentially expressed genes for each cell cluster in T cell subpopulations.


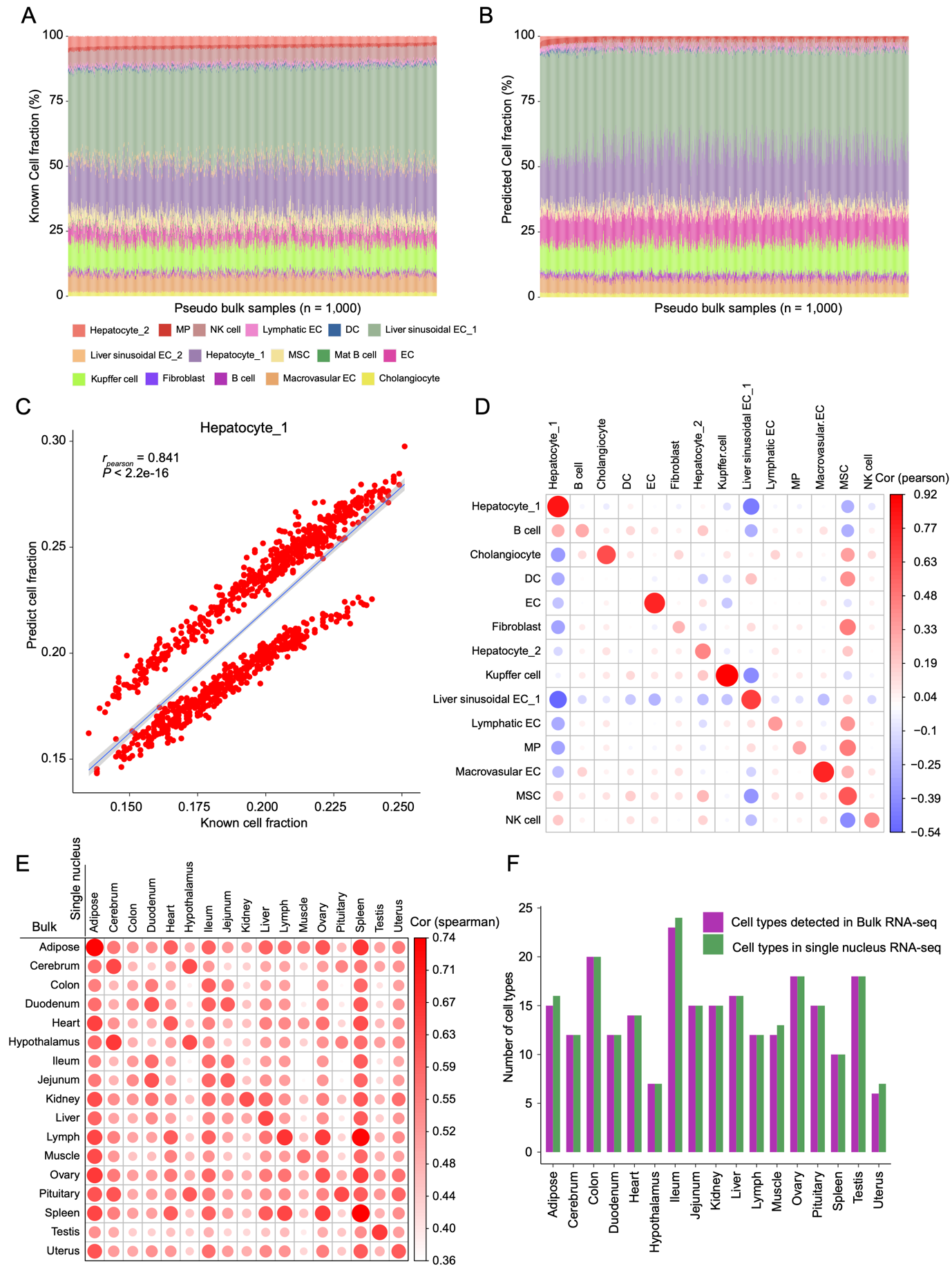


**Figure S32. Correlation between known and deconvoluted cellular composition.**

**(A)** Stacked bar plots showing the known fraction of cell types in the pseudo-bulk liver samples simulated by the SCDC software.

**(B)** Stacked bar plots showing the predicted fraction of cell types by deconvoluting the pseudo-bulk liver samples.

**(C)** Scatter plot of known and predicted cell compositions. The Pearson correlation coefficients are displayed at the top of the panel.

**(D)** Heatmap showing the Pearson correlation coefficients of all putative cell types with the proportion over zero.

**(E)** Heatmap showing the Spearman correlation coefficients of 17 matching tissues between our snRNA-seq and PigGTEx samples.

**(F)** Barplot comparing the number of cell types detected in our snRNA-seq data versus the number of those deconvoluted cell types for 17 PigGTEx tissues.


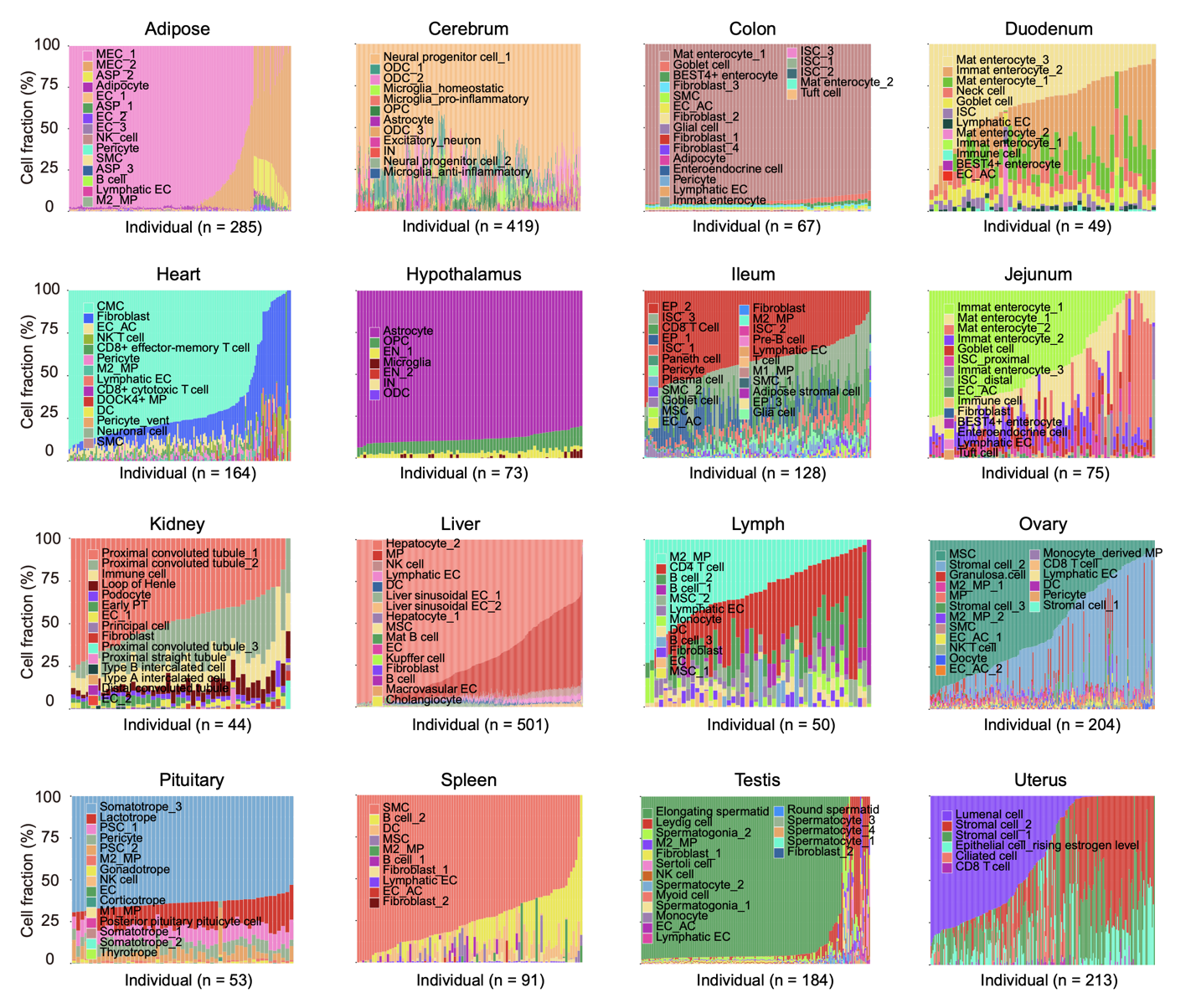


**Figure S33. Stacked bar plots showing the fraction of cell types estimated in PigGTEx RNA-seq samples based on our snRNA-seq reference matrix in 16 tissues.** These tissues contain the adipose, cerebellum, cerebrum, colon, duodenum, heart, hypothalamus, ileum, jejunum, kidney, liver, lymph, ovary, pancreas, pituitary, spleen, testis, and uterus.


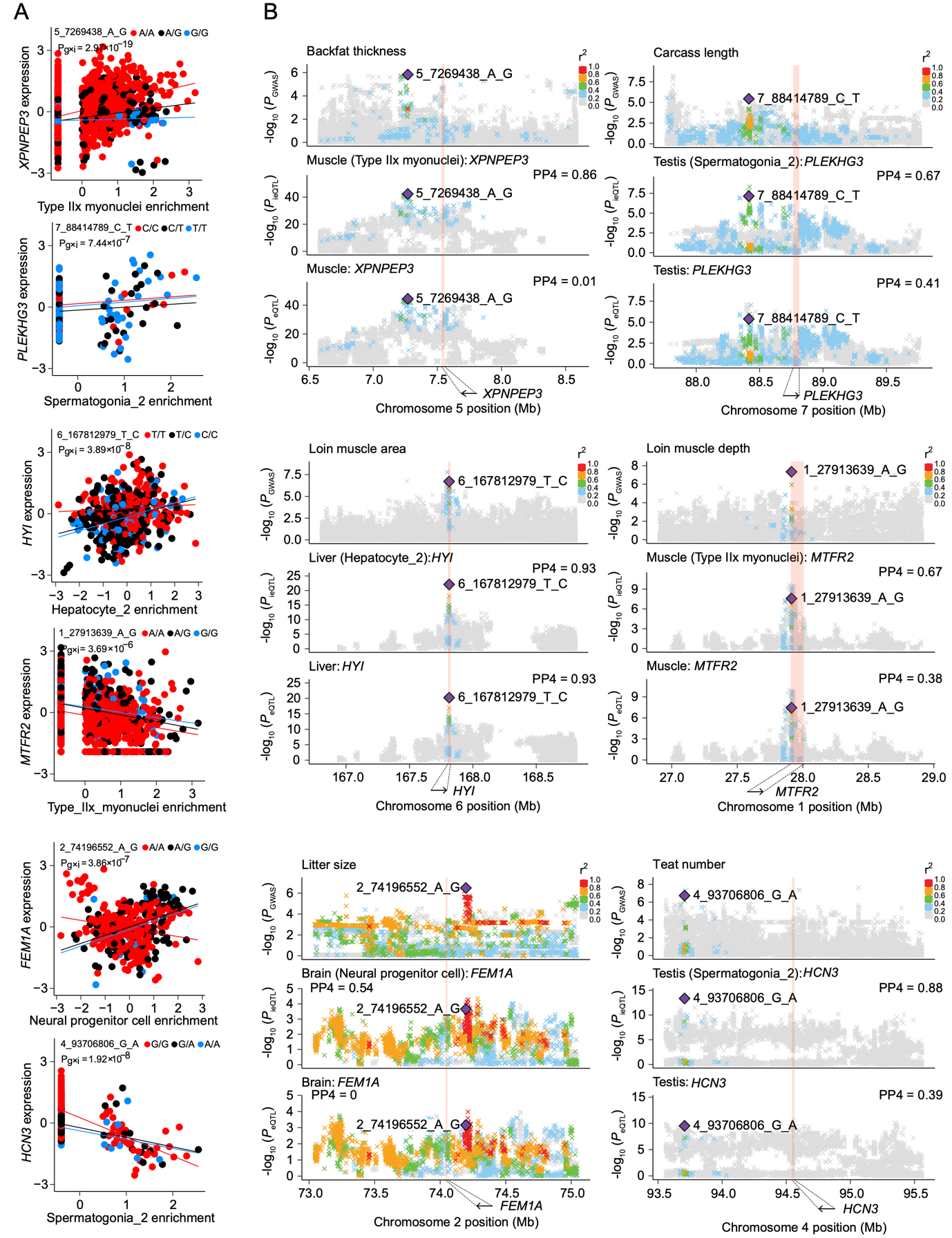


**Figure S34. Cell-type-specific effects of ieQTL on gene expression and complex traits.**

**(A)** Six examples showing cell-type-specific effects in different cell types. Each point represents an individual and is colored by three genotypes. Both gene expression levels and cell type enrichment values are inverse normal transformed across samples. The lines are fitted by a linear regression model using the geom_smooth function from ggplot2 (v3.3.2) in R (v4.0.2). Orange shading indicates gene position and the black arrow (bottom) indicates gene expression direction.

**(B)** Aligned Manhattan plots of pig GWAS, ieQTL, and eQTL at the six representative loci for different traits. SNPs are colored according to the magnitude of linkage disequilibrium (*r*^2^) between adjacent SNP pairs. Orange shading indicates gene position and the black arrow (bottom) indicates gene expression direction.


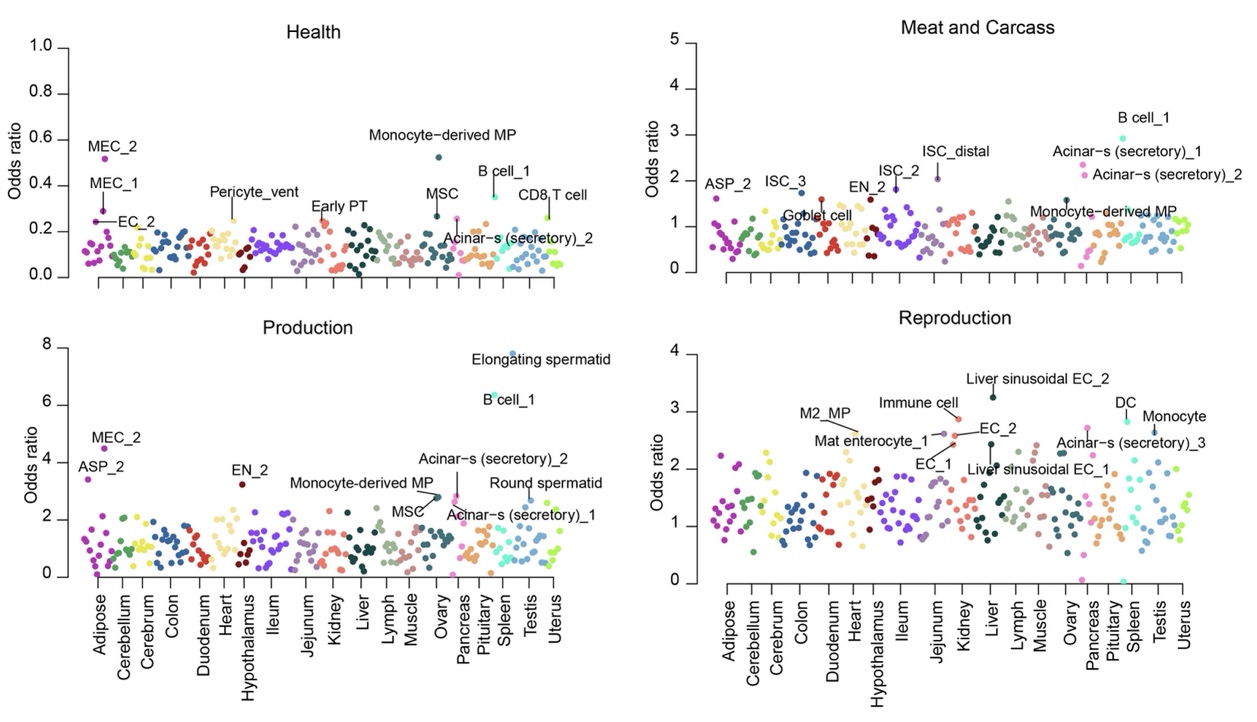


**Figure S35. Dot plots showing the significance of enrichment for four main trait categories.** The exterior category was discarded due to the low sample size (n = 6). Each circle represents a cell type. The top 10 most highly associated cell types are labeled for each trait.


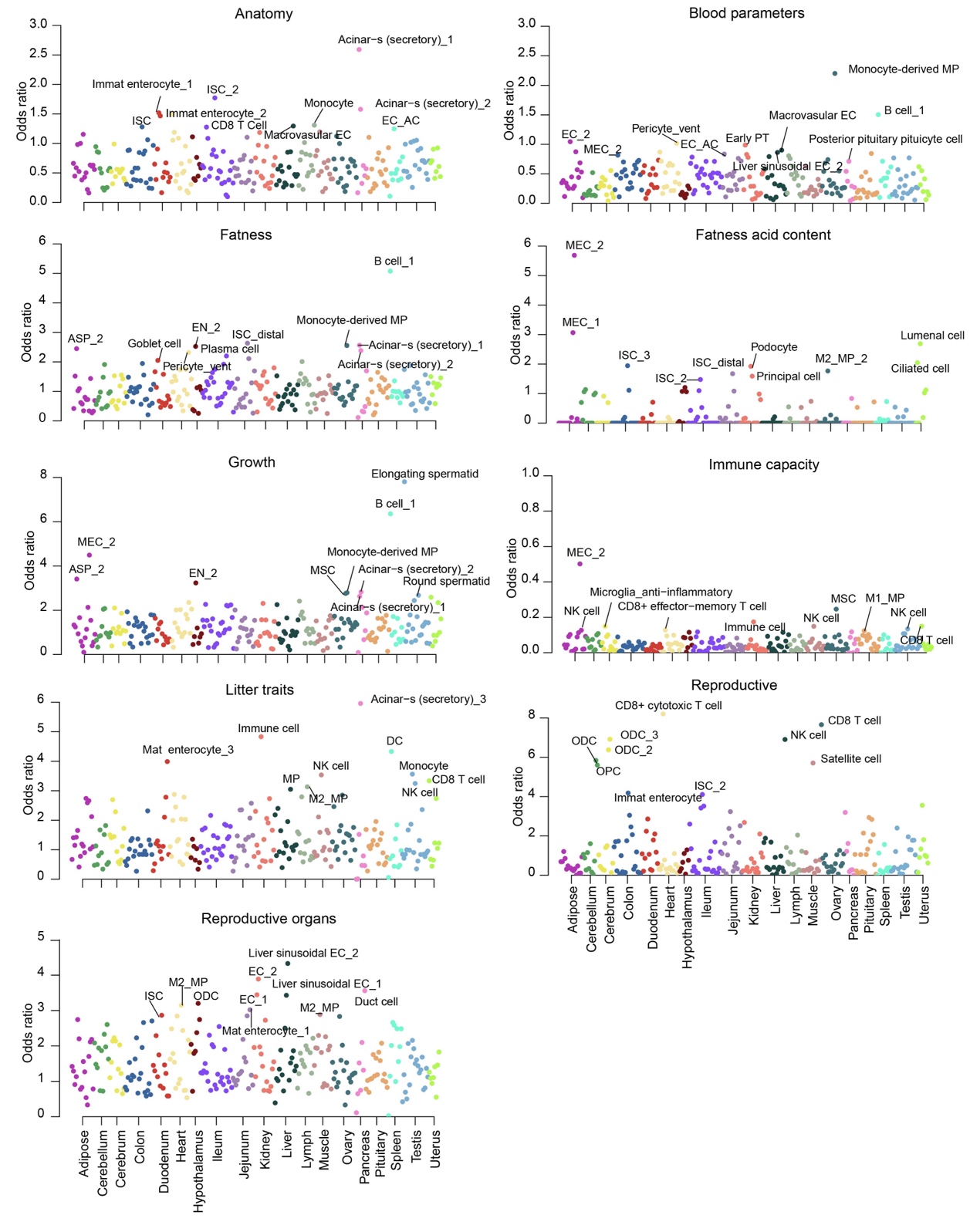


**Figure S36. Dot plots showing the significance of enrichment for nine trait subcategories.** Each circle represents a cell type. The top 10 most highly associated cell types are labeled for each trait.


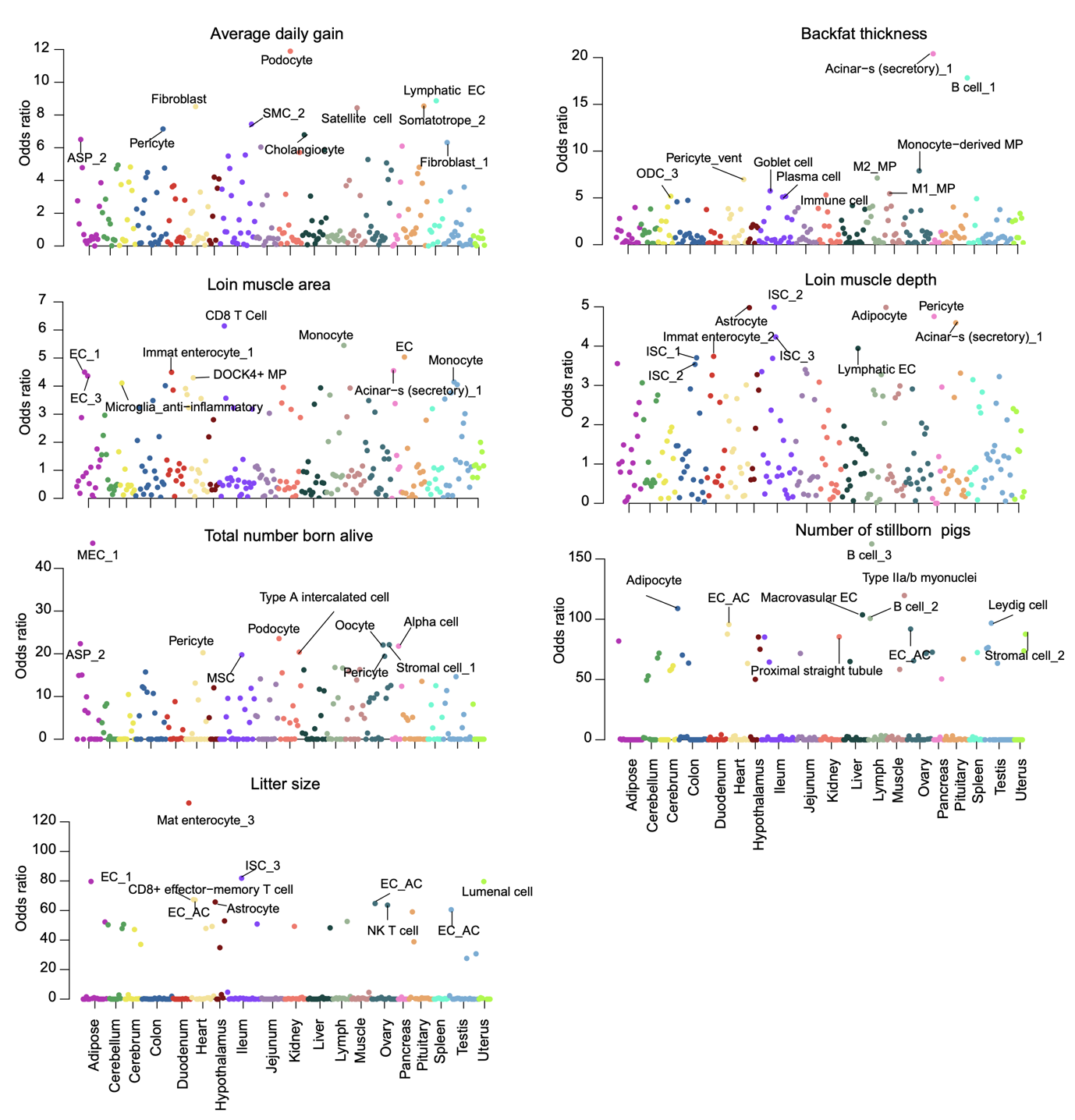


**Figure S37. Dot plots showing the significance of enrichment for seven representative traits.** These traits contain average daily gain, backfat thickness, loin muscle area, loin muscle depth, total number born alive, number of stillborn pigs, and litter size. Each circle represents a cell type. The top 10 most highly associated cell types are labeled for each trait.


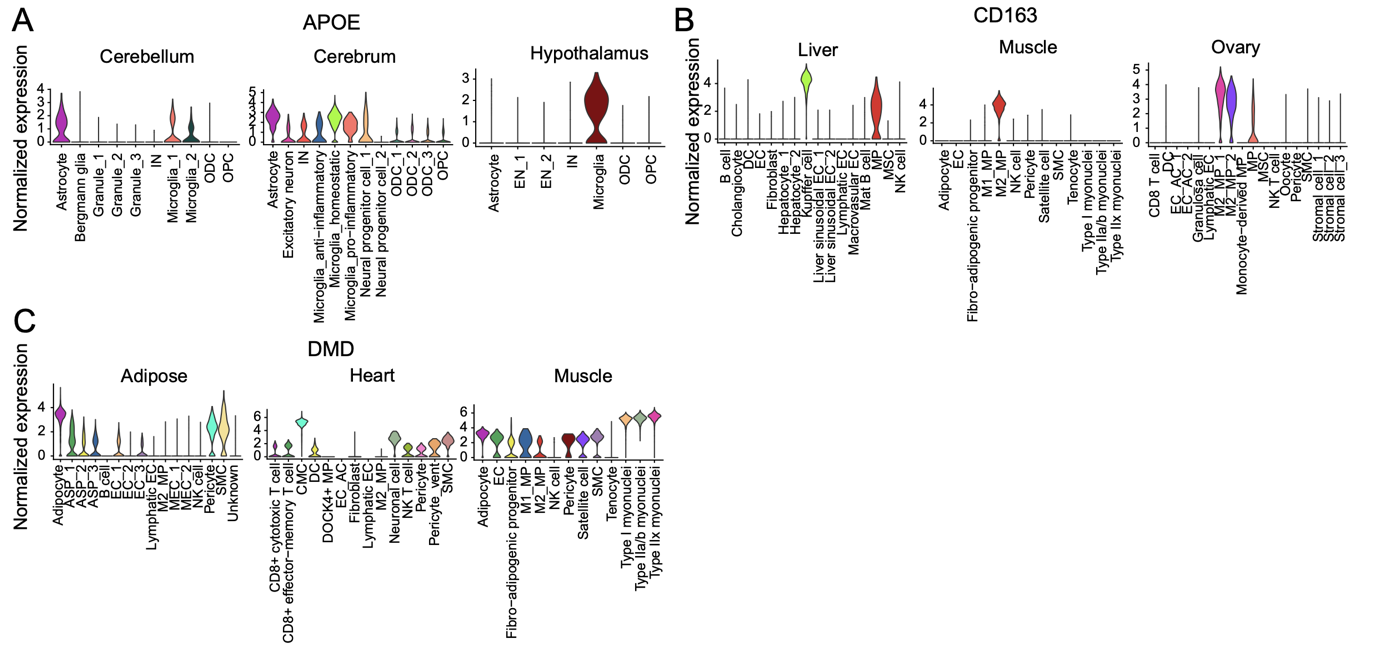


**Figure S38. Violin plots showing the expression levels of the three representative genes.**

**(A)** Cell-type-specific expression patterns of *APOE* in the cerebellum, cerebrum, and hypothalamus. The *APOE* gene is a key candidate associated with hyperlipidemia/atherosclerosis from the OMIA database.

**(B)** Cell-type-specific expression patterns of *CD163* in the liver, muscle, and ovary. The *CD163* gene is an essential receptor linked to resistance/susceptibility to the porcine reproductive and respiratory syndrome (PRRS) virus from the OMIA database.

**(C)** Cell-type-specific expression patterns of *DMD* in the adipose, heart, and muscle. The *DMD* gene plays a vital role in muscular dystrophy from the OMIA database.


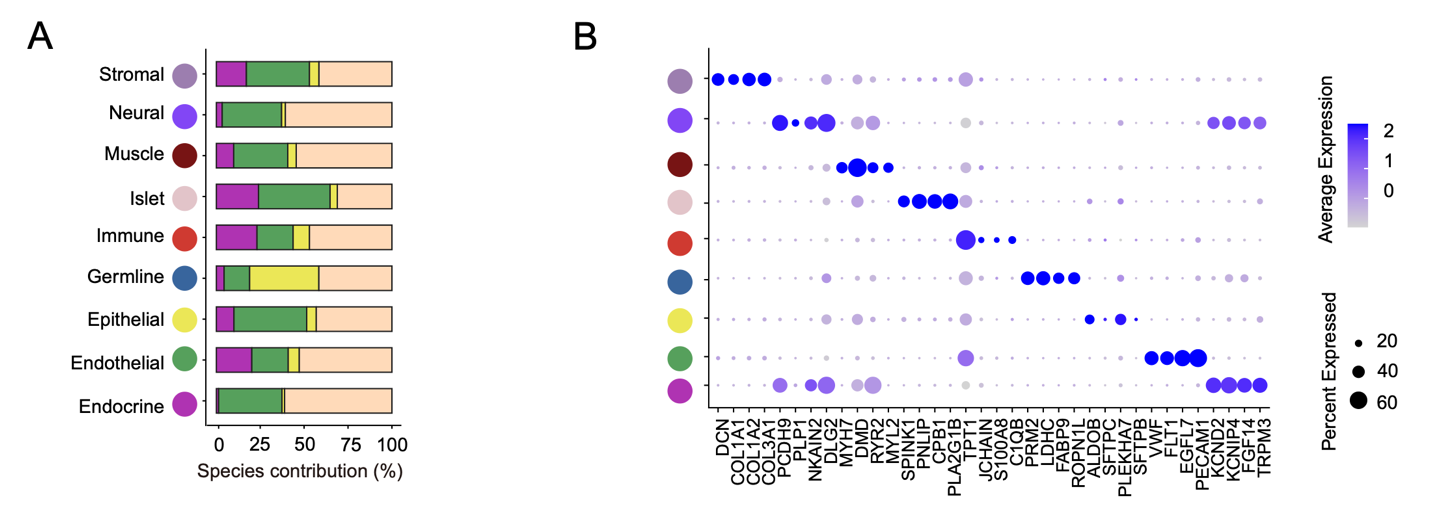


**Figure S39. Characterization of species contribution and marker genes for the nine lineages in the integrated cell map from human, monkey, mouse and pig.**

**(A)** Bar plot showing the percentage of cells from the four species.

**(B)** Dot plot showing marker genes of each lineage.


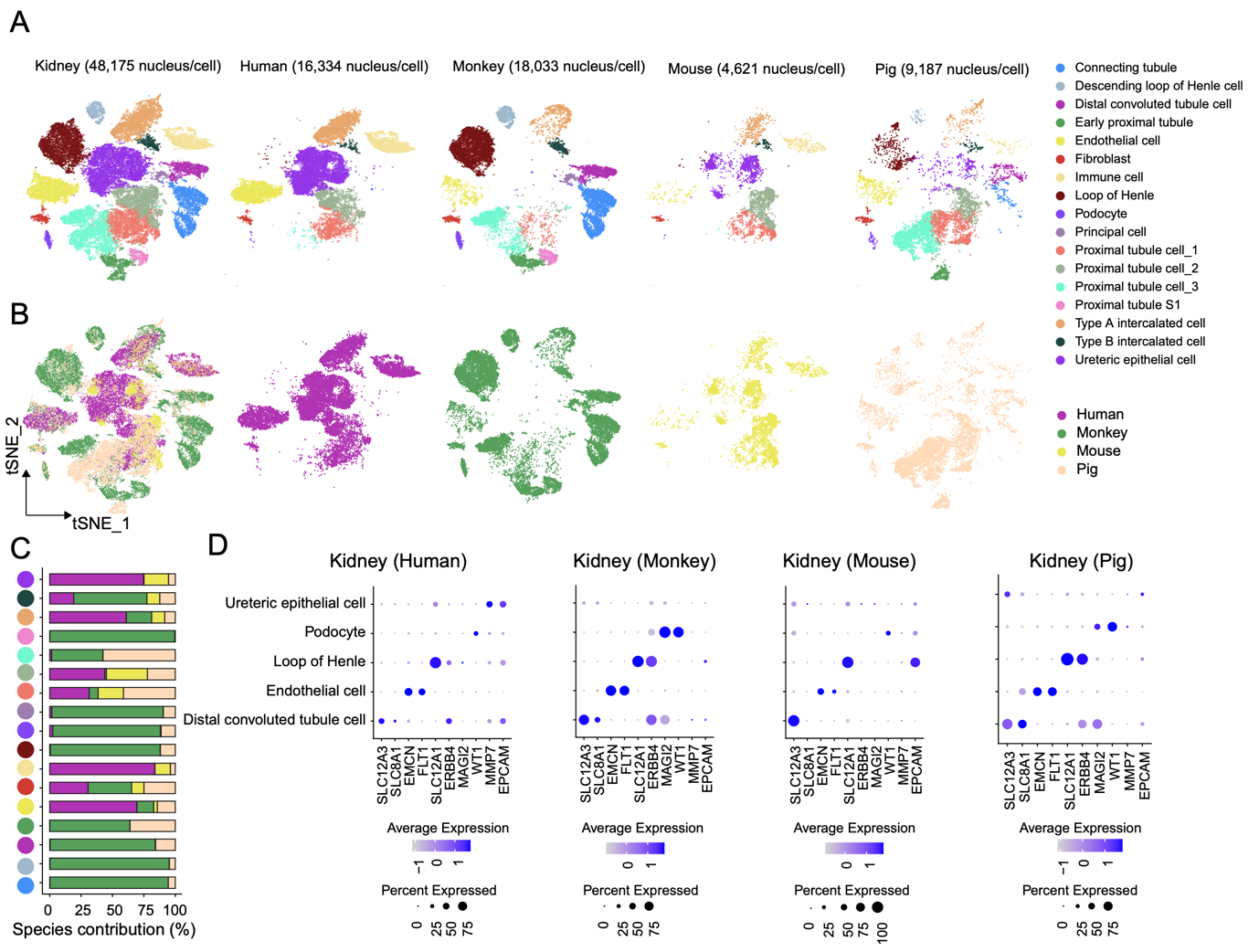


**Figure S40. Integrated cell atlas by combining single-nucleus/cell RNA-seq data in kidney from human, monkey, mouse and pig.**

**(A-B)** t-SNE showing the cell landscape according to cell types (upper) and species (lower).

**(C)** Bar plot showing the percentage of cells from the four species.

**(D)** Dot plot showing marker genes of each cell type.


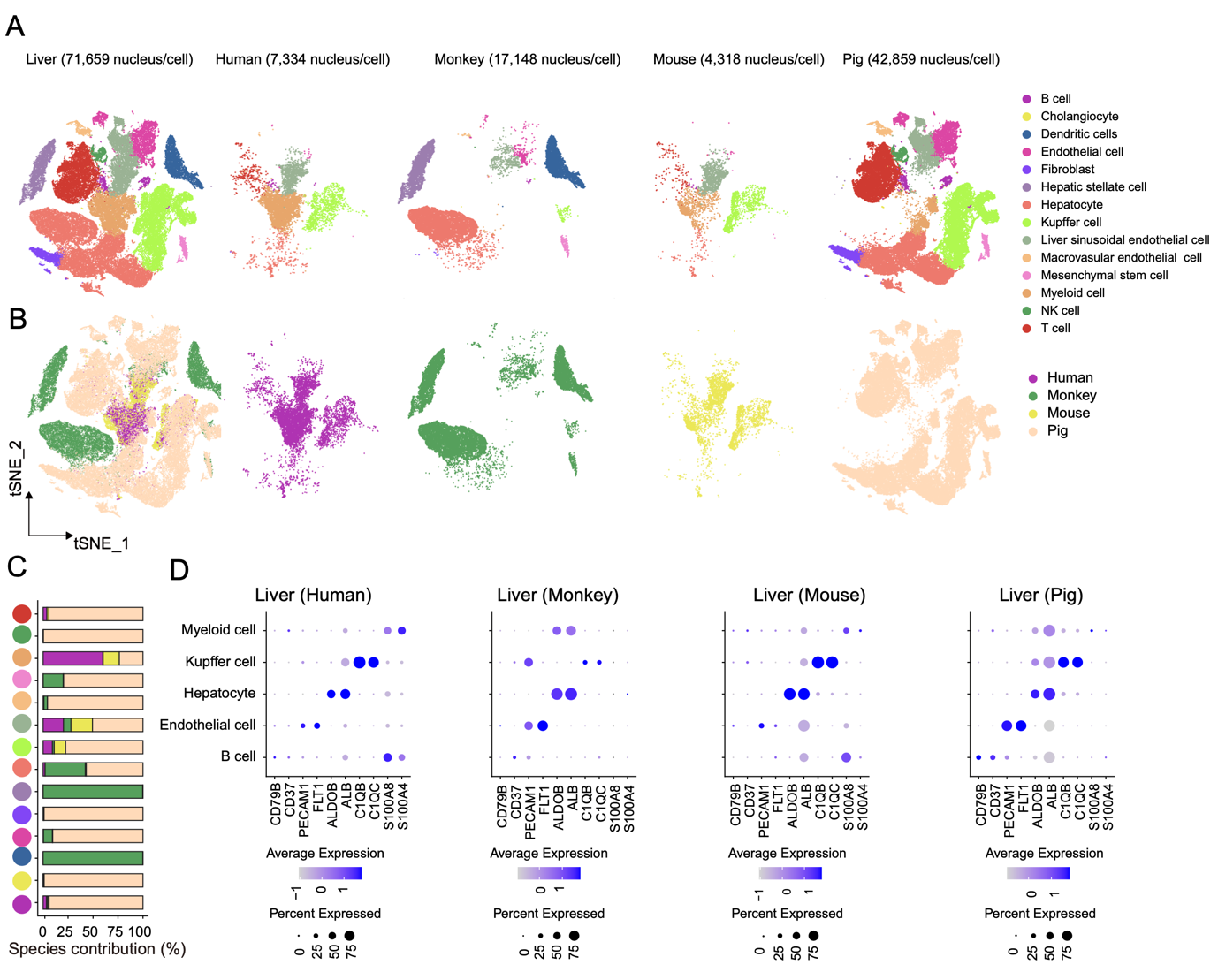


**Figure S41. Integrated cell atlas by combining single-nucleus/cell RNA-seq data in liver from human, monkey, mouse and pig.**

**(A-B)** t-SNE showing the cell landscape according to cell types (upper) and species (lower).

**(C)** Bar plot showing the percentage of cells from the four species.

**(D)** Dot plot showing marker genes of each cell type.


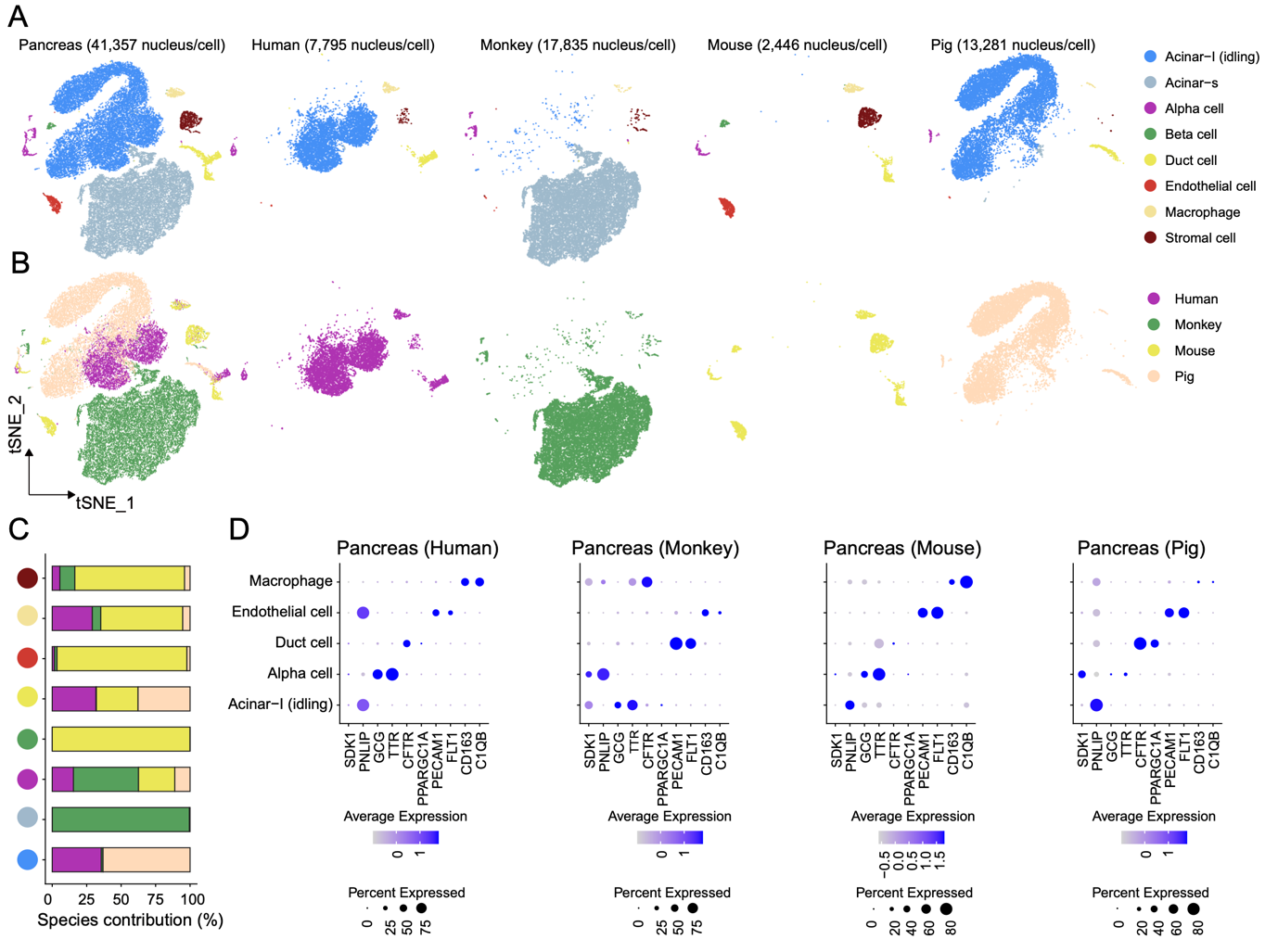


**Figure S42. Integrated cell atlas by combining single- nucleus/cell RNA-seq data in pancreas from human, monkey, mouse and pig.**

**(A-B)** t-SNE showing the cell landscape according to cell types (upper) and species (lower).

**(C)** Bar plot showing the percentage of cells from the four species.

**(D)** Dot plot showing marker genes of each cell type.


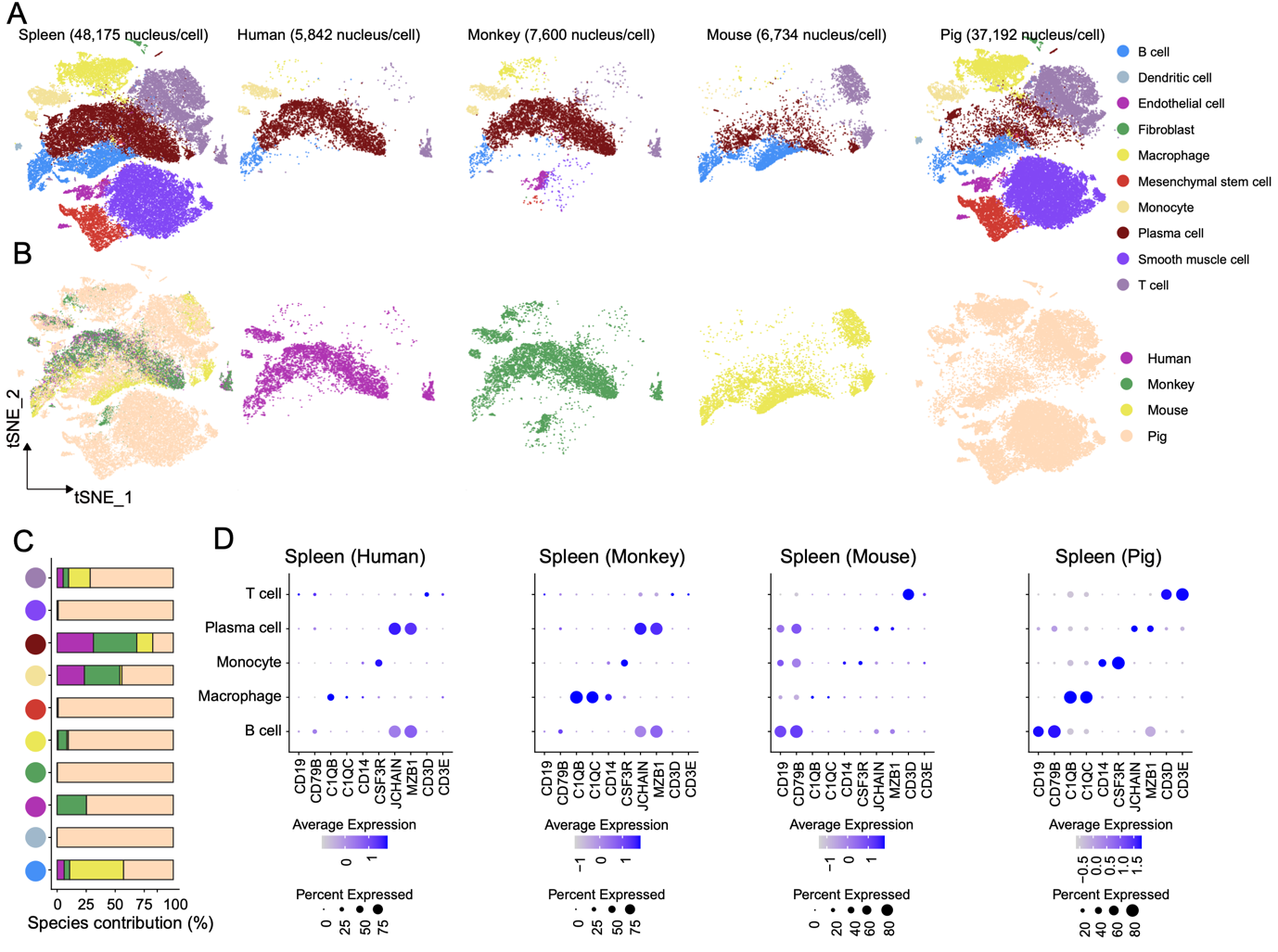


**Figure S43. Integrated cell atlas by combining single- nucleus/cell RNA-seq data in spleen from human, monkey, mouse and pig.**

**(A-B)** t-SNE showing the cell landscape according to cell types (upper) and species (lower).

**(C)** Bar plot showing the percentage of cells from the four species.

**(D)** Dot plot showing marker genes of each cell type.


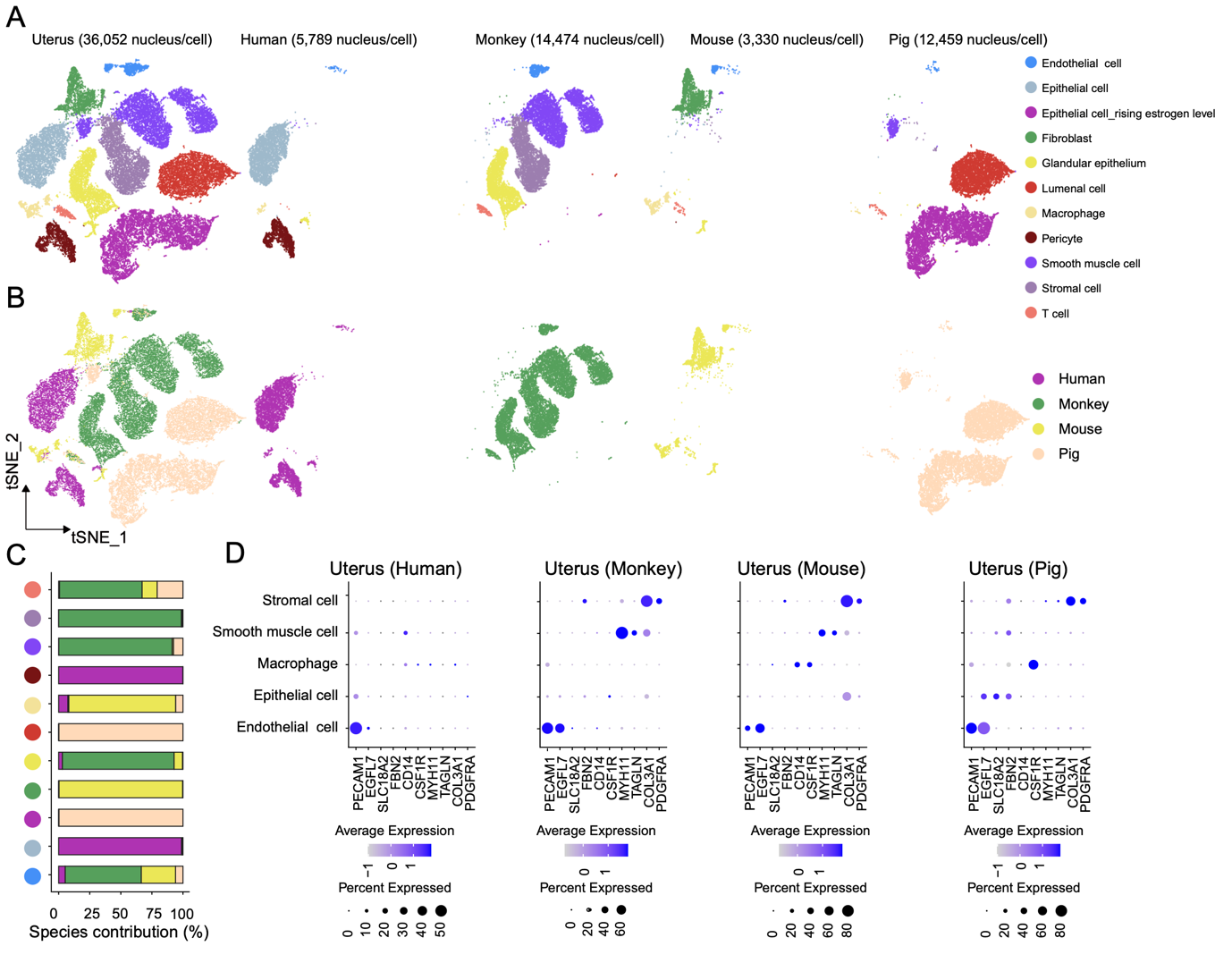


**Figure S44. Integrated cell atlas by combining single-nucleus/cell RNA-seq data in uterus from human, monkey, mouse and pig.**

**(A-B)** t-SNE showing the cell landscape according to cell types (upper) and species (lower).

**(C)** Bar plot showing the percentage of cells from the four species.

**(D)** Dot plot showing marker genes of each cell type.


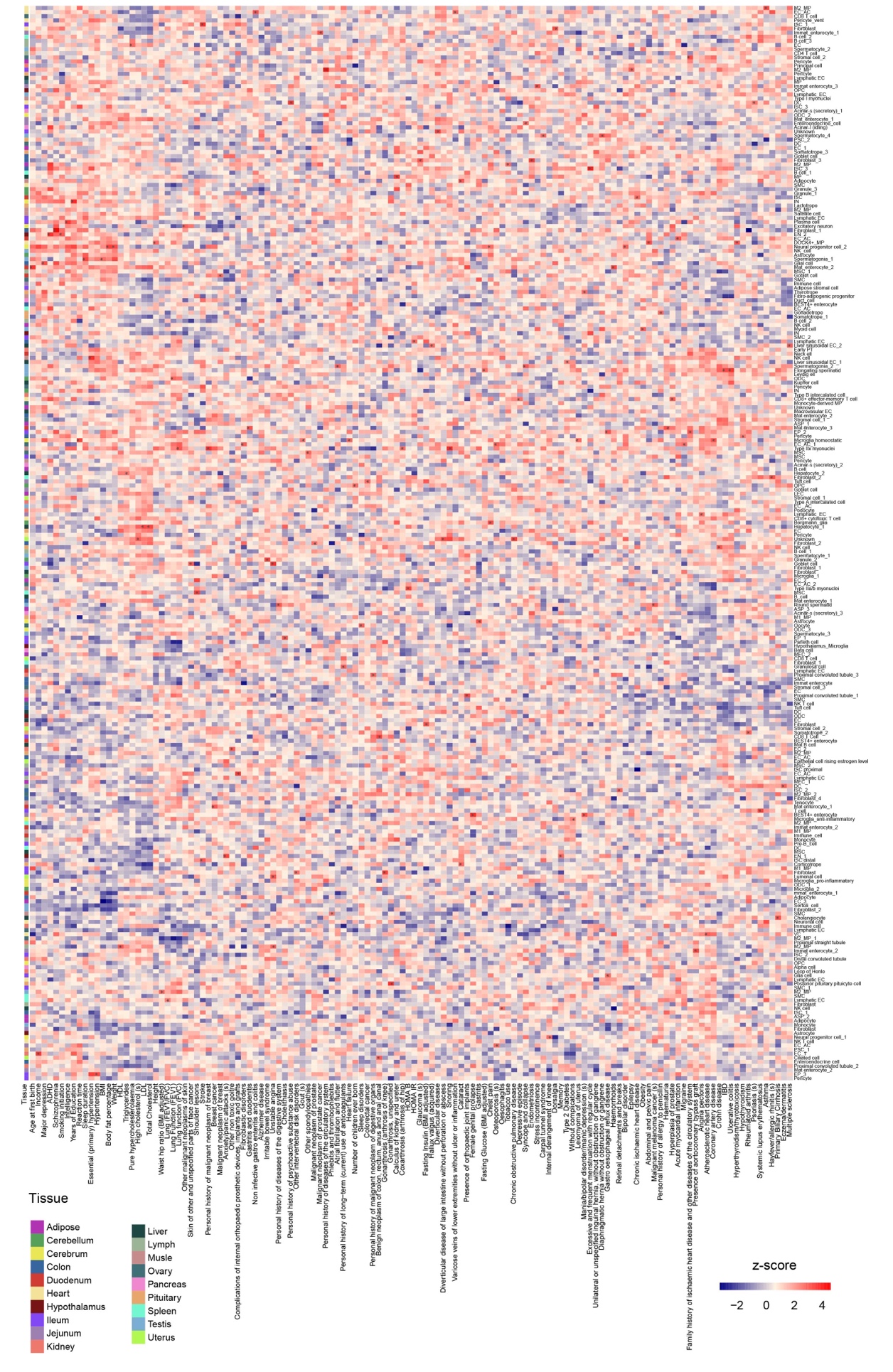


**Figure S45. Global cell-type-trait associations in humans.** Heatmap showing enrichment of cell types associated with complex traits and diseases from genome-wide association studies in humans. Stratified linkage disequilibrium score regression (LDSC) analysis was performed using GWAS summary statistics for 137 phenotypes. All signficant associations were marked by asterisk.

Supplementary Tables:

**Table S1: Canonical marker genes from the literature in 19 tissues.**

**Table S2: Significant genes for each cell cluster in 19 pig tissues.** pct.1 is the percentage of cells in the cluster where the gene is detected, while pct.2 is the percentage of cells on average in all the other clusters where the gene is detected.

**Table S3: List of the cell cycle markers.**

**Table S4: Putative driver genes from satellite cells to myofiber in the skeletal muscle tissue.**

**Table S5: The complete list of all significant ligand-receptor pairs and pathways across the four intestine segments.**

**Table S6: Detailed information on 268 metaGWAS in pigs.**

**Table S7: Coloc results of cell type ieQTL and eQTL with PP4 ≥ 0.50.**

**Table S8: GWAS results from 26,000 individuals.**

**Table S9: Summary of 137 GWAS in humans.** The information of complex traits and diseases in the 6A sheet was collected from published papers, while these in the 6B sheet were downloaded from <https://gwas.mrcieu.ac.uk/datasets/>.

**Table S10: Association of GWAS traits and human genetic diseases with pig cell types.** Heritability enrichment was calculated as the proportion of trait heritability contributed by SNPs in the annotation over the total proportion of SNPs in that annotation using the LDSC method, and the estimated z-score was used for visualizing the enrichment results.
